# Shotgun metagenomics reveals distinct skin microbial species in allergen-sensitized individuals

**DOI:** 10.1099/mgen.0.001527

**Published:** 2025-12-03

**Authors:** Matilda Riskumäki, Matti O. Ruuskanen, Kuunsäde Mäenpää, Lasse Ruokolainen, Mika J. Mäkelä, Pekka Jousilahti, Erkki Vartiainen, Noora Ottman, Tiina Laatikainen, Tari Haahtela, Harri Alenius, Nanna Fyhrquist, Hanna Sinkko

**Affiliations:** 1 Human Microbiome Research Program, University of Helsinki, Helsinki, Finland; 2 Department of Computing, University of Turku, Turku, Finland; 3 Department of Biosciences, University of Helsinki, Helsinki, Finland; 4 Skin and Allergy Hospital, Helsinki University Hospital, University of Helsinki, Helsinki, Finland; 5 Department of Public Health, Finnish Institute for Health and Welfare, Helsinki, Finland; 6 National Food Institute, Technical University of Denmark, Kgs Lyngby, Denmark; 7 Institute of Public Health and Clinical Nutrition, University of Eastern Finland, Kuopio, Finland; 8 Institute of Environmental Medicine, Karolinska Institutet, Stockholm, Sweden; 9 Department of Public Health Sciences, Karlstad University, Karlstad, Sweden

**Keywords:** allergic sensitization, environmental exposure, Karelia allergy study, metagenomics, skin microbiome

## Abstract

The Karelian region, which spans the border between Finland and Russia, presents distinct environmental exposures and lifestyles on either side of the governmental border. In the more urbanized Finnish Karelia, allergic diseases are markedly more prevalent than in the more rural Russian Karelia. Prior studies, based on amplicon sequencing, have demonstrated major differences in skin microbiotas between the two populations. However, compositional differences in microbiota between sensitized and non-sensitized (NS) individuals have not been characterized. Here, in a selected population of 112 allergen-sensitized and NS adolescents, we used shotgun metagenomics to characterize the prokaryotic, eukaryotic and viral species in the skin potentially involved in allergic sensitization via distinct environmental exposures. In the more urban Finnish Karelia, the microbiome species composition was associated with IgE-mediated allergen sensitization status, while in the more rural Russian Karelia, the composition was associated with exposure to furry pets. Finnish participants showing high IgE-mediated sensitization to common allergens (allergen-specific IgE >7.5 kU/L) had less *Cutibacterium acnes* and *Malassezia* in their skin and displayed weaker interconnectedness of the microbial co-occurrence network compared with NS participants. Moreover, *Malassezia restricta* strain-level differences were related to allergen sensitization in both Finnish and Russian participants. In summary, we found distinct skin microbiomes between allergen-sensitized and NS participants and tracked the bacterial and fungal species associated with the degree of allergic sensitization in the more urbanized part of the Karelian region. These findings provide new insights into the factors that shape the human skin microbiome and influence allergic diseases.

**Impact Statement**

The prevalence of allergic diseases has increased worldwide in the past decades along with rapid urbanization. In urban environments, deprived of green spaces and natural environments, loss of biodiversity is apparent. A decline in exposure to biodiverse environments has resulted in a deprived composition of micro-organisms living in human bodies. This community, i.e. human microbiome, plays a crucial role in immune modulation, influencing allergy risk and overall health. On skin, the microbiome receives direct and continuous input from the external environment, providing a proxy of environmental microbial exposure. We explored the relation of skin microbiome, living environment and allergic sensitization in Finnish and Russian Karelia – regions that are geo-climatically similar but distinct in environmental exposures and allergy prevalence. Improved knowledge on how the interaction of skin microbiome and living environment influences the risk of developing allergic diseases will enable planning of urban areas that preserve natural biodiversity to benefit human health.

## Data Summary

Sequencing data of the quality filtered and trimmed microbial reads have been uploaded to the NCBI Sequence Read Archive with accession number PRJNA1155269. The script for the statistical analyses executed in R is included as Additional file 1. Relative abundance and read count tables, including taxonomies from MetaPhlan4 and MetaPhlAn3 classification, as well as metadata, are included as Additional files 2, 3, 4, 5 and 6, respectively. Maximum likelihood phylogeny of *Malassezia restricta* strains and the code for the tree construction are included as Additional files 7 and 8, respectively. StrainPhlAn and IQ-TREE commands for strain identification, multiple sequence alignment, likelihood-mapping and maximum likelihood phylogenetic tree construction are included as Additional file 8. The Additional files 1-8 are available through Figshare at https://doi.org/10.6084/m9.figshare.30095587.v1 [1].

## Background

Rapid urbanization and modernization after World War II have dramatically reduced human exposure to biodiverse and natural environments. Concurrently, the prevalence of allergic diseases has increased worldwide [2,3]. The biodiversity hypothesis suggests that a decline in exposure to diverse natural environments results in human microbiome deprivation, disturbed immune regulation and increased risk of allergic conditions and inflammatory disorders [4]. The skin microbiome is strategically positioned at the interface between the body and the environment, receiving direct and continuous input from the external environment [5]. Environment and lifestyle shape the skin microbiome, which, alongside other human microbiomes, plays an important role in modulating host immune regulation, influencing allergy risk and overall health [5,7].

The Karelian region, spanning the border between Finland and Russia, offers contrasting environmental exposures to the Finnish and Russian populations despite their close geographical proximity. Since the 1950s, the socioeconomic gap between North Karelia in Finland (Finnish Karelia) and the Republic of Karelia in Russia (Russian Karelia) has been widening. While rapid urbanization occurred in Finland, many households in Russian Karelia continued their small-scale agricultural lifestyle [8]. In Finnish Karelia, allergic diseases are significantly more prevalent than in Russian Karelia and have increased across generations, while in Russian Karelia, the prevalence has remained low [8,9]. The growing allergy gap is not explained by genetic differences, air pollution or exposure to common environmental chemicals [10,11]. Instead, it could be attributed to distinct environmental exposures and contrasting lifestyles, which influence the human microbiome, immune regulation and skin barrier integrity [3,12,14]. Commensal microbes in the skin support an intact skin barrier, which acts as a protective layer between the body and the environment [5,14].

Taxonomic genus-level differences in the skin microbiota between the populations in Finnish and Russian Karelia have been demonstrated by targeting parts of the bacterial 16S rRNA and fungal internal transcribed spacer (ITS) gene regions [9]. While single-gene amplicon sequencing is cost-effective, the biologically important species- and strain-level classification remains poor [15,16]. Using the genus-level taxonomies, the previous study on the cohort (8) did not find a difference in the skin microbiota between allergen-sensitized and non-sensitized (NS) individuals. Overall, the information on microbial species involved in IgE-mediated allergen sensitization or protection from it is scarce, especially in the skin. The human skin metagenome has not been extensively studied.

Using whole metagenome shotgun (WMS) sequencing, we provide species-level classification of *bacteria*, *archaea*, fungi and DNA viruses to better understand the crosstalk among the environment, skin microbiome and allergic sensitization. We examined the skin microbiome of Finnish and Russian adolescents in Karelia and associated the microbial communities with IgE-mediated allergic sensitization and environmental exposure. Improved knowledge may help identify nature-based solutions in densely populated areas to preserve natural biodiversity and benefit human health.

## Methods

### Sample collection

**Fig. 1.**
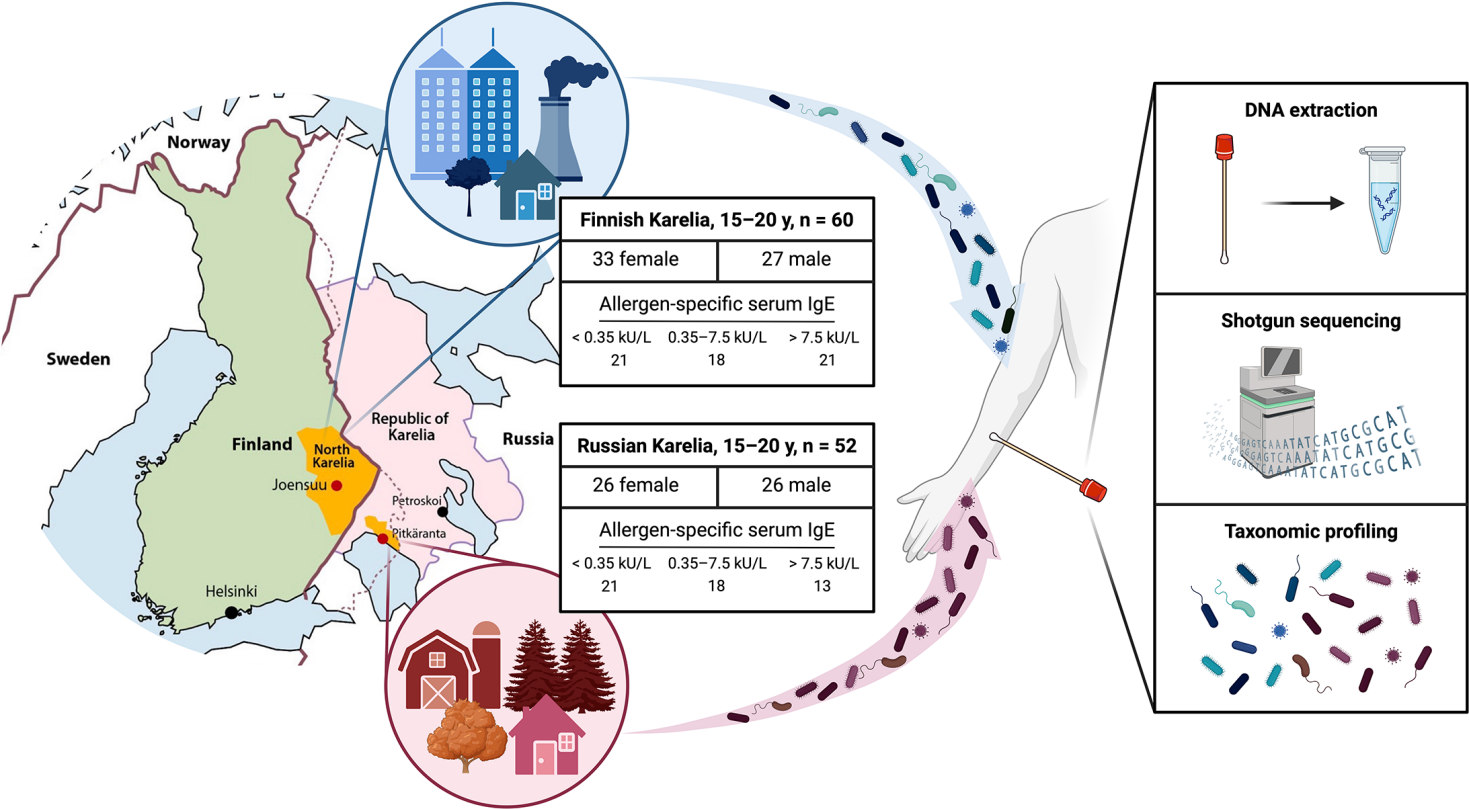
Study design. The subgroup of study participants (*n*=112, age=15–20) was selected from a cohort that had been recruited in Finnish and Russian Karelia in 2003 and re-examined in 2010–2012. Finnish and Russian Karelia present distinct environmental exposures and lifestyles despite their geographical proximity. Skin swab and serum samples were used for microbiome and sIgE analyses. The subgroup included equal numbers of age- and sex-matched allergen-sensitized and NS participants from Finnish and Russian Karelia, respectively. Differences in microbiome compositions were studied between Finnish and Russian Karelia, as well as between the Finnish allergen-sensitized and NS participants. The image was created in BioRender, Riskumäki, M. (2025 https://BioRender.com/k24j646. The map was adapted with permission from Haahtela *et al*. [8].

The study participants were part of a study cohort initially recruited in 2003 in North Karelia, Finland (Finnish Karelia), and the Republic of Karelia, Russia (Russian Karelia) [17]. The participants were re-examined in 2010–2012, and the samples collected then were used in the present subgroup study. The original study cohort and sample collection have been described in detail earlier [9]. This study consisted of a subgroup (*n*=112) of the original study cohort, including 60 Finnish (33 female, 27 male) and 52 Russian (26 female, 26 male) participants (Fig. 1). The participants were aged between 15 and 20 years at the time of the follow-up. The subgroup selection aimed to include equal numbers of age- and sex-matched allergen-sensitized and NS participants from Finnish and Russian Karelia, respectively, to facilitate comparison of the skin microbiomes between groups. Therefore, the subgroup did not represent the differences in allergic sensitization prevalence between Finnish and Russian Karelia described in the original study cohort [9].

For the present analyses, skin swab samples collected from the volar forearm were included, along with clinical data and lifestyle information obtained through self-administered questionnaires. Serum immunoglobulin E (sIgE) against a combination of eight common inhalant allergens (timothy grass, birch, mugwort, horse, cat and dog, house dust mite *D. pteronyssinus* and a common mould *Cladosporium herbarum*) was measured using Phadiatop^®^. The participants were divided into three allergic sensitization categories according to specific sIgE levels against the allergen combination: NS (allergen-specific sIgE <0.35 kU/L), sensitized intermediate (SI; allergen-specific sIgE=0.35–7.5 kU/L) and highly sensitized (HS; allergen-specific sIgE >7.5 kU/L) (Figs 1 and S1, available in the online Supplementary Material). If the specific sIgE level against the allergen combination was above 0.35 kU/L, specific sIgE against each of the eight inhalant allergens was measured separately. Thus, for NS participants, only the allergen combination-specific sIgE levels were available, and the specific sIgE levels against each separate allergen were set to zero (0) for further analyses.

### DNA extraction and shotgun sequencing

DNA was extracted from the skin swab samples as described by Mäenpää *et al*. [18]. In short, skin swabs were incubated in lysis buffer (MasterPure Yeast DNA Purification kit, Epicentre) with lysozyme (ReadyLyse Lysozyme solution, Epicentre) and bead beaten (BeadBug, Sigma-Aldrich). The lysis was followed by protein precipitation and removal, then DNA precipitation and washing (PureLink Genomic DNA Kit, Invitrogen) and finally DNA elution (Buffer EB, Qiagen). Library preparation and shotgun sequencing were provided by the Biomedicum Functional Genomics Unit at the Helsinki Institute of Life Science and Biocenter Finland (University of Helsinki). Libraries were prepared with the NEBNext Ultra II FS kit (New England Biolabs), and between 7 and 13 PCR cycles were applied depending on the sample-specific DNA concentration. The libraries were sequenced in one lane on an S4 flow cell in the Illumina NovaSeq6000 using paired-end read length of 2×150 bp.

### Sequencing data filtering and taxonomic, strain-level and functional classification

The adapter sequences were removed from the reads using Cutadapt (v3.4) [19], followed by quality trimming and filtering, and removal of human genome mapping reads using KneadData (v0.10.0), which utilizes Trimmomatic and Bowtie2 [20,21]. Reads were quality filtered and trimmed using KneadData and Trimmomatic default options (phred33 and SLIDINGWINDOW:4:20 MINLEN:50, respectively). Trimmed reads were then mapped against the human genome assembly (hg37), and the mapping reads were removed using Bowtie2. Quality of the trimmed and filtered reads was analysed using FastQC within KneadData with the option RUN-FASTQ-END [22]. Identification of prokaryotes and eukaryotes was performed using MetaPhlAn4 (v4.0.3 and database vJan21) [23]. Relative abundance and count data of the lowest rank, that is, the species-level genome bins (species based on reference genomes and metagenome-assembled genomes), were used for further analyses. DNA viruses were identified separately with MetaPhlAn3 (v.3.1.0) [23,24]. For the viral profiles, the species-level relative abundance and count data were used for further analyses. The taxonomic profiles (prokaryotes and eukaryotes) and viral profiles were converted into TreeSummarizedExperiment (v2.8.0) objects and further analysed in R (v4.3.1) [25,26].

Per-sample functional gene families and functional pathways were profiled using HUMAnN3 (v3.6) with default options. The functional profiles were further analysed in R. Using the MetaPhlAn4 markers (database vJan21) and StrainPhlAn4 (v4.0.2), we identified sample-specific strains within species with sufficient coverage by setting the StrainPhlAn option MARKER_IN_N_SAMPLES to 40 (marker is found in 40% of the samples) [23,27]. Multiple sequence alignment (MSA) was produced for *Cutibacterium acnes* and *Malassezia restricta* strains implemented by PhyloPhlAn within StrainPhlAn. To inspect whether the MSAs were suitable for phylogenetic reconstruction, likelihood-mapping was done using IQ-TREE 2 (option -LMAP) with 10,000 random quartets [28]. Maximum likelihood phylogenetic tree was constructed from the MSA using IQ-TREE 2 (v2.4.0) with options for ultrafast bootstrap (UFBoot) and the Shimodaira–Hasegawa approximate likelihood ratio test (SH-aLRT) with 1,000 replicates, respectively, to assess branch support [29]. The tree was visualized in R. The R-package CRP-Tree was used for testing whether the strain phylogeny was associated with the allergic sensitization categories [30].

### Contamination monitoring and the use of mock communities

DNA was extracted from six empty samples, serving as negative controls (no starting material added), and two microbial mock community samples to observe kit contamination and extraction and sequencing performance, respectively. The microbial mock community samples included the ZymoBIOMICS Microbial Community Standard II (Zymo Research) and the Skin Microbiome Whole Cell Mix (MSA-2005, ATCC). The negative control and mock community samples were subjected to the same DNA extraction procedure as the skin swab samples, followed by library preparation with seven PCR cycles and shotgun sequencing. Sequencing data from the negative control and mock community samples were quality filtered and taxonomically classified alongside the swab samples using the same tools and parameters (KneadData and MetaPhlAn4, respectively). On average, the number of good-quality microbial reads in the negative control samples was only 0.15% of the number found in the skin swab samples (7–25 thousand reads per sample), indicating minimal contamination in our DNA extraction procedure. Reads in the negative control samples could not be classified using MetaPhlAn4, except for one out of the six samples, in which 22.6% of the reads were identified as *C. acnes*. Decontamination was not carried out on the sequencing data due to minimal contamination in the negative control samples according to MetaPhlAn4 taxonomic classification.

### Alpha and beta diversity and statistical analyses of microbial community structure with associated variables

Alpha diversity in the microbial communities was explored using the Shannon diversity index and Chao1 richness estimator from rarefied count data. Species counts were rarefied ten times, with a different random seed for each iteration, using the ‘rrarefy’ function from the R-package vegan (v2.6-4) [31]. Diversity and richness were calculated for each rarefied count table using the Bioconductor package mia (v1.8.0) [32], and the average per-sample diversity and richness across all iterations was calculated. Beta diversity was analysed by non-metric multidimensional scaling (NMDS) based on the sample-wise Bray–Curtis dissimilarity index on relative abundances of species. The Bray–Curtis dissimilarity index was calculated using the function ‘vegdist’ and the NMDS using the function ‘metaMDS’ with four dimensions within the R-package vegan.

Screening of variables which contributed to the variation in skin microbial compositions was done using the permutational multivariate analysis of variance (PERMANOVA) separately in the Finnish and Russian study populations. Significant variables (*P*<0.05) from PERMANOVA were further modelled using multivariable distance-based redundancy analysis (dbRDA) to determine which variables best explained the inter-individual dissimilarities in the skin microbiomes. The explanatory variables in the final dbRDA models included only those variables that remained significant (*P*<0.05) after adjusting for the effects of age, sex and technical variables. PERMANOVA and dbRDA were performed on the sample-wise Bray–Curtis dissimilarities using the functions ‘adonis2’ and ‘dbrda’, respectively, in the R-package vegan. *P* values were calculated using the functions ‘adonis2’ and ‘anova.cca’, respectively, using 999 permutations on the data.

### Variance partitioning of species abundances and differential abundance analysis

To identify microbial species that were associated with geographical location, hierarchical modelling of species communities (HMSC) was used [33]. The model was run on a server which had the R version 4.2.1 installed. Counts of the microbial species that were present in at least 50% of the samples were included as response variables. The model explanatory variables were sIgE specific to a combination of eight inhalant allergens and timothy grass, birch, mugwort, cat, dog and house dust mite separately, as well as geographical location, library size and fraction of duplicate reads. Library size and fraction of duplicate reads were derived from FastQC analysis. Sex was included in the model as a random variable. Bayesian inference was used to fit species counts to a lognormal Poisson model by using functions ‘Hmsc’ for model construction and ‘sampleMcmc’ for fitting, respectively, within the R-package Hmsc (v3.0.13) [34]. The function ‘sampleMcmc’ was run using options ‘transient=5,000’ and ‘thin=100’. The model was run for 105,000 Markov Chain Monte Carlo iterations. Lastly, variance partitions were created to identify the microbial species for which the geographical location explained most of their variation.

To identify differentially abundant skin microbial species between the Finnish and Russian study populations, as well as between the Finnish NS and HS participants, two methods, LinDA (v0.1.0) and MaAsLin2 (v1.13.0), were used [35,36]. Species with prevalence lower than 10% were excluded from the differential abundance testing, and alpha level *P*<0.001 was used in both methods. Additionally, sex was considered as a random effect within both models. *P* values were adjusted using the Benjamini–Hochberg correction. The compound Poisson linear model was used as the ‘analysis_method’ option in MaAsLin2.

### Network analysis

To understand the interactions within skin microbial communities under the influence of host degree of allergic sensitization, we used network analysis to visualize species co-occurrence relationships in Finnish NS and HS participants. Microbial species co-occurrences were calculated using the Spearman’s correlation coefficient on relative abundance data of species that were present in at least 50% of the Finnish NS and HS samples, respectively. Based on positive correlations of strength >0.6 and false discovery rate (FDR) adjusted *P* value <0.05, weighted undirected networks were constructed using the function ‘graph_from_adjacency_matrix’ in the R-package igraph (v1.4.3) [37]. To compare the network topology and centrality between the NS and HS, networks were constructed using only species that were co-occurring both in NS and HS samples. Furthermore, due to fragmentation in the NS and HS networks, only the network component with the highest number of nodes (i.e. the giant component) was used for the comparison. Clusters of densely connected species within the giant component were identified through random walks using the walktrap method by the ‘cluster_walktrap’ function with default options in the igraph package [37,38]. The giant component closeness and betweenness centrality scores were calculated using the igraph functions ‘closeness’ and ‘betweenness’, respectively [37]. To calculate the degree centrality, the edge weights (the species–species correlations) were considered by calculating the per node sum of weights of all edges per node.

## Results

### Allergic sensitization and lifestyle differences between the Finnish and Russian study populations

Consistent with the original study cohort, allergen-specific sIgE levels were generally higher in the Finnish participants (*n*=60) compared with the Russian participants (*n*=52) (Fig. S1). Notably, birch pollen and timothy grass-specific sIgE levels were higher in the Finnish compared with Russian participants. In contrast, house dust mite-specific sIgE was higher in the Russian participants compared with the Finnish participants (Fig. S1). Of the study population (*n*=112), 11.6% were Russian HS, and 18.8% were Finnish HS, displaying allergen-specific sIgE levels >7.5 kU/L. The portion of both Russian and Finnish participants that were NS or displayed low to intermediate levels of allergen-specific sIgE (SI) was 18.8 and 16.1%, respectively (Fig. S1A). The majority (76.2%) of the Finnish HS participants had experienced symptoms of allergic diseases, including hay fever, asthma and atopic dermatitis, during their lifetime. In the Russian HS participants, a significantly smaller portion (53.8%) had experienced allergy symptoms (Fig. S1D).

The environmental exposures of the Finnish and Russian Karelian study populations were examined through a self-administered questionnaire focused on lifestyles. Farming was more common in Russian Karelia and practised in households located in the city, suburban area and countryside, whereas in Finnish Karelia, farming was practised only in the countryside (Fig. S2). Dog ownership and frequent interaction with dogs within the past 12 months were common in both areas, while cat ownership and frequent interaction with cats were more common in Russian Karelia (Fig. S2).

### Sequencing data, the mock communities and taxonomic profiles of the skin microbiomes

Volar forearm skin microbiomes were explored through metagenomic shotgun sequencing. On average, the fraction of microbial reads was 44% among all paired good-quality reads (3–78 million reads per sample passed quality thresholds, out of which 1.5–39 million reads were of microbial origin). Applying the MetaPhlAn4 database, we identified 2,117 bacterial, 11 eukaryotic and 10 archaeal species-level genome bins, which represent species classification against both reference genomes and metagenome-assembled genomes. On average, 38% of the microbial reads remained unclassified. However, a fraction of the unclassified reads can be expected to be of viral origin. Using the MetaPhlAn3 database allowed us to identify 297 species of DNA viruses.

Consistent with previous human microbiome studies, *bacteria* was the most abundant microbial kingdom in the skin microbiomes (Fig. S3A). The five most abundant microbial phyla were *Actinomycetota*, *Bacillota*, *Pseudomonadota*, *Bacteroidota* (formerly *Actinobacteria*, *Firmicutes*, *Proteobacteria* and *Bacteroidetes*, respectively) and *Basidiomycota* (Fig. S3B). The most abundant genera included *Cutibacterium*, *Corynebacterium*, *Staphylococcus* and *Micrococcus*, as well as *Acinetobacter* (Fig. S3C). The DNA virus composition in the skin was dominated either by bacteriophages, such as *Siphoviridae*, *Myoviridae* and *Podoviridae*, or by *Papillomaviridae* viruses (Fig. S3D)*.* However, it should be noted that these phage taxonomies are outdated [39].

Analysis of the mock community samples revealed differences between the theoretical composition of the mock communities and identified compositions after DNA extraction and sequencing (Fig. S4). Moreover, the least abundant species, contributing to 0.00089% or less in the theoretical relative abundance within the mock community, were not detected. In total, ~7% of the reads remained unclassified in both mock communities. Results from the MetaPhlAn4 taxonomic classification from the microbial mock community samples are explained in more detail in online Supplementary Material.

### Allergen sensitization and environmental exposure were associated with skin microbiomes

**Fig. 2.**
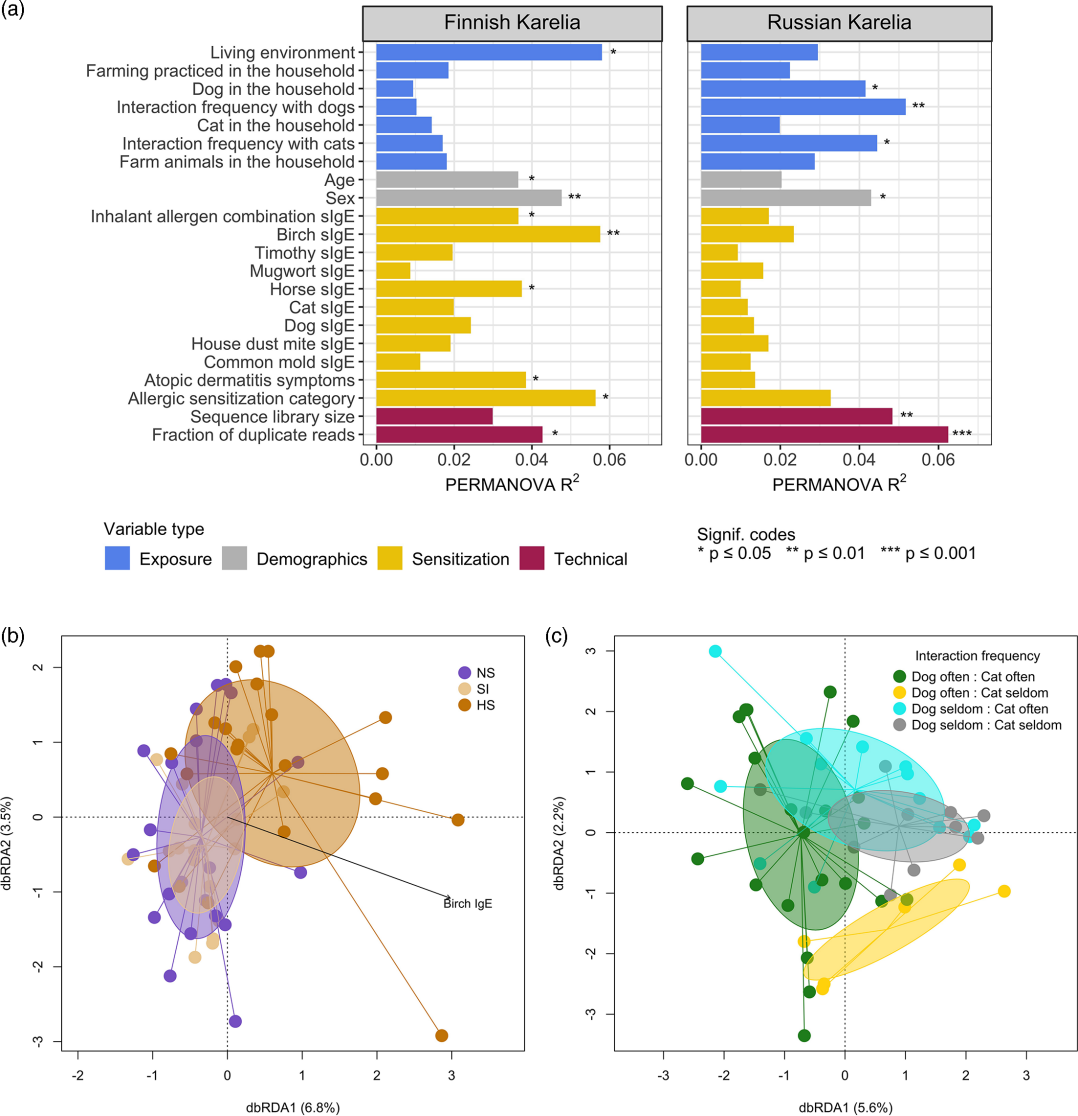
Associations between the skin microbial compositions and factors related to allergen sensitization and environmental exposures. (a) Screening of associations between variation in the microbiome composition and study variables. Effect size (*R*^2^) for factors related to environmental exposures (blue), demographics (grey), allergen sensitization (yellow) and technical factors (purple) from PERMANOVA within the Finnish (left) and Russian (right) study populations. (b) and (c) Modelling of significant variables from (a) using dbRDA on inter-individual dissimilarities in the microbiome, supervised by (b) birch-specific sIgE and allergic sensitization category (NS; SI; HS) within the Finnish study population and (c) interaction frequency with dogs and cats in the past 12 months within the Russian study population, while adjusting for (b, c) sex and fraction of duplicate reads and (b) age and (c) sequence library size. (a–c) Analyses were based on Bray–Curtis dissimilarities, and statistical significance was calculated using 999 permutations of the data.

Compositions of bacteria, eukaryotes and archaea in the Finnish and Russian participants, living in contrasting environments, were associated with distinct factors. In the more urban Finnish Karelia, levels of allergen-specific sIgE, notably birch-specific sIgE, were associated with the skin microbiome composition (Fig. 2a). The association of birch sIgE and allergic sensitization category with skin microbiome remained significant after adjusting for age, sex and fraction of duplicate reads (*P*=0.009 and *P*=0.017, respectively) (Fig. 2b). Furthermore, the microbiome of Finnish HS participants differed from SI and NS participants (FDR-adjusted *P*=0.041, respectively). Additionally, the microbiome functional profiling revealed a distinct composition of functional gene families in the HS participants compared with NS/SI participants in Finnish Karelia (FDR-adjusted *P*=0.04, Fig. S5A). However, only three functional pathways were distinct between NS and HS but marginally significant after *P* value adjusting (Fig. S5B). These pathways included l-histidine degradation and biosynthesis of very long chain fatty acids and UDP-*N*-acetyl-d-glucosamine.

In contrast, in the more rural Russian Karelia, the skin microbiome composition did not differentiate between allergic sensitization categories but was associated with factors related to environmental exposure. This included dog ownership and interaction with dogs and cats within the past 12 months (Fig. 2a). After adjusting for sex, library size and fraction of duplicate reads, interaction frequency with dogs and cats remained significantly associated with the skin microbiome composition (*P*=0.012 and *P*=0.044, respectively) (Fig. 2c). Unlike in Finnish Karelia, the microbiome functional profiles did not differ between allergic sensitization categories in Russian Karelia.

When comparing the skin microbiome between Finnish and Russian participants, both microbial and viral compositions were found to be distinct between the study populations (*P*=0.001 for both, Fig. S6A, B). The diversity and species richness of the skin microbiomes and viral communities were greater in the Russian participants compared with the Finnish (*P*=0.0032 and *P*<0.001, respectively) (Fig. S6C, D). Moreover, the fraction of unclassified reads was greater in the Russians (*P*=0.0052, Fig. S7), suggesting a greater abundance of uncharacterized micro-organisms in Russian Karelia than in Finnish Karelia.

### *C. acnes* and *Malassezia* were significantly abundant in NS participants

**Fig. 3.**
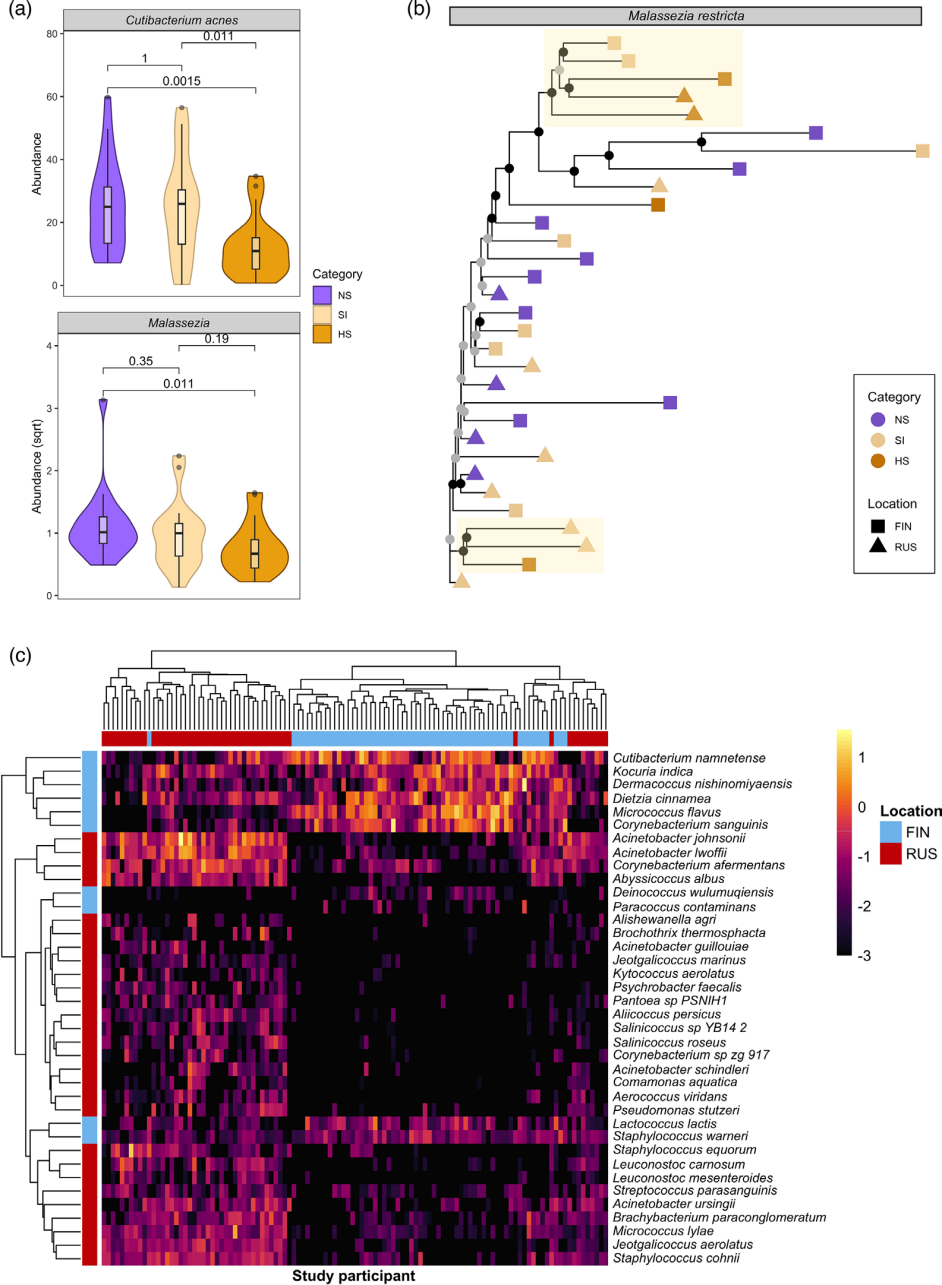
Species associations with allergic sensitization and geographic location in an urban-rural environment. (a) *C. acnes* abundance and square root transformed *Malassezia* spp. abundance in Finnish participants by allergic sensitization category (NS; SI; HS), *P* values calculated using Wilcoxon pairwise comparisons. (b) Maximum likelihood phylogeny of *M. restricta* strains from 31 participant samples built with IQ-TREE 2. The root was set to the midpoint. Nodes are coloured according to UFBoot and SH-aLRT values for branch support calculated within IQ-TREE 2 (black when both values are ≥0.7, otherwise grey). Branch tips are coloured by allergic sensitization category and shaped by the geographical sampling location of the sample from which the strain originated. The light-yellow boxes highlight clades of strains from allergen sensitized participants. The tree topology was associated with the allergic sensitization categories (*P* = 0.044). (c) The relative abundances of the differentially abundant species between the Finnish and Russian study populations in clustered heatmap. Species identified as differentially abundant between Finnish and Russian study populations using both LinDA and MaAsLin2 linear models with adjusted *P* value <0.001 are presented. Relative abundances were transformed with base ten logarithm to improve visualization.

We explored the association between allergic sensitization and species abundances only in the Finnish population, as HS participants did not differ from the NS participants in species composition in the Russian population. Using linear mixed modelling, three abundant species, that is, *C. acnes*, *Malassezia globosa* and *Malassezia sympodialis*, were identified as differentially abundant between Finnish NS and HS participants. The summed abundances of four identified *Malassezia* species (*M. globosa*, *M. restricta*, *M. sympodialis* and *Malassezia pachydermatis*) as well as *C. acnes* abundance were significantly greater in Finnish NS participants compared with HS participants (*P*=0.0015 and *P*=0.0048, respectively) (Fig. 3a).

For strain-level profiling, *C. acnes* and *M. restricta* species markers were present at sufficient coverage in 105 and 31 samples (both Finnish and Russian participants), respectively. According to the likelihood-mapping analysis, *M. restricta* MSA data were suitable for a phylogenetic tree construction, but phylogenetic relationships could not be resolved for *C. acnes* (Fig. S8). Interestingly, IQ-TREE inferred phylogeny of *M. restricta* displayed two clades with strains from Finnish and Russian allergen sensitized participants (Fig. 3b). The phylogenetic tree topology was associated with allergic sensitization categories (*P* = 0.044), but not with geographical sampling location. This may indicate some functional similarities at the genome level within allergic sensitization categories.

### *Acinetobacter johnsonii* and other indicator species were detected for contrasting living environments

Variance partitioning of the Bayesian HMSC model was utilized to evaluate the importance of geographical location among other variables on the variation of the most prevalent species in the skin microbiomes. For several species, the geographical location was the strongest factor explaining the variance in their abundance. For instance, *A. johnsonii* and *Micrococcus flavus* abundances were mostly explained by the geographical location variable (Fig. S9).

As HMSC was applied only to a fraction of the microbial species due to its high computational demands, we utilized linear mixed modelling to further determine species that were differentially abundant between the geographical locations. Altogether, 38 bacterial species were identified as differentially abundant between the Finnish and Russian participants (Fig. 3c). The species associated with the more rural Russian Karelia (*n*=28) included several species of *Acinetobacter*, of which *A. johnsonii* and *Acinetobacter lwoffii* were the most abundant. The most abundant species associated with the more urban Finnish Karelia included *M. flavus* and *Cutibacterium namnetense* (Fig. 3c). The same ten species that had been identified with the Bayesian model were identified with the linear mixed modelling as well. Thus, the results from the different models supported each other.

### Skin microbial species formed a tight network in NS participants

**Fig. 4.**
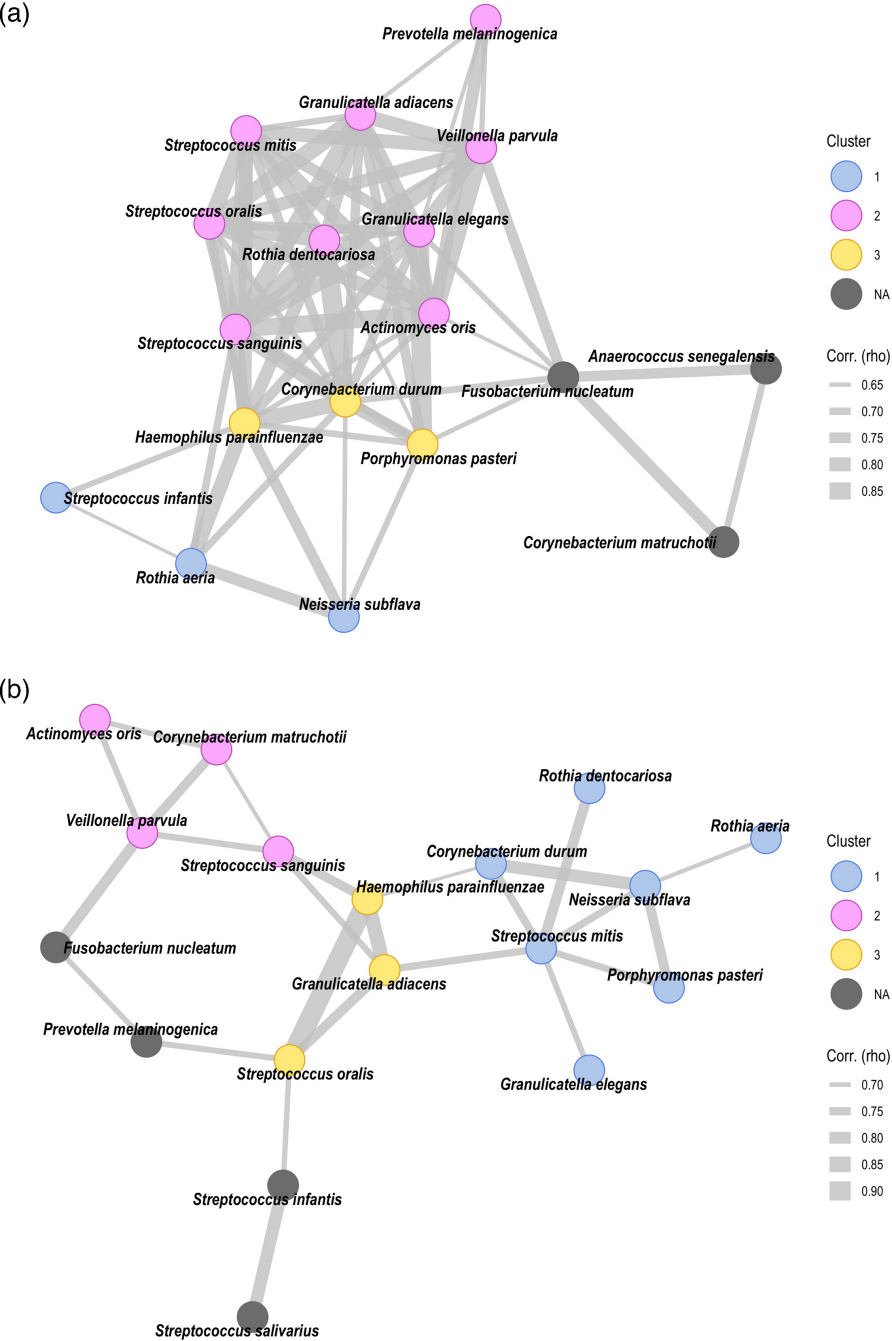
Microbial co-occurrence in the Finnish study population by allergic sensitization category. Microbial co-occurrence network drawn for (a) NS and (b) HS participants for species that correlated strongly (*rho*>0.6, adjusted *P*<0.05). Clusters were identified using the walktrap method.

Microbe–microbe associations of the most prevalent skin microbial species were explored in Finnish NS and HS participants using network analysis. To compare network topology and centrality between the NS and HS, networks were constructed using 18 species showing strong co-occurrence (*rho*>0.6, *P*<0.05) both in NS and HS samples. These 18 species included *Streptococcus*, *Rothia*, *Granulicatella* and *Corynebacterium* species, *Haemophilus parainfluenzae*, *Actinomyces oris*, *Veillonella parvula*, *Prevotella melaninogenica*, *Porphyromonas pasteri*, *Fusobacterium nucleatum* and *Neisseria subflava* (Fig. 4).

The NS network exhibited a greater number of microbial species–species associations compared with the HS network, despite the presence of the same species in both networks (Fig. 4). The NS network had higher weighted degree centrality, representing the number of edges (connections) and summed edge weights (*rho*) per node (species), compared with the HS network (*P*<0.001, Fig. S10). Moreover, the co-occurring species were more closely connected in the NS network according to higher node closeness centrality than in the HS network (*P*<0.001, Fig. S10). Betweenness centrality, indicating the number of times a node falls between shortest paths connecting two other nodes, did not differ between the NS and HS networks (Fig. S10).

## Discussion

The interplay among environmental exposures, the human microbiome and the immune system plays a key role in maintaining health and may also drive disease [2,7]. In urban areas, human exposure to natural environments is reduced, which may lead to deprived human microbiomes and their health-supporting elements [2,4]. The lack of such elements, e.g. specific microbial taxa, functions or metabolites, may in turn result in an inadequately educated immune system and an increased risk of developing inflammatory diseases such as allergies [3,4]. The skin microbiome closely reflects environmental exposures due to its location on the body’s surface, where it continuously interacts with the external environment. We characterized cutaneous bacterial, fungal, archaeal and viral species of allergen-sensitized and NS adolescents living in Finnish and Russian Karelia through WMS sequencing. Finnish and Russian populations within the Karelian region share partly the same ancestry and live in neighbouring, geo-climatically similar regions but present different lifestyle exposures and have sharply contrasting allergy prevalence [8]. The strength of our study lies in providing new species-level information in the context of human skin microbiomes related to environmental exposure and allergic sensitization.

Variation in the skin microbiome composition is associated with many internal and external factors, such as host genetics, age, skin physiology, diet, pet ownership and time spent outdoors [12]. While the skin microbiome in the more urban Finnish Karelia was associated with factors related to allergic sensitization, in the more rural Russian Karelia, the microbiome composition was associated with interaction frequency with pet cats and dogs instead. Dogs and cats may act as transmission vehicles for micro-organisms between the outdoor environment and the owner. However, household pets’ exposure to microbes is limited to the living environment and lifestyle of the owner; thus, the influence of pets may differ between Finnish and Russian Karelia [40]. In Russian Karelia, IgE-mediated allergen sensitization is commonly asymptomatic monosensitization towards the house dust mite (*Dermatophagoides pteronyssinus*) [8]. This may reflect a protective role of monosensitization against the mite, an ectodermal parasite, in the Russian living environment rich with mites in house dust. In Finnish Karelia, house dust was virtually free of mites, but the participants were polysensitized to several allergens (house dust mite among them) and reported clinical allergy symptoms [8,41]. This difference may explain the lack of association between the skin microbiome and allergic sensitization in Russian Karelia, which is apparent in Finnish Karelia.

Finland has urbanized rapidly since World War II, and alongside this urbanization, the prevalence of allergies has increased dramatically [42]. Among Finnish participants, skin microbiome compositions differed between the NS and those with high allergen-specific sIgE levels, in particular associating with birch pollen-specific sIgE levels. Elevated allergen-specific IgE reflects activated B-cell and Th2-type immune signalling, the latter also known to profoundly influence epithelial barrier functions [14,43, 44]. This inflammatory immune milieu with potential negative effects on skin barrier function, in turn, may influence the composition of microbial species colonizing the skin [14,45, 46]. Additionally, the epithelial barrier hypothesis suggests that urban lifestyle results in daily exposure to toxic agents (e.g. particulate matter and detergents) which may damage epithelial barriers, leading to microbes relocating through epithelial barriers and triggering low-grade inflammation [14]. Studies on gut microbiome using a mouse model support the hypothesis that exposure to diverse environmental microbiota reduces the risk of allergic diseases by shaping the microbiome [47,49]. Such studies have shown that a diverse gut microbiome prevents excessive sIgE levels in mice [48,49]. Further investigations are needed to elucidate the mechanisms by which the skin microbiome modulates the immune system to prevent allergic diseases.

Skin microbial and DNA virus species communities differed significantly between the Finnish and Russian study participants. This study did not consider RNA viruses, which limits our estimation of overall skin viral composition and diversity between Finnish and Russian Karelia. The differences in the skin microbiomes may be explained by distinct lifestyle exposures. While Finnish Karelia is westernized, many households in Russian Karelia follow a traditional rural lifestyle, including small-scale farming for their own needs and collecting drinking water from wells or nearby springs [8]. Consistent with previously published results from the original study cohort, Russian Karelia was characterized by a greater abundance of *Acinetobacter* species, such as *A. johnsonii* and *A. lwoffii* [9]. Several *Acinetobacter* species are typical skin commensals but are also abundant in soil and aquatic environments, where they play important roles in the nutrient cycle [50]. *Acinetobacter* abundance on the skin is associated with anti-inflammatory immune responses in healthy individuals, and *in vitro* and mouse studies have shown that *A. lwoffii* suppresses Th2 (allergic) type responses [6,13, 51].

Within the Finnish participants, *C. acnes* and several *Malassezia* species were more abundant in NS participants compared with allergen-sensitized participants. While this study cannot establish a causal link between skin health and *C. acnes* or *Malassezia* spp. abundance, previous studies suggest that these species may help in maintaining healthy skin. *C. acnes* is abundant in adult skin. It supports skin barrier integrity by lowering skin pH through lipid metabolism and by secreting the antioxidant enzyme radical oxygenase (RoxP), which prevents oxidative stress [52,53]. Additionally, *C. acnes* enhances host immune function and barrier defence through the induction of *C. acnes*-specific Th17/Th1 immune responses [54]. Notably, *C. acnes* abundance has been shown to be reduced in atopic dermatitis skin lesions, an inflammatory skin condition characterized by Th2-type immune responses [55]. *Malassezia* spp. are the most common fungi found on human skin [56]. Despite their role in skin disorders, causing irritation following epithelial barrier disruption and sensitization to *Malassezia* antigens, *in vitro* studies have demonstrated anti-inflammatory effects of *Malassezia* as part of immune evasion [56,60]. This immune system modulation is conferred by the lipid-rich capsule surrounding *Malassezia*, masking the pathogen-associated molecular patterns present in the fungi’s cell wall and inducing intracellular IL10 production in host cells [57,58]. *Malassezia* are unable to synthesize lipids; thus, the capsule formation is dependent on host-derived lipids, the abundance of which varies between skin sites and different skin conditions [56,58]. Therefore, individual properties of the skin may play a role in the host–microbe interplay. To elucidate the functions of *C. acnes* and *Malassezia* species in skin health and disease, further genome-level investigations on the interplay between the microbes and the skin in different physiological conditions are needed.

No taxonomic species compositional differences were found in the skin microbiomes between Russian NS and allergen-sensitized participants. The lack of taxonomic differences was possibly due to generally low sIgE levels in Russian Karelia compared to the Finnish participants. This discrepancy limited us to conclude whether the associations between microbial species and allergic sensitization are region-specific to Finnish Karelia or more widespread. However, when inspecting strain-level genomic variation in Finnish and Russian participants together, *M. restricta* strains were often found within the same clade according to allergic sensitization categories despite the geographical sampling location. This finding highlights associations that are not found comparing purely taxonomic species abundances, as within the Russian study population, but indicate that skin microbiome may be involved in allergic sensitization on a strain-specific functional level. In general, our findings suggest that *C. acnes* and *Malassezia* may be involved in the protection against inflammation in healthy skin, but the mechanisms, which may be strain-specific, remain to be elucidated. Additionally, our findings emphasize that the role of the microbiome in allergic sensitization, or protection against it, should be investigated through genomes and microbial functionality. Functional gene families were significantly different between NS and highly allergen-sensitized Finnish participants. Pathways related to bacterial cell wall and surface structures, including very long chain fatty acid and UDP-*N-*acetyl-d-glucosamine biosynthesis, were marginally distinct between NS and highly allergen-sensitized Finnish participants. These functions are involved in bacterial immune evasion mechanisms and virulence and resistance to external stressors [61,63]. Here, more sequencing data would have been needed for an extensive genome-level investigation of microbial strains and strain-level functionality.

Microbial co-occurrence analysis in the Finnish participants demonstrated that strong species–species associations were fewer among participants with high allergen-specific sIgE levels compared with NS participants. High allergen-specific sIgE is a consequence of Th2-polarized immune responses, including the secretion of IL-4 and IL-13 cytokines. These cytokines inhibit skin epithelial barrier component (e.g. filaggrin and loricrin) expression and antimicrobial peptide release onto the skin, thus compromising the skin barrier defence mechanisms [43,64, 65]. We suggest that a disrupted barrier, with the resulting inflammatory responses and dysbiosis, likely impacts microbial species interactions on the skin, driven by the metabolic adaptations necessary for survival in the inflammatory milieu. The effect of host immunology and physiological changes in the skin on microbial species–species interactions remains to be further inspected.

## Conclusions

In this study, we demonstrate differences in the skin microbiomes in NS and allergen-sensitized adolescents in Finnish and Russian Karelia – two areas presenting contrasting lifestyle exposures. Our results expand the previously shown genus-level skin bacterial and fungal microbiota differences between Finnish and Russian Karelia to species- and strain-level differences, including *bacteria*, *archaea*, fungi and DNA viruses. Moreover, we show that the skin microbiome composition is linked with IgE-mediated sensitization that leads to allergy symptoms, as observed in the Finnish study population. Inspection of strain-level variation suggested that studying microbial genomes could provide a more mechanistic view into the crosstalk among the living environment, skin microbiome and allergic sensitization. Our study promotes further research on the role of the human skin microbiome in human health in the context of environmental exposure and the effects of urban lifestyle and decline in exposure to natural environments.

## Supplementary material

10.1099/mgen.0.001527 Uncited Supplementary Material 1.

10.1099/mgen.0.001527 Uncited Supplementary Material 2.

## Abbreviations

dbRDA: distance-based redundancy analysis
FDR: false discovery rate
HMSC: hierarchical modelling of species communities
HS: highly sensitized
ITS: internal transcribed spacer
MSA: multiple sequence alignment
NMDS: non-metric multidimensional scaling
NS: non-sensitized
PERMANOVA: permutational multivariate analysis of variance
SH-aLRT: Shimodaira–Hasegawa approximate likelihood ratio test
SI: sensitized intermediate
sIgE: serum immunoglobulin E
UFBoot: ultrafast bootstrap
WMS: whole metagenome shotgun

## References

[1] Riskumäki M, Ruuskanen MO, Mäenpää K, Ruokolainen L, Mäkelä MJ (2025). Microbiology Society Figshare.

[2] Tong S, Beggs PJ, Davies JM, Jiang F, Kinney PL (2023). Compound impacts of climate change, urbanization and biodiversity loss on allergic disease. Int J Epidemiol.

[3] Haahtela T, Holgate S, Pawankar R, Akdis CA, Benjaponpitak S (2013). The biodiversity hypothesis and allergic disease: world allergy organization position statement. World Allergy Organ J.

[4] von Hertzen L, Hanski I, Haahtela T (2011). Natural immunity. Biodiversity loss and inflammatory diseases are two global megatrends that might be related. EMBO Rep.

[5] Byrd AL, Belkaid Y, Segre JA (2018). The human skin microbiome. Nat Rev Microbiol.

[6] Fyhrquist N, Ruokolainen L, Suomalainen A, Lehtimäki S, Veckman V (2014). *Acinetobacter* species in the skin microbiota protect against allergic sensitization and inflammation. J Allergy Clin Immunol.

[7] Ahn J, Hayes RB (2021). Environmental influences on the human microbiome and implications for noncommunicable disease. Annu Rev Public Health.

[8] Haahtela T, Laatikainen T, Alenius H, Auvinen P, Fyhrquist N (2015). Hunt for the origin of allergy - comparing the Finnish and Russian Karelia. Clin Exp Allergy.

[9] Ruokolainen L, Paalanen L, Karkman A, Laatikainen T, von Hertzen L (2017). Significant disparities in allergy prevalence and microbiota between the young people in Finnish and Russian Karelia. Clin Exp Allergy.

[10] Koskinen J-P, Kiviranta H, Vartiainen E, Jousilahti P, Vlasoff T (2016). Common environmental chemicals do not explain atopy contrast in the Finnish and Russian Karelia. Clin Transl Allergy.

[11] Ruokolainen L, Fyhrquist N, Laatikainen T, Auvinen P, Fortino V (2020). Immune-microbiota interaction in Finnish and Russian Karelia young people with high and low allergy prevalence. Clin Exp Allergy.

[12] Moitinho-Silva L, Boraczynski N, Emmert H, Baurecht H, Szymczak S (2021). Host traits, lifestyle and environment are associated with human skin bacteria. Br J Dermatol.

[13] Hanski I, von Hertzen L, Fyhrquist N, Koskinen K, Torppa K (2012). Environmental biodiversity, human microbiota, and allergy are interrelated. Proc Natl Acad Sci USA.

[14] Akdis CA (2021). Does the epithelial barrier hypothesis explain the increase in allergy, autoimmunity and other chronic conditions?. Nat Rev Immunol.

[15] Ruuskanen MO, Vats D, Potbhare R, RaviKumar A, Munukka E (2022). Towards standardized and reproducible research in skin microbiomes. Environ Microbiol.

[16] Johnson JS, Spakowicz DJ, Hong B-Y, Petersen LM, Demkowicz P (2019). Evaluation of 16S rRNA gene sequencing for species and strain-level microbiome analysis. Nat Commun.

[17] von Hertzen L, Mäkelä MJ, Petäys T, Jousilahti P, Kosunen TU (2006). Growing disparities in atopy between the Finns and the Russians: a comparison of 2 generations. J Allergy Clin Immunol.

[18] Mäenpää K, Wang S, Ilves M, El-Nezami H, Alenius H (2022). Skin microbiota of oxazolone-induced contact hypersensitivity mouse model. PLoS One.

[19] Martin M (2011). Cutadapt removes adapter sequences from high-throughput sequencing reads. EMBnet J.

[20] Bolger AM, Lohse M, Usadel B (2014). Trimmomatic: a flexible trimmer for Illumina sequence data. Bioinformatics.

[21] Langmead B, Salzberg SL (2012). Fast gapped-read alignment with Bowtie 2. Nat Methods.

[22] Andrews S (2010). FastQC: a quality control tool for high throughput sequence data. https://www.bioinformatics.babraham.ac.uk/projects/fastqc.

[23] Blanco-Míguez A, Beghini F, Cumbo F, McIver LJ, Thompson KN (2023). Extending and improving metagenomic taxonomic profiling with uncharacterized species using MetaPhlAn 4. Nat Biotechnol.

[24] Beghini F, McIver LJ, Blanco-Míguez A, Dubois L, Asnicar F (2021). Integrating taxonomic, functional, and strain-level profiling of diverse microbial communities with bioBakery 3. Elife.

[25] Huang R, Soneson C, Ernst FGM, Rue-Albrecht KC, Yu G (2021). TreeSummarizedExperiment: a S4 class for data with hierarchical structure. F1000Res.

[26] Team RC (2023). R: a language and environment for statistical computing.

[27] Truong DT, Tett A, Pasolli E, Huttenhower C, Segata N (2017). Microbial strain-level population structure and genetic diversity from metagenomes. Genome Res.

[28] Strimmer K, von Haeseler A (1997). Likelihood-mapping: a simple method to visualize phylogenetic content of a sequence alignment. Proc Natl Acad Sci USA.

[29] Minh BQ, Schmidt HA, Chernomor O, Schrempf D, Woodhams MD (2020). IQ-TREE 2: new models and efficient methods for phylogenetic inference in the genomic era. Mol Biol Evol.

[30] Zhang J, Rimet F, Bouchez A, Franc A (2024). CRP-Tree: a phylogenetic association test for binary traits. J R Stat Soc C Appl Stat.

[31] Oksanen J (2022).

[32] Ernst FGM (2023).

[33] Ovaskainen O, Tikhonov G, Norberg A, Guillaume Blanchet F, Duan L (2017). How to make more out of community data? A conceptual framework and its implementation as models and software. Ecol Lett.

[34] Tikhonov G (2022).

[35] Zhou H, He K, Chen J, Zhang X (2022). LinDA: linear models for differential abundance analysis of microbiome compositional data. Genome Biol.

[36] Mallick H, Rahnavard A, McIver LJ, Ma S, Zhang Y (2021). Multivariable association discovery in population-scale meta-omics studies. PLoS Comput Biol.

[37] Csardi G, Nepusz T (2006). The igraph software package for complex network research. InterJ.

[38] Pons P, Latapy M (2005). Computing communities in large networks using random walks.

[39] Turner D, Kropinski AM, Adriaenssens EM (2021). A roadmap for genome-based phage taxonomy. Viruses.

[40] Lehtimäki J, Sinkko H, Hielm-Björkman A, Laatikainen T, Ruokolainen L (2020). Simultaneous allergic traits in dogs and their owners are associated with living environment, lifestyle and microbial exposures. Sci Rep.

[41] Von Hertzen LC, Laatikainen T, Pennanen S, Mäkelä MJ, Haahtela T (2008). ALLERGY Net: is house dust mite monosensitization associated with clinical disease?. Allergy.

[42] Laatikainen T, von Hertzen L, Koskinen J-P, Mäkelä MJ, Jousilahti P (2011). Allergy gap between Finnish and Russian Karelia on increase. Allergy.

[43] Walker JA, McKenzie ANJ (2018). TH2 cell development and function. Nat Rev Immunol.

[44] van Ree R, Hummelshøj L, Plantinga M, Poulsen LK, Swindle E (2014). Allergic sensitization: host-immune factors. Clin Transl Allergy.

[45] Belkaid Y, Segre JA (2014). Dialogue between skin microbiota and immunity. Science.

[46] Riskumäki M, Tessas I, Ottman N, Suomalainen A, Werner P (2021). Interplay between skin microbiota and immunity in atopic individuals. Allergy.

[47] Yang Z, Chen Z, Lin X, Yao S, Xian M (2022). Rural environment reduces allergic inflammation by modulating the gut microbiota. Gut Microbes.

[48] Cahenzli J, Köller Y, Wyss M, Geuking MB, McCoy KD (2013). Intestinal microbial diversity during early-life colonization shapes long-term IgE levels. Cell Host Microbe.

[49] Wyss M, Brown K, Thomson CA, Koegler M, Terra F (2019). Using precisely defined *in vivo* microbiotas to understand microbial regulation of IgE. Front Immunol.

[50] Jung J, Park W (2015). Acinetobacter species as model microorganisms in environmental microbiology: current state and perspectives. Appl Microbiol Biotechnol.

[51] Debarry J, Garn H, Hanuszkiewicz A, Dickgreber N, Blümer N (2007). *Acinetobacter lwoffii* and *Lactococcus lactis* strains isolated from farm cowsheds possess strong allergy-protective properties. J Allergy Clin Immunol.

[52] Allhorn M, Arve S, Brüggemann H, Lood R (2016). A novel enzyme with antioxidant capacity produced by the ubiquitous skin colonizer *Propionibacterium acnes*. Sci Rep.

[53] Rozas M, Hart de Ruijter A, Fabrega MJ, Zorgani A, Guell M (2021). From dysbiosis to healthy skin: major contributions of *Cutibacterium acnes* to skin homeostasis. Microorganisms.

[54] Kistowska M, Meier B, Proust T, Feldmeyer L, Cozzio A (2015). *Propionibacterium acnes* promotes Th17 and Th17/Th1 responses in acne patients. J Invest Dermatol.

[55] Fyhrquist N, Muirhead G, Prast-Nielsen S, Jeanmougin M, Olah P (2019). Microbe-host interplay in atopic dermatitis and psoriasis. Nat Commun.

[56] Grice EA, Dawson TLJ (2017). Host-microbe interactions: *Malassezia* and human skin. Curr Opin Microbiol.

[57] Thomas DS, Ingham E, Bojar RA, Holland KT (2008). *In vitro* modulation of human keratinocyte pro- and anti-inflammatory cytokine production by the capsule of *Malassezia* species. FEMS Immunol Med Microbiol.

[58] Kesavan S, Holland KT, Ingham E (2000). The effects of lipid extraction on the immunomodulatory activity of *Malassezia* species *in vitro*. Med Mycol.

[59] Glatz M, Bosshard PP, Hoetzenecker W, Schmid-Grendelmeier P (2015). The role of *Malassezia* spp. in atopic dermatitis. J Clin Med.

[60] Sparber F, De Gregorio C, Steckholzer S, Ferreira FM, Dolowschiak T (2019). The skin commensal yeast *Malassezia* triggers a type 17 response that coordinates anti-fungal immunity and exacerbates skin inflammation. Cell Host Microbe.

[61] Kneidinger B, O’Riordan K, Li J, Brisson J-R, Lee JC (2003). Three highly conserved proteins catalyze the conversion of UDP-N-acetyl-D-glucosamine to precursors for the biosynthesis of O antigen in *Pseudomonas aeruginosa* O11 and capsule in *Staphylococcus aureus* type 5. Implications for the UDP-N-acetyl-L-fucosamine biosynthetic pathway. J Biol Chem.

[62] Komaniecka I, Żebracki K, Mazur A, Suśniak K, Sroka-Bartnicka A (2025). The absence of a very long chain fatty acid (VLCFA) in lipid A impairs *Agrobacterium fabrum* plant infection and biofilm formation and increases susceptibility to environmental stressors. Molecules.

[63] Bourassa DV, Kannenberg EL, Sherrier DJ, Buhr RJ, Carlson RW (2017). The lipopolysaccharide lipid A long-chain fatty acid is important for *Rhizobium leguminosarum* growth and stress adaptation in free-living and nodule environments. Mol Plant Microbe Interact.

[64] Furue M (2020). Regulation of filaggrin, loricrin, and involucrin by IL-4, IL-13, IL-17A, IL-22, AHR, and NRF2: pathogenic implications in atopic dermatitis. Int J Mol Sci.

[65] Hönzke S, Wallmeyer L, Ostrowski A, Radbruch M, Mundhenk L (2016). Influence of Th2 cytokines on the cornified envelope, tight junction proteins, and ß-defensins in filaggrin-deficient skin equivalents. J Invest Dermatol.

