# Supplementary material for: Shotgun metagenomics reveals distinct skin microbial species in allergen-sensitized individuals
Source: Microb Genom. 2025 Dec 3;11(12):001527. doi: 10.1099/mgen.0.001527 (PMC13293315; doi:10.1099/mgen.0.001527)
Supplement: Uncited Supplementary Material 1. [file mgen-11-01527-s001.pdf]

R script for the manuscript 'Shotgun metagenomics reveals distinct skin microbial species in allergen sensitized individuals'

Matilda Riskumäki

```
knitr::opts_chunk$set(dev='cairo_pdf')

library(dplyr)
library(tidyr)
library(tibble)
library(vegan)
library(TreeSummarizedExperiment)
library(mia)
library(miaViz)
library(ecodist)
library(scales)
library(patchwork)
library(LinDA)
library(Maaslin2)
library(reshape2)
library(ggplot2)
library(ggpubr)
library(ggvenn)
library(RColorBrewer)
library(GGally)
library(pheatmap)
library(treeio)
library(ggtree)
library(tidytree)
library(paleotree)
library(phylobase)
library(phylosignal)
library(ggnewscale)
library(Hmisc)
library(here)
library(igraph)
library(tidyverse)
library(tidygraph)
library(ggraph)
library(BiodiversityR)
```

1) Data

Taxonomic profiles were recovered from quality trimmed and filtered reads using the MetaPhlAn (v4.0.3, database vJan21 for prokaryotic and eukaryotic reads and v3.1.0 for viral reads). Species relative abundances and counts were used in downstream analyses. MetaPhlAn4 (prokaryotes and eukaryotes) output was filtered to include only species that were identified down to species-level genome bins (SGB), while MetaPhlAn3

(viruses) output was filtered to include only species-level identifications.

```r
# MetaPhlAn4 output----

# MetaPhlAn 4 output----

# relative abundance
taxonomy_relab <- read.table("Additional_file_2_mpa4.0.3_abundance_table.txt",
                        sep = "\t", row.names = 1, header = T, check.names = F)

# counts
taxonomy_count <- read.table("Additional_file_3_mpa4.0.3_count_table.txt",
                              sep = "\t", row.names = 1, header = T, check.names = F)

# taxonomy table
taxa_df <- data.frame(Taxa = row.names(taxonomy_relab)) %>%
  separate(Taxa, into = c("Kingdom", "Phylum", "Class", "Order", "Family", "Genus", "Species", "SGB"),
           sep = ";[a-z]__")

# set SGB codes as rownames
rownames(taxa_df) <- taxa_df$SGB

# modify row names in the abundance and count tables to match the row names in taxa_df
rownames(taxonomy_relab) <- gsub(".*t__", "", rownames(taxonomy_relab))
rownames(taxonomy_count) <- gsub(".*t__", "", rownames(taxonomy_count))

# MetaPhlAn 3 output----

# relative abundance
virus_relab <- read.table("Additional_file_4_mpa3_virus_abundance_table.txt",
                          sep = "\t", row.names = 1, header = T, check.names = F)

# counts
virus_count <- read.table("Additional_file_5_mpa3_virus_count_table.txt",
                          sep = "\t", row.names = 1, header = T, check.names = F)

# the samples are in reverse order in the virus abundance and count tables
# --> fix this to make the tables compatible with the sample order in metadata table

virus_relab <- virus_relab[,rev(names(virus_relab))]
virus_count <- virus_count[,rev(names(virus_count))]

# taxonomy table
virus_taxa_df <- data.frame(Taxa = row.names(virus_relab)) %>%
  separate(Taxa, into = c("Kingdom", "Phylum", "Class", "Order", "Family", "Genus", "Species"),
           sep = ";[a-z]__")

# set rownames to the taxonomy table
rownames(virus_taxa_df) <- paste0("V", seq(nrow(virus_taxa_df)))

# set rownames to abundance and count tables to match those with the taxonomy table
rownames(virus_relab) <- paste0("V", seq(nrow(virus_relab)))
rownames(virus_count) <- paste0("V", seq(nrow(virus_count)))
```

The metadata table includes clinical data, including allergen specific serum IgE measurements, from the

participants, as well as survey questionnaire -based data about health and lifestyles. Serum IgE specific for a combination of eight inhalant allergens (column 'AllMix' in metadata table) was measured from all participants. The allergens included birch, timothy, mugwort, dog, cat, horse, *Dermatophagoides pteronyssinus*, and *Cladosporium herbarum*. Serum IgE specific for each single allergen listed above was measured separately from participants whose serum IgE against the combination was higher than 0.35 kU/L. Altogether 42/112 participants had 'AllMix' < 0.35 kU/L. For these participants the value for the separately measured allergens was set to 0 for further analyses. DNA sample and sequencing derived technical information, such as DNA concentration and sequencing library size, respectively, is also included in the metadata table. Per sample fraction of unclassified reads from the MetaPhlAn4 output is also in the metadata table.

```
# load metadata table
metadata_w_tech <- read.table("Additional_file_6_metadata_w_tech.txt", sep = "\t", stringsAsFactors = T)
str(metadata_w_tech)
```

```
## 'data.frame':    112 obs. of  35 variables:
##  $ Location          : Factor w/ 2 levels "FIN","RUS": 2 2 2 2 2 2 2 2 2 2 ...
##  $ Age               : int  19 18 17 18 17 18 16 17 16 16 ...
##  $ Sex               : Factor w/ 2 levels "F","M": 2 2 1 2 2 1 2 1 2 2 ...
##  $ Timothy           : num  0 0 0.02 0.02 0 0 0 0.07 19.1 0.02 ...
##  $ Birch             : num  0 0 0.08 0.01 0 0 0 0.07 0.03 0.01 ...
##  $ Mugwort           : num  0 0 0.09 0.01 0 0 0 1 0.02 0.01 ...
##  $ Horse             : num  0 0 0.09 0.02 0 0 0 0.03 0.02 0.04 ...
##  $ Cat               : num  0 0 0.37 0.02 0 0 0 0.05 0.06 0.05 ...
##  $ Dog               : num  0 0 0.21 0.02 0 0 0 0.05 0.04 0.05 ...
##  $ Dptero            : num  0 0 50.4 0.05 0 0 0 2.93 0.05 14.7 ...
##  $ Cladherb          : num  0 0 0.02 0.01 0 0 0 0.02 0.02 0.02 ...
##  $ AllMix            : num  0.04 0.07 49.6 1.44 0.05 0.07 0.04 3.96 14.6 13.6 ...
##  $ Category          : Factor w/ 3 levels "HS","NS","SI": 2 2 1 3 2 2 2 3 1 1 ...
##  $ Asthma            : Factor w/ 2 levels "healthy","symptoms": 1 1 1 1 1 1 1 1 1 2 ...
##  $ Hay_fever         : Factor w/ 2 levels "healthy","symptoms": 2 1 1 1 1 1 1 1 1 2 ...
##  $ Eczema_12mo       : Factor w/ 2 levels "no","yes": 2 1 1 1 2 1 1 1 1 1 ...
##  $ Atopic_derm       : Factor w/ 2 levels "healthy","symptoms": 1 1 2 1 1 2 1 1 1 2 ...
##  $ Farming           : Factor w/ 2 levels "no","yes": 2 2 2 1 1 1 2 1 1 1 ...
##  $ Dog_owner         : Factor w/ 2 levels "no","yes": 2 2 2 2 1 2 2 1 2 2 ...
##  $ Cat_owner         : Factor w/ 2 levels "no","yes": 2 2 1 2 2 2 2 2 2 2 ...
##  $ Farm_animals      : Factor w/ 2 levels "no","yes": 2 1 1 2 1 1 1 1 1 1 ...
##  $ Dog_12mo          : Factor w/ 2 levels "often","seldom": 1 1 1 1 2 1 1 2 1 1 ...
##  $ Cat_12mo          : Factor w/ 2 levels "often","seldom": 1 1 1 1 1 1 1 1 1 1 ...
##  $ Farm_animals_12mo: Factor w/ 2 levels "often","seldom": 1 2 2 2 2 2 2 2 2 2 ...
##  $ Farm_milk         : Factor w/ 2 levels "often","seldom": 1 2 2 1 2 2 1 2 2 2 ...
##  $ Smoking_house     : Factor w/ 2 levels "no","yes": 2 2 2 2 2 2 1 2 2 1 ...
##  $ Allerg_dis        : Factor w/ 2 levels "healthy","symptoms": 2 1 2 1 1 2 1 1 1 2 ...
##  $ DNA_conc          : num  0.092 0.173 0.107 0.253 0.01 0.096 0.144 0.069 0.194 0.074 ...
##  $ DNAexc_kit        : int  2 2 2 2 2 2 2 3 2 2 ...
##  $ Lib_plate         : Factor w/ 3 levels "Plate_1","Plate_2",..: 2 1 2 1 3 2 2 2 1 2 ...
##  $ M_total_seqs      : num  11.5 7.8 7 13.3 8.2 12.1 14.4 6.8 39 4.9 ...
##  $ Dups_prc          : num  22.6 18.8 21.4 17.8 31.7 15 20.2 18.9 26.2 18.6 ...
##  $ loc.categ         : Factor w/ 6 levels "Fin.HS","Fin.NS",..: 5 5 4 6 5 5 5 6 4 4 ...
##  $ Living_env        : Factor w/ 3 levels "city_center",..: 1 1 2 3 1 1 2 1 2 1 ...
##  $ Unclassified      : num  34.6 26.1 49.5 65 30.5 ...
```

The data is stored into a TreeSummarizedExperiment object using the Bioconductor package TreeSummarizedExperiment (v2.8.0) for further analyses. The relative abundance and count tables include taxonomic profiles from both swab samples and positive control samples. The positive control samples and the species present exclusively in those samples must be excluded from the TreeSummarizedExperiment object. Positive

control samples are present only in the MetaPhlAn 4 output.

```
# remove the positive control samples (the last six rows) from the abundance and count tables
swab_relab <- taxonomy_relab[,-c(113:118)]
swab_count <- taxonomy_count[,-c(113:118)]

# Remove taxa exclusively present in the positive controls
swab_relab <- swab_relab[rowSums(swab_relab[])>0,]
swab_count <- swab_count[rowSums(swab_count[])>0,]

# contamination was identified in the positive control samples (species Dechlorosoma suillum on row 154
# remove the contaminating species
swab_relab <- swab_relab[-154,]
swab_count <- swab_count[-154,]

# ...from the taxonomy table too
swab_taxa <- taxa_df[rownames(swab_relab),]

# TreeSummarizedExperiment (TSE) object
# Prokaryotic and eukaryotic taxonomies----

tse <- TreeSummarizedExperiment(assays = list(Abundance = as.matrix(swab_relab),
                                              Count = as.matrix(swab_count)),
                                rowData = swab_taxa,
                                colData = metadata_w_tech)

# create TSE object for FIN

tse_FIN <- tse[ ,tse$Location == "FIN"]

sum(rowSums(assay(tse_FIN, "Abundance")) == 0)
# 743 taxa are exclusively in RUS samples
# remove these from FIN TSE object

tse_FIN <- tse_FIN[rowSums(assay(tse_FIN, "Abundance")) > 0, ]

# create TSE object for RUS

tse_RUS <- tse[ ,tse$Location == "RUS"]

sum(rowSums(assay(tse_RUS, "Abundance")) == 0)
# 384 taxa are exclusively in FIN samples
# remove these from RUS TSE object

tse_RUS <- tse_RUS[rowSums(assay(tse_RUS, "Abundance")) > 0, ]

# TSE object for viruses-----

tse_vir <- TreeSummarizedExperiment(assays = list(Abundance = as.matrix(virus_relab),
                                                  Count = as.matrix(virus_count)),
                                    rowData = virus_taxa_df,
                                    colData = metadata_w_tech)
```

2) Alpha diversity

Alpha diversity and estimated species richness were calculated using the Bioconductor package mia (v1.8.0). The diversity and richness were calculated on rarefied count data. The data was rarefied using the R-package vegan (v2.6-4). The count data was rarefied with 10x permutation and the Shannon diversity index and Chao1 richness estimation were calculated for each iteration of rarefied counts. The average Shannon diversity and Chao1 richness were used for comparison between the Finnish and Russian study populations.

```r
# function for violin plots
plotByLocation <- function(data, x, y){
  ggplot(as.data.frame({{data}}), aes({{x}},{{y}})) +
    geom_violin(aes(fill={{x}}, col = {{x}})) +
    geom_boxplot(width=0.1, alpha = 0.5) +
    scale_fill_manual(values=c("lightblue1", "indianred1")) +
    scale_color_manual(values = c("skyblue3", "red3")) +
    stat_compare_means(method = "wilcox.test", label = "p.format", label.x = 1.4) +
    theme_classic() +
    theme(plot.title = element_text(size = 20, face = "bold", hjust = 0.2),
          axis.title.x = element_blank(), legend.position = "none")
}

# rarefied counts----
# Prokaryota and Eukaryota
rarefied_m <- list(NULL)

for (i in 1:10) {
  set.seed(64*i)
  rarefied <- rrarefy(t(assay(tse, "Count")),
                      sample = min(colSums(assay(tse, "Count"))))
  rarefied_m[[i]] <- t(rarefied)
}

# Viruses
rarefied_v <- list(NULL)

for (i in 1:10) {
  set.seed(59*i)
  rarefied <- rrarefy(t(assay(tse_vir, "Count")),
                      sample = min(colSums(assay(tse_vir, "Count"))))
  rarefied_v[[i]] <- t(rarefied)
}

# Shannon diversity----
# Prokaryota and Eukaryota
diversity_m <- list(NULL)

for (i in 1:length(rarefied_m)) {
  tse_rarefied <- TreeSummarizedExperiment(
    assays = list(Count = rarefied_m[[i]]),
    rowData = rowData(tse),
    colData = colData(tse)
  )
  tse_rarefied <- estimateDiversity(tse_rarefied,
                                    assay.type = "Count",
                                    index = "shannon",
```

```r
                               name = "Shannon")
  diversity <- colData(tse_rarefied)$Shannon
  diversity_m[[i]] <- diversity
}

diversity_m <- do.call(cbind, diversity_m)
diversity_mavg <- rowMeans(diversity_m)

colData(tse)$Shannon <- as.numeric(diversity_mavg)

# Viruses
diversity_v <- list(NULL)

for (i in 1:length(rarefied_v)) {
  tse_rarefied <- TreeSummarizedExperiment(
    assays = list(Count = rarefied_v[[i]]),
    rowData = rowData(tse_vir),
    colData = colData(tse_vir)
  )
  tse_rarefied <- estimateDiversity(tse_rarefied,
                               assay.type = "Count",
                               index = "shannon",
                               name = "Shannon")
  diversity <- colData(tse_rarefied)$Shannon
  diversity_v[[i]] <- diversity
}

diversity_v <- do.call(cbind, diversity_v)
diversity_vavg <- rowMeans(diversity_v)

colData(tse_vir)$Shannon <- as.numeric(diversity_vavg)

# plot
ggarrange(plotByLocation(colData(tse), Location, Shannon),
plotByLocation(colData(tse_vir), Location, Shannon),
labels = c("Prok. + Euk.", "Viruses"),
label.y = 1.015, label.x = c(0.03, 0.08))
```

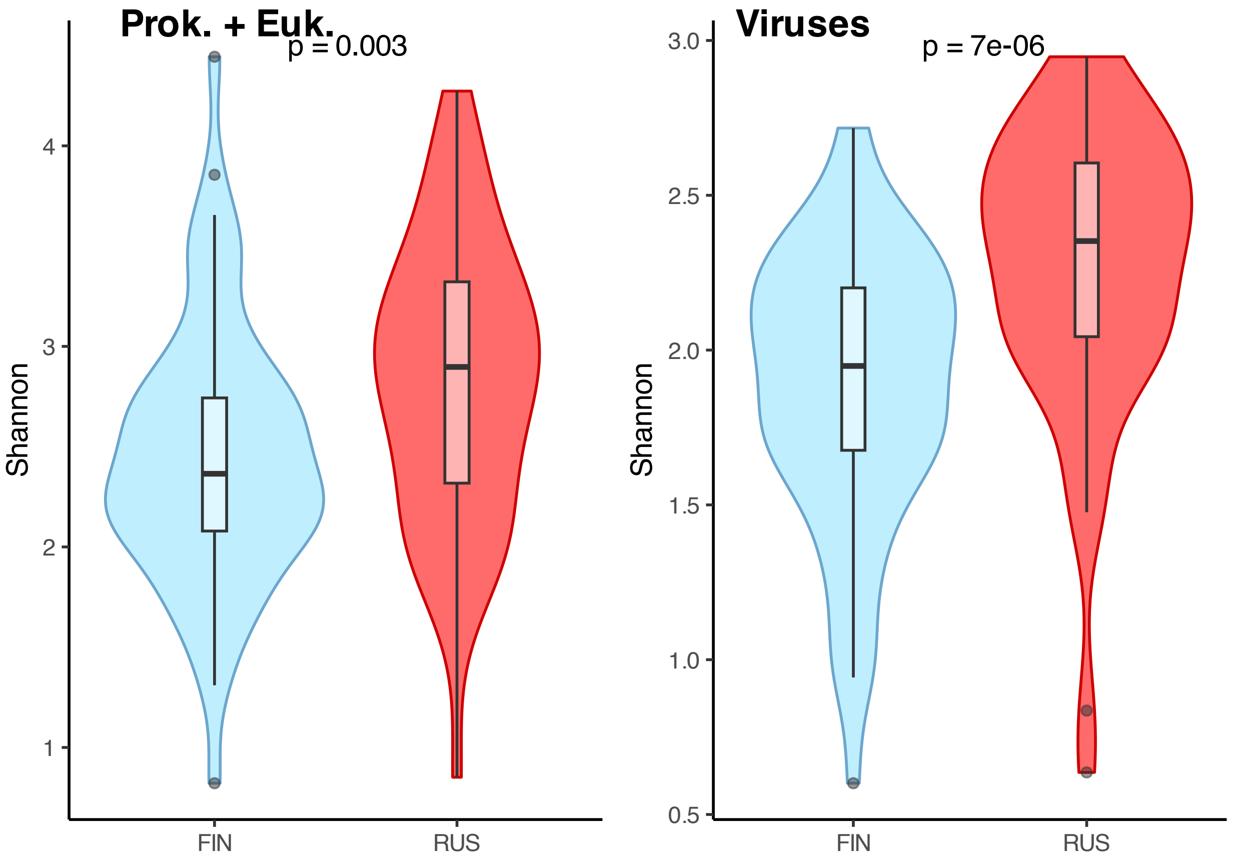


```
# Estimated species richness (Chao1)----
# Prokaryota and Eykaryota
richness_m <- list(NULL)

for (i in 1:length(rarefied_m)) {
  tse_rarefied <- TreeSummarizedExperiment(
    assays = list(Count = rarefied_m[[i]]),
    rowData = rowData(tse),
    colData = colData(tse)
  )
  tse_rarefied <- estimateRichness(tse_rarefied,
                                   assay.type = "Count",
                                   index = "chao1",
                                   name = "Chao1")
  richness <- colData(tse_rarefied)$Chao1
  richness_m[[i]] <- richness
}

richness_m <- do.call(cbind, richness_m)
richness_mavg <- rowMeans(richness_m)

colData(tse)$Chao1 <- as.numeric(richness_mavg)

# Viruses
richness_v <- list(NULL)

for (i in 1:length(rarefied_v)) {
```

```
  tse_rarefied <- TreeSummarizedExperiment(
    assays = list(Count = rarefied_v[[i]]),
    rowData = rowData(tse_vir),
    colData = colData(tse_vir)
  )
  tse_rarefied <- estimateRichness(tse_rarefied,
                                   assay.type = "Count",
                                   index = "chao1",
                                   name = "Chao1")
  richness <- colData(tse_rarefied)$Chao1
  richness_v[[i]] <- richness
}

richness_v <- do.call(cbind, richness_v)
richness_vavg <- rowMeans(richness_v)

colData(tse_vir)$Chao1 <- as.numeric(richness_vavg)

# plot
ggarrange(plotByLocation(colData(tse), Location, Chao1),
plotByLocation(colData(tse_vir), Location, Chao1),
labels = c("Prok. + Euk.", "Viruses"),
label.y = 1.015, label.x = c(0.03, 0.08))
```

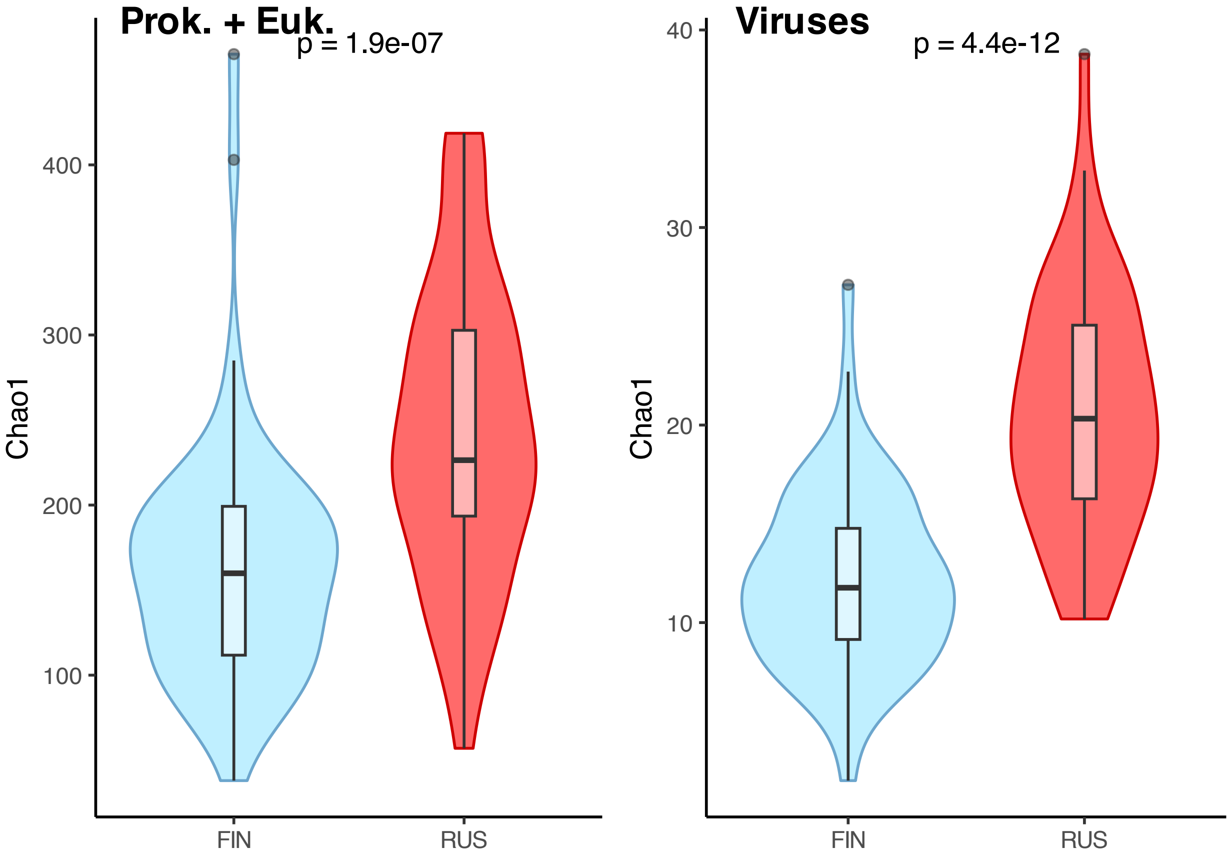

3) Beta diversity

Beta diversity was analyzed by non-metric multidimensional scaling (NMDS), based on the sample-wise Bray-Curtis dissimilarity index on the relative abundance data. The Bray-Curtis dissimilarity index was calculated and the NMDS using the R-package vegan (v2.6-4).

```
# beta diversity

bray_curtis <- vegdist(t(assay(tse, "Abundance")), method = "bray")

# variation
boxplot(betadisper(bray_curtis, metadata_w_tech$Location))
```

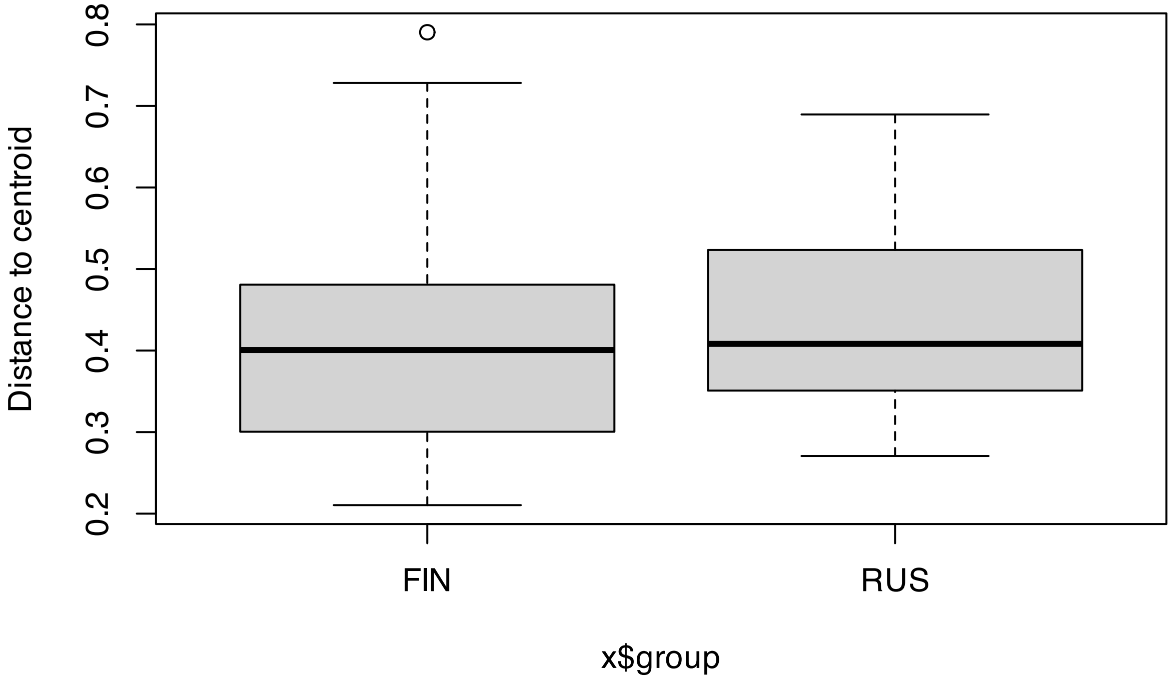


```
# viruses
bray_curtis_virus <- vegdist(t(assay(tse_vir, "Abundance")), method = "bray")
boxplot(betadisper(bray_curtis_virus, metadata_w_tech$Location))
```

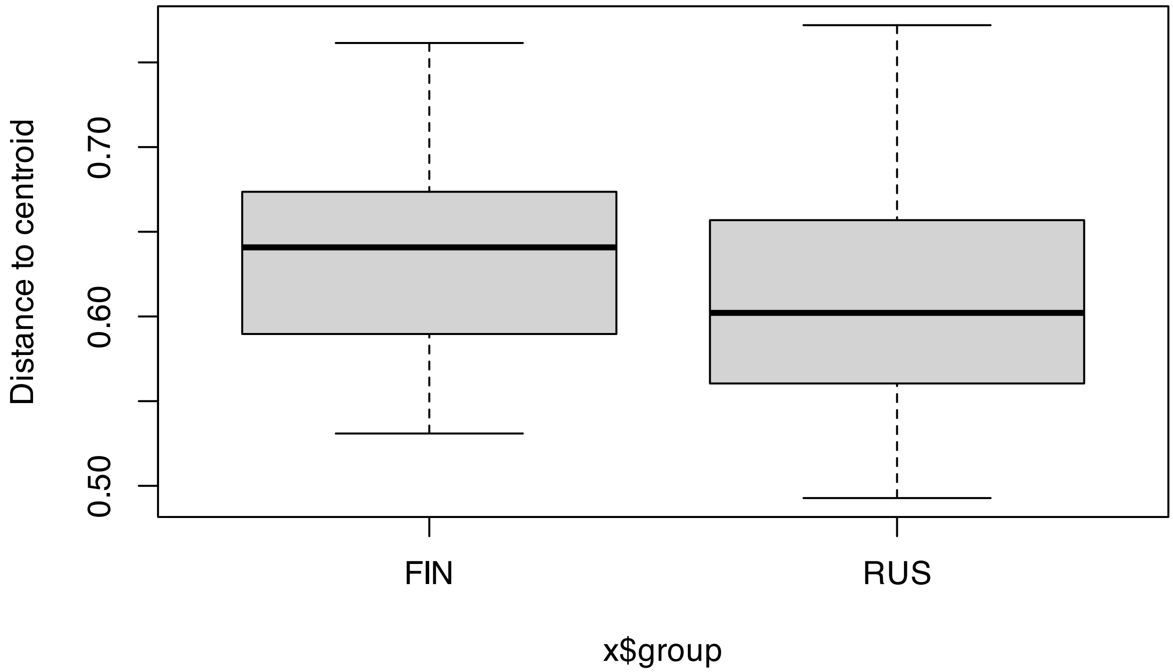


```r
# NMDS prokaryotes + eukaryotes----

set.seed(891)
bc_nmds <- metaMDS(t(assay(tse, "Abundance")),
                   distance = "bray", k = 4)

bc_nmds # stress: ~0.13
stressplot(bc_nmds)
```

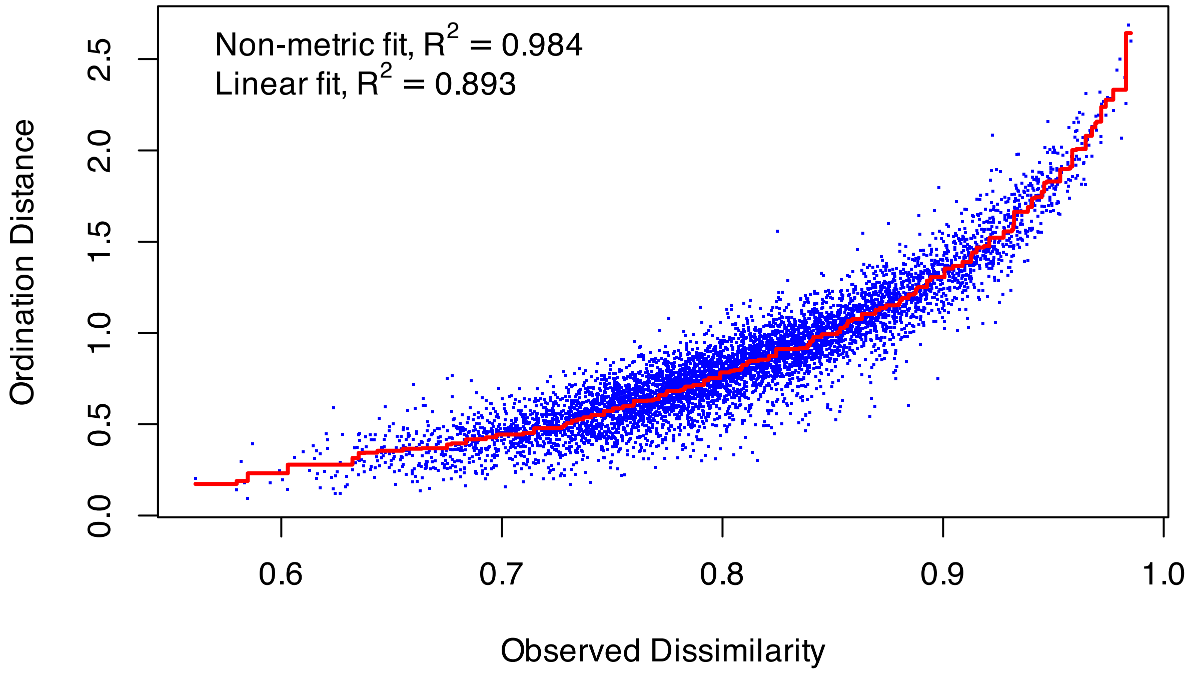


```r
# plot
col_location = c("skyblue2", "red3")[metadata_w_tech$Location]
```

```
plot(bc_nmds$points)
with(metadata_w_tech,
     points(bc_nmds$points, display = "sites",
            col = col_location,
            pch = 19,
            cex = 2))
ordispider(bc_nmds, metadata_w_tech$Location, col = c("skyblue2", "red3"))
ordiellipse(bc_nmds, metadata_w_tech$Location,
            col = c("skyblue2", "red3"), border = c("skyblue2", "red3"), draw = "polygon", label = F)
legend("topleft", inset = 0.05, legend = c("FIN","RUS"), pch = 19, pt.cex = 2,
       col = c("skyblue2", "red3"), box.lty = 0)
```

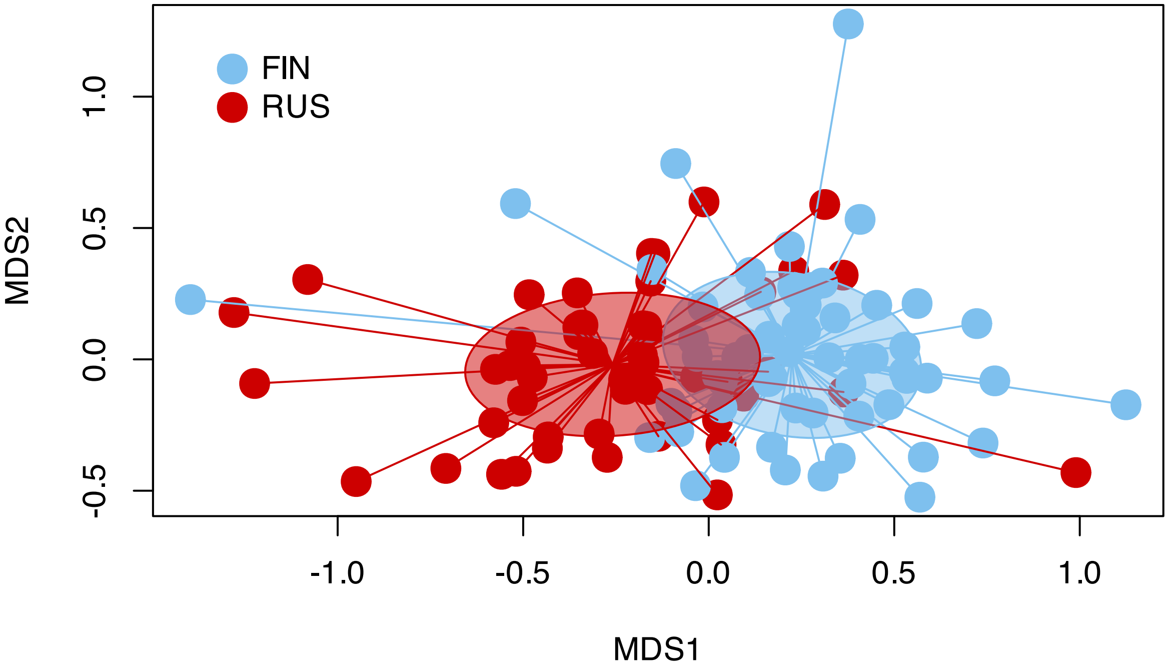


```
# NMDS viruses----

# number of viral species present in each sample
rowSums(t(assay(tse_vir, "Abundance")) != 0)
# sample 3536 has only two viral species present --> omit the sample from NMDS

set.seed(932)
virus_bc_nmds <- metaMDS(t(assay(tse_vir, "Abundance")[,-76]),
                         distance = "bray", k = 4)
# omit sample 3536 (row 76)

virus_bc_nmds # stress: ~0.2
stressplot(virus_bc_nmds)
```

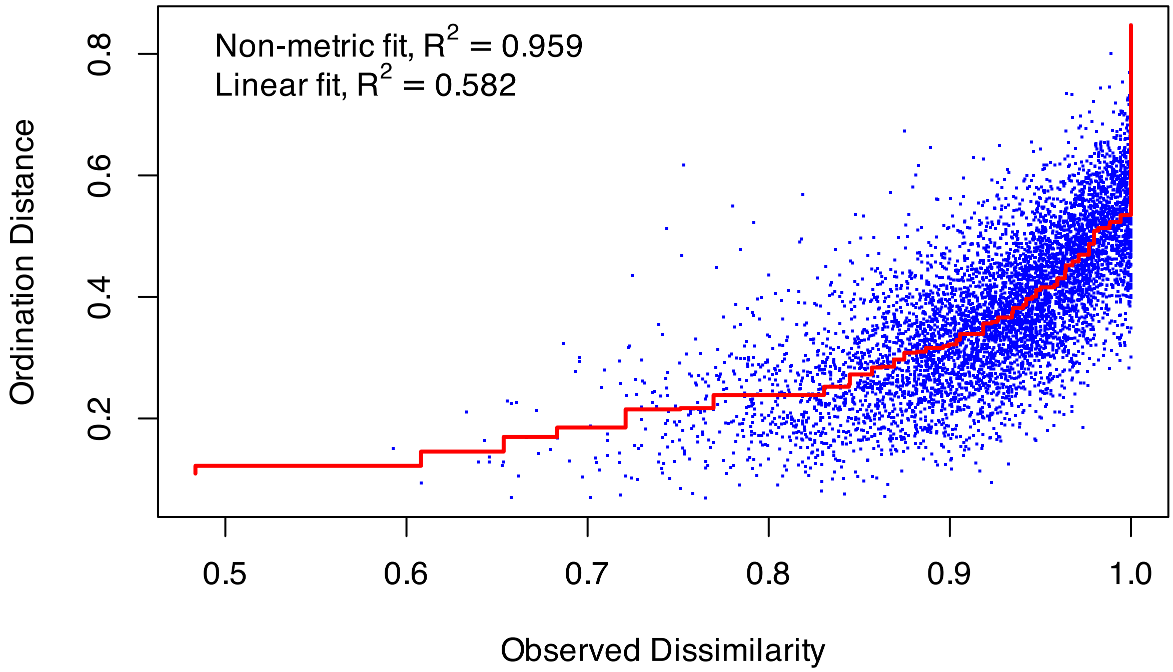


```
# plot
plot(virus_bc_nmds$points)
with(metadata_w_tech,
     points(virus_bc_nmds$points, display = "sites",
            col = col_location,
            pch = 19,
            cex = 2))
ordispider(virus_bc_nmds, metadata_w_tech[-76,1], col = c("skyblue2", "red3"))
ordiellipse(virus_bc_nmds, display = "sites", metadata_w_tech[-76,1],
            col = c("skyblue2", "red3"), border = c("skyblue2", "red3"), draw = "polygon", label = F)
legend("bottomleft", inset = 0.05, legend = c("FIN","RUS"), pch = 19, pt.cex = 2,
       col = c("skyblue2","red3"), box.lty = 0)
```

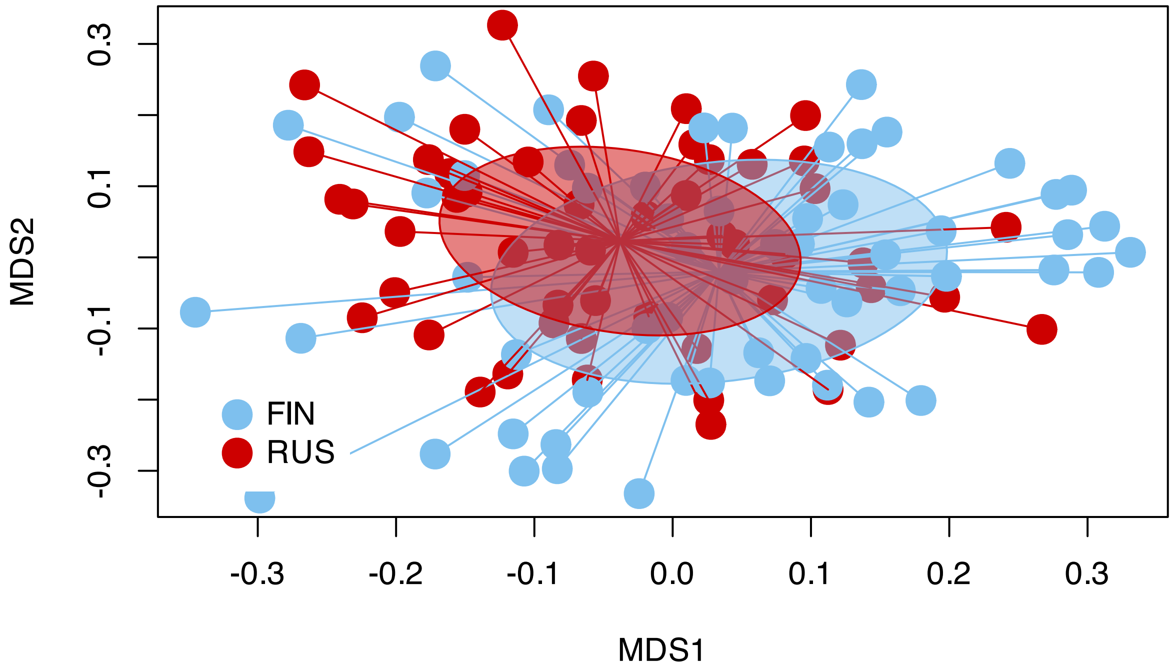

3) Variable contribution to skin microbial composition

Variables which contributed to the variation in skin microbial compositions were investigated using the permutational multivariate analysis of variance (PERMANOVA) separately in the Finnish and Russian study populations. Significant variables ($P < 0.05$) from PERMANOVA were further modelled using distance-based redundancy analysis (dbRDA) to determine which variables best explained the inter-individual dissimilarities in the skin microbiomes. PERMANOVA and dbRDA were performed on the sample-wise Bray-Curtis dissimilarities using the R-package vegan.

```
# let's first take a look at the whole data

# prokaryotes + eukaryotes ~ Location
adonis2(bray_curtis ~ Location, data = colData(tse), permutations = 999)
```

```
## Permutation test for adonis under reduced model
## Permutation: free
## Number of permutations: 999
##
## adonis2(formula = bray_curtis ~ Location, data = colData(tse), permutations = 999)
##           Df SumOfSqs      R2      F Pr(>F)
## Model      1   0.8234 0.03572 4.0742  0.001 ***
## Residual 110  22.2317 0.96428
## Total    111  23.0551 1.00000
## ---
## Signif. codes:  0 '***' 0.001 '**' 0.01 '*' 0.05 '.' 0.1 ' ' 1
```

```
# viruses ~ Location
adonis2(bray_curtis_virus ~ Location, data = colData(tse_vir), permutations = 999)
```

```
## Permutation test for adonis under reduced model
## Permutation: free
## Number of permutations: 999
##
## adonis2(formula = bray_curtis_virus ~ Location, data = colData(tse_vir), permutations = 999)
```

```
##           Df SumOfSqs      R2      F Pr(>F)
## Model      1    1.214 0.02708 3.0617  0.001 ***
## Residual 110   43.624 0.97292
## Total    111   44.838 1.00000
## ---
## Signif. codes:  0 '***' 0.001 '**' 0.01 '*' 0.05 '.' 0.1 ' ' 1
```

```
# pairwise anova for allergic sensitization categories per geographic sampling location
# prokaryotes and eukaryotes only

set.seed(446)
p_multi <- multiconstrained(method = "dbrda", formula = bray_curtis ~ loc.categ +
                              Condition(Sex + M_total_seqs + Dups_prc + Unclassified),
                            data = colData(tse), distance = "bray", comm = NULL,
                            add = FALSE, multicomp = "", contrast = 0)

p_multi
```

```
## Multiple comparisons for dbrda for all contrasts of loc.categ
##
## Model:  multiconstrained(method = "dbrda", formula = bray_curtis ~ loc.categ + Condition(Sex + M_tota
##
##                     Df SumOfSqs      F Pr(>F)
## Fin.HS vs. Fin.NS  1  0.41632 2.5700  0.008 **
## Fin.HS vs. Fin.SI  1  0.39866 2.3255  0.009 **
## Fin.HS vs. Rus.HS  1  0.54421 2.8750  0.002 **
## Fin.HS vs. Rus.NS  1  0.40215 1.9211  0.030 *
## Fin.HS vs. Rus.SI  1  0.41026 2.1367  0.011 *
## Fin.NS vs. Fin.SI  1  0.07279 0.5490  0.930
## Fin.NS vs. Rus.HS  1  0.25131 1.7895  0.078 .
## Fin.NS vs. Rus.NS  1  0.39385 2.2758  0.024 *
## Fin.NS vs. Rus.SI  1  0.22562 1.4373  0.120
## Fin.SI vs. Rus.HS  1  0.24878 1.5770  0.109
## Fin.SI vs. Rus.NS  1  0.31564 1.6158  0.102
## Fin.SI vs. Rus.SI  1  0.22353 1.3866  0.129
## Rus.HS vs. Rus.NS  1  0.18090 0.9919  0.422
## Rus.HS vs. Rus.SI  1  0.10138 0.5818  0.897
## Rus.NS vs. Rus.SI  1  0.19271 0.9755  0.435
## ---
## Signif. codes:  0 '***' 0.001 '**' 0.01 '*' 0.05 '.' 0.1 ' ' 1
```

```
# adjusted p-values
p.adjust(p_multi$`Pr(>F)`, method = "fdr")
```

```
##  [1] 0.0412500 0.0412500 0.0300000 0.0750000 0.0412500 0.9300000 0.1671429
##  [8] 0.0720000 0.1759091 0.1759091 0.1759091 0.1759091 0.5019231 0.9300000
## [15] 0.5019231
```

```
# Finnish Karelia----

set.seed(906)

results_adonis_FIN <- lapply(colnames(colData(tse_FIN)), function(x){
  form <- as.formula(paste("t(assays(tse_FIN)$Abundance)", x, sep = "~"))
  z <- adonis2(form, data = colData(tse_FIN), permutations = 999, method = "bray", na.action = na.omit)
  return(as.data.frame(z))
```

```
}
)

names(results_adonis_FIN) <- colnames(colData(tse_FIN))
results_adonis_FIN <- do.call(rbind, results_adonis_FIN)

# show the significant PERMANOVA results
subset(results_adonis_FIN[,c(3,5)], results_adonis_FIN$`Pr(>F)` < 0.05)
```

```
##                            R2 Pr(>F)
## Age.Model          0.03646313  0.017
## Sex.Model          0.04763595  0.004
## Birch.Model        0.05759778  0.003
## Horse.Model        0.03739708  0.017
## AllMix.Model       0.03655088  0.014
## Category.Model     0.05638150  0.024
## Atopic_derm.Model  0.03845615  0.017
## Dups_prc.Model     0.04272587  0.003
## loc.categ.Model    0.05638150  0.029
## Living_env.Model   0.05800824  0.038
## Unclassified.Model 0.09128417  0.001
```

```
# Russian Karelia----

set.seed(874)

results_adonis_RUS <- lapply(colnames(colData(tse_RUS)), function(x){
  form <- as.formula(paste("t(assays(tse_RUS)$Abundance)", x, sep = "~"))
  z <- adonis2(form, data = colData(tse_RUS), permutations = 999, method = "bray", na.action = na.omit)
  return(as.data.frame(z))
}
)

names(results_adonis_RUS) <- colnames(colData(tse_RUS))
results_adonis_RUS <- do.call(rbind, results_adonis_RUS)

# show the significant PERMANOVA results
subset(results_adonis_RUS[,c(3,5)], results_adonis_RUS$`Pr(>F)` < 0.05)
```

```
##                                  R2 Pr(>F)
## Sex.Model                0.04297922  0.015
## Dog_owner.Model          0.04154112  0.026
## Dog_12mo.Model           0.05170158  0.009
## Cat_12mo.Model           0.04451781  0.016
## Farm_animals_12mo.Model 0.04219311  0.017
## M_total_seqs.Model      0.04834291  0.005
## Dups_prc.Model          0.06246957  0.001
## Unclassified.Model      0.07497624  0.001
```

```
## dbRDA FIN----

set.seed(582)

dbrda_FIN <- dbrda(t(assay(tse_FIN, "Abundance")) ~ Category + Birch +
                      Condition(Sex + Age + Dups_prc),
```

```r
                    distance = "bray", data = colData(tse_FIN))

summary(dbrda_FIN)
anova.cca(dbrda_FIN, by = "terms")

# plot

col_categ_FIN <- c("orange3", "mediumpurple3", "navajowhite2")[colData(tse_FIN)$Category]

plot(dbrda_FIN, type = "n", scaling = 2, choices = c(1,2), xlab = "dbRDA1 (6.8%)", ylab = "dbRDA2 (3.5%
with(colData(tse_FIN),
     points(dbrda_FIN, display = "sites", scaling = 2,
            col = col_categ_FIN, pch = 19, cex = 2))
ordispider(dbrda_FIN, colData(tse_FIN)$Category, label = F,
           col = c("orange3", "mediumpurple3", "navajowhite2"))
ordiellipse(dbrda_FIN, colData(tse_FIN)$Category,
            col = c("orange3", "mediumpurple3", "navajowhite2"), border = c("orange3", "mediumpurple3",
legend("bottomleft", inset = 0.05, legend = c("NS","SI","HS"), pch = 19, pt.cex = 2,
       col = c("mediumpurple3", "navajowhite2","orange3"), box.lty = 0)
```

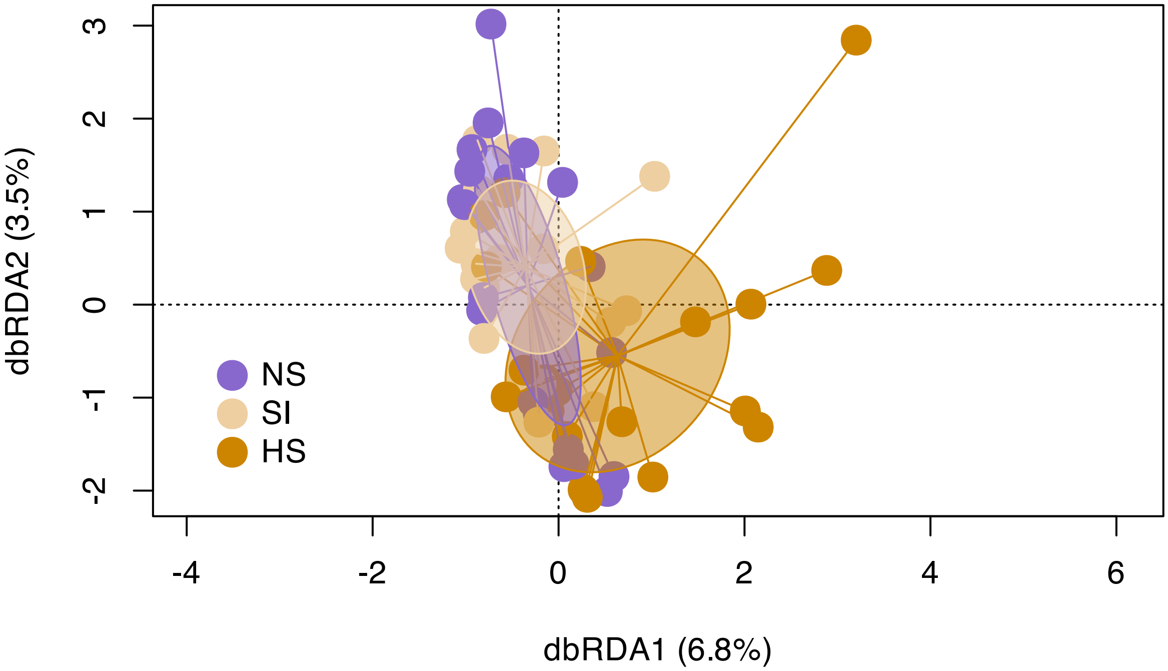


```r
## dbRDA RUS----

set.seed(165)

dbrda_RUS <- dbrda(t(assays(tse_RUS)$Abundance) ~ Dog_12mo + Cat_12mo +
                     Condition(Sex + Dups_prc + M_total_seqs),
                   distance = "bray", data = colData(tse_RUS))
summary(dbrda_RUS)
anova.cca(dbrda_RUS, by = "terms")

# plot
# color vector for interaction frequency considering both cats and dogs
```

```
pets_RUS.df <- as.data.frame(colData(tse_RUS)[,c(2,3,19:24)])
pets_RUS.df$Dog.Cat_12mo <- as.factor(interaction(pets_RUS.df$Dog_12mo,pets_RUS.df$Cat_12mo))
col_DogCat_RUS <- c("forestgreen", "cyan2", "gold1","grey63")[pets_RUS.df$Dog.Cat_12mo]

plot(dbrda_RUS, type = "n", scaling = 2, choices = c(1,2), xlab = "dbRDA1 (5.6%)", ylab = "dbRDA2 (2.2%
with(colData(tse_RUS),
     points(dbrda_RUS, display = "sites", scaling = 2,
            col = col_DogCat_RUS, pch = 19, cex = 2))
ordispider(dbrda_RUS, pets_RUS.df$Dog.Cat_12mo, label = F,
           col = c("forestgreen", "cyan2", "gold1","grey63"))
ordiellipse(dbrda_RUS, pets_RUS.df$Dog.Cat_12mo,
            col = c("forestgreen", "cyan2", "gold1","grey63"), border = c("forestgreen", "cyan2", "gold
            draw = "polygon", label = F)
legend("topright", inset = 0.02, legend = c("Dog often : Cat often","Dog often : Cat seldom", "Dog seld
       title = "Interaction frequency",pch = 19, pt.cex = 2, col = c("forestgreen", "gold1","cyan2", "g
```

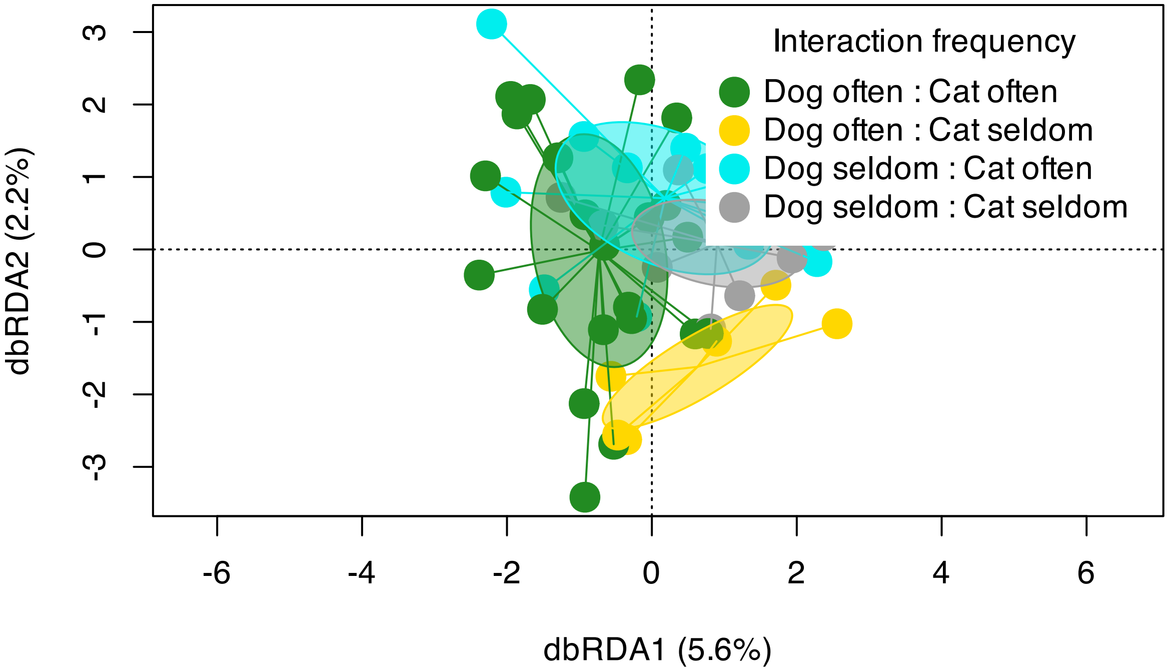

4) Differential abundance testing

To identify differentially abundant skin microbial species between the Finnish and Russian study populations, two methods, LinDA (v0.1.0) and MaAsLin2 (v1.13.0) were used. Species with prevalence lower than 10% were excluded from the differential abundance testing, and alpha level $P < 0.001$ was used in both methods. Additionally, sex was set as random effects in both LinDA and MaAsLin2 models. The compound Poisson linear model (CPLM) was used as the 'analysis_method' option in MaAsLin2. P-values were adjusted using the Benjamini-Hochberg correction. The same differential abundance testing methods were followed for identification of differentially abundant species between the Finnish non-sensitized (NS) participants and allergen sensitized participants with high allergen-specific serum IgE (HS). Due to adjusted p-values not reaching significance ($p < 0.05$), the non-adjusted values were used for identifying differentially abundant species between Finnish NS and HS participants.

```
# FIN vs RUS----
## LinDA----
```

```r
linda_Loc_out <- linda(assay(tse, "Abundance"),
                       metadata_w_tech,
                       formula = '~Location + (1|Sex)',
                       type = "proportion",
                       prev.cut = 0.1,
                       alpha = 0.001,
                       p.adj.method = "BH")

# filter out species with adjusted p-value > 0.001

linda_Loc_out_df <- as.data.frame(linda_Loc_out$output)
linda_Loc_signif <- subset(linda_Loc_out_df, linda_Loc_out_df$LocationRUS.padj < 0.001)

## MaAsLin2----

maaslin_Loc_out <- Maaslin2(input_data = t(swab_relab),
                            input_metadata = metadata_w_tech,
                            output = "MaAsLin2_Location_CPLM_out",
                            fixed_effects = "Location",
                            random_effects = "Sex",
                            normalization = "NONE",
                            transform = "NONE",
                            analysis_method = "CPLM",
                            standardize = FALSE,
                            min_prevalence = 0.1,
                            max_significance = 0.001,
                            correction = "BH")

# filter out species with adjusted p-value > 0.001

maaslin_Loc_out_df <- as.data.frame(maaslin_Loc_out$results)
maaslin_Loc_signif <- subset(maaslin_Loc_out_df, maaslin_Loc_out_df$qval < 0.001)

# shared DA taxa between LinDA and MaAsLin2

list(MaAsLin2_CPLM = maaslin_Loc_signif$feature,
     LinDA = rownames(linda_Loc_signif)) %>%
  ggvenn()
```

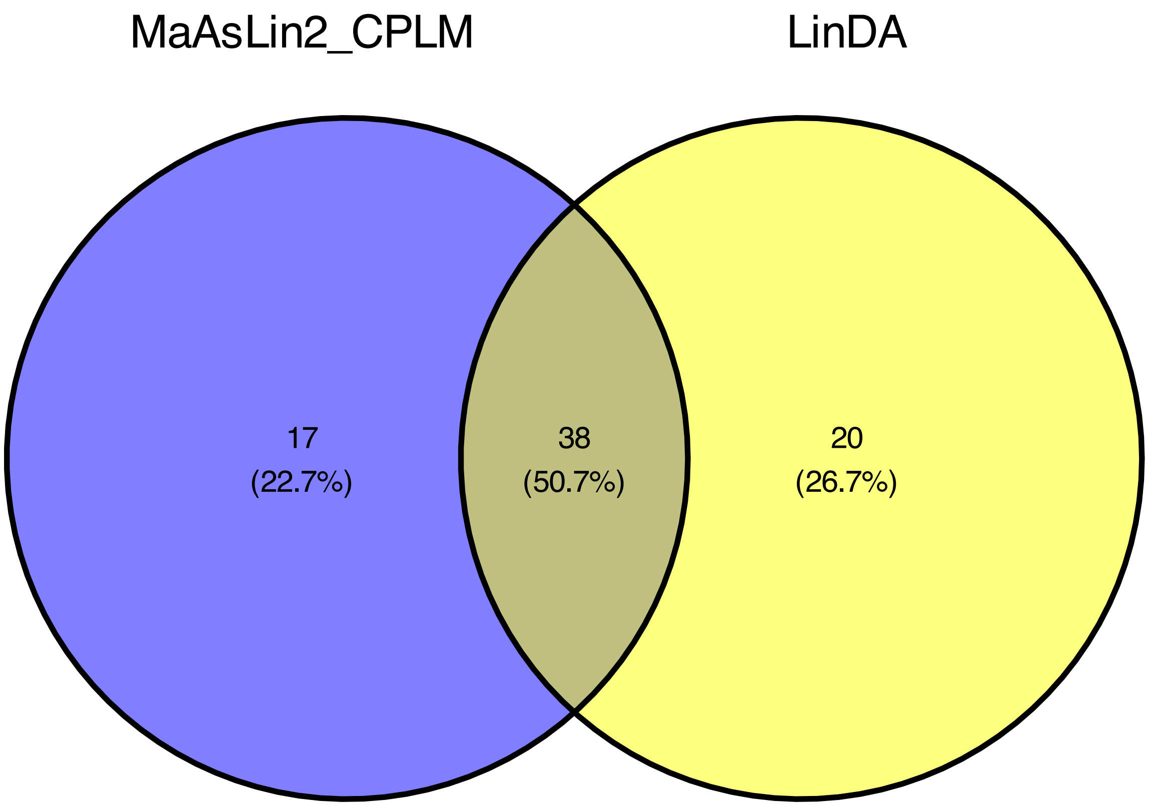


```r
# 38 DA taxa shared between the methods

### plot DA taxa identified using LinDA and MaAsLin2----

# transformed abundance counts for plotting

tse <- transformAssay(tse, assay.type = "Abundance",
                      method = "log10", name = "Log10",
                      pseudocount = 0.001)

# abundance table for the DA taxa

DA_Loc_SGB <- intersect(rownames(linda_Loc_signif), maaslin_Loc_signif$feature)
DA_Loc_tax <- rowData(tse)[which(rownames(rowData(tse)) %in% DA_Loc_SGB),]
DA_Loc_abund <- assay(tse, "Log10")[which(rownames(assay(tse, "Log10")) %in% DA_Loc_SGB),] %>%
  t() %>%
  as.data.frame()

colnames(DA_Loc_abund) <- DA_Loc_tax$Species
DA_Loc_abund$Location <- metadata_w_tech$Location

# heatmap

# annotation column and colors

annot_col_Loc <- data.frame(Location = metadata_w_tech$Location)
rownames(annot_col_Loc) <- rownames(metadata_w_tech)
```

```r
annotColor_Loc <- list(Location = c("FIN" = "skyblue2", "RUS" = "red3"))

# take out the species names of the DA taxa

names_hmap <- gsub("_", " ", names(DA_Loc_abund[,c(1:38)]))
names_hmap <- lapply(names_hmap, function(x) bquote(italic(.(x))))

# plot

pheatmap(t(DA_Loc_abund[,c(1:38)]),
         color = hcl.colors(60, "Inferno"),
         annotation_col = annot_col_Loc,
         annotation_colors = annotColor_Loc,
         fontsize_row = 8,
         labels_row = as.expression(names_hmap),
         show_colnames = F,
         annotation_names_row = F,
         annotation_names_col = F,
         cellwidth = 2, cellheight = 9)
```

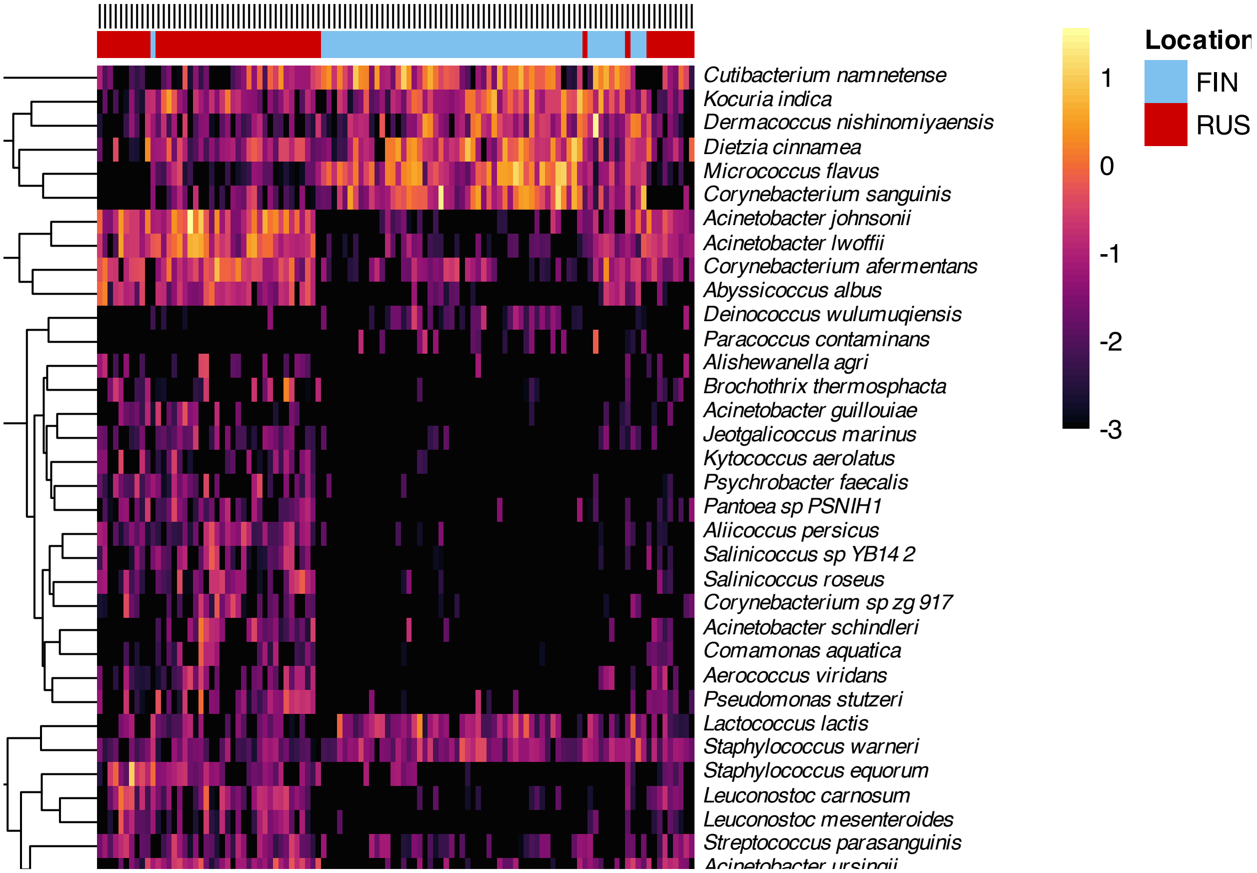


```r
# FIN NS vs HS----
# first make TSE for FIN without the allergic sensitization category 'sensitized intermediate' (SI)

tse_FIN_Allg <- tse_FIN[ ,tse_FIN$Category != "SI"]

sum(rowSums(assay(tse_FIN_Allg, "Abundance")) == 0)
```

```r
# 318 taxa are exclusively in SI samples
# remove these from FIN NS+HS TSE object

tse_FIN_Allg <- tse_FIN_Allg[rowSums(assay(tse_FIN_Allg, "Abundance")) > 0, ]

## LinDA----

linda_AllgF_out <- linda(assay(tse_FIN_Allg, "Abundance"),
                        colData(tse_FIN_Allg),
                        formula = '~Category + (1|Sex)',
                        type = "proportion",
                        prev.cut = 0.1,
                        alpha = 0.001,
                        p.adj.method = "BH")

linda_AllgF_out_df <- as.data.frame(linda_AllgF_out$output)
linda_AllgF_signif <- subset(linda_AllgF_out_df, linda_AllgF_out_df$CategoryNS.pvalue < 0.05)

## MaAsLin2----

maaslin_AllgF_out <- Maaslin2(input_data = as.data.frame(t(assay(tse_FIN_Allg, "Abundance"))),
                          input_metadata = as.data.frame(colData(tse_FIN_Allg)),
                          output = "MaAsLin2_FIN_Allg_CPLM_out",
                          fixed_effects = "Category",
                          random_effects = "Sex",
                          normalization = "NONE",
                          transform = "NONE",
                          analysis_method = "CPLM",
                          standardize = FALSE,
                          min_prevalence = 0.1,
                          max_significance = 0.001,
                          correction = "BH")

maaslin_AllgF_out_df <- as.data.frame(maaslin_AllgF_out$results)
maaslin_AllgF_signif <- subset(maaslin_AllgF_out_df, maaslin_AllgF_out_df$pval < 0.05)

# shared DA taxa between LinDA and MaAsLin2
list(MaAsLin2_CPLM = maaslin_AllgF_signif$feature,
     LinDA = rownames(linda_AllgF_signif)) %>%
  ggvenn()
```

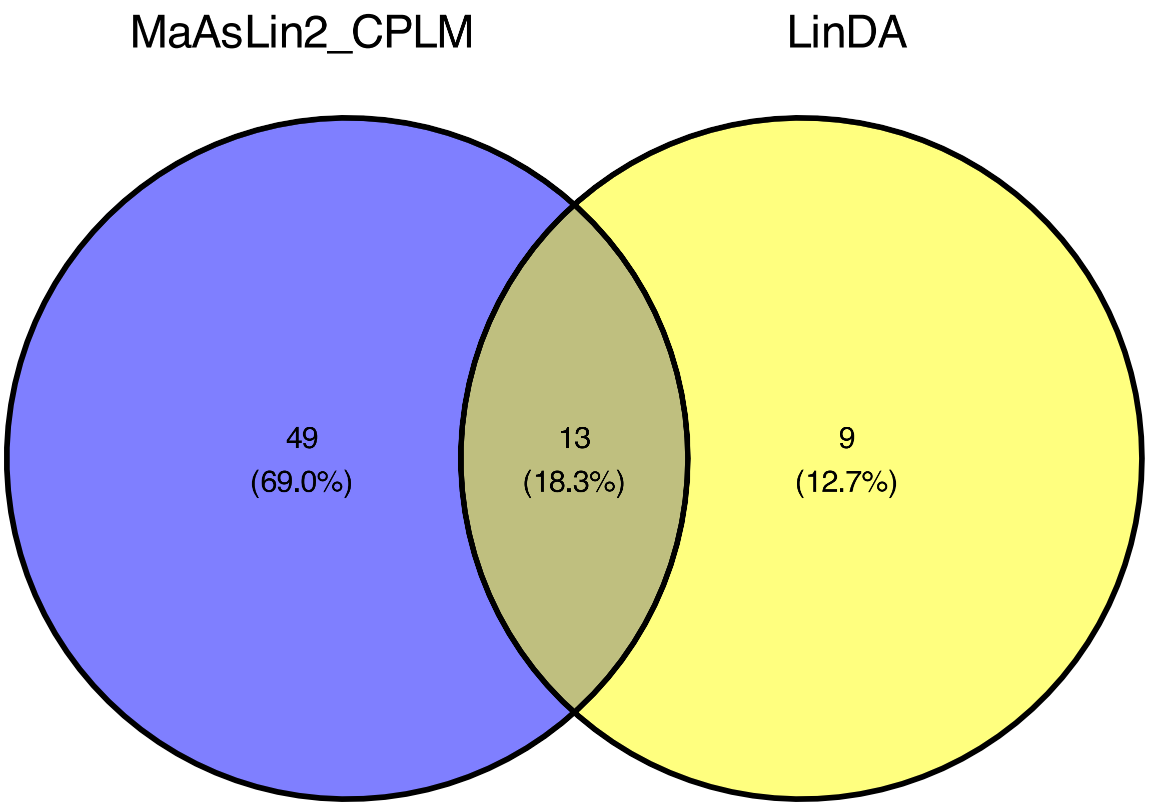


```
# 13 taxa shared between methods

### plot DA taxa identified using LinDA and MaAsLin2----

DA_AllgF_SGB <- intersect(rownames(linda_AllgF_signif), maaslin_AllgF_signif$feature)
DA_AllgF_tax <- rowData(tse_FIN_Allg)[which(rownames(rowData(tse_FIN_Allg)) %in% DA_AllgF_SGB),]
DA_AllgF_abund <- assay(tse_FIN_Allg, "Abundance")[which(rownames(assay(tse_FIN_Allg,  "Abundance")) %i
  t() %>%
  as.data.frame()

colnames(DA_AllgF_abund) <- DA_AllgF_tax$Species
DA_AllgF_abund$Category <- colData(tse_FIN_Allg)$Category

colSums(DA_AllgF_abund == 0)
# the DA taxa are mostly rare species (0 in many samples and low abundance in samples in which present)
# plot only taxa that are in at least 60% (n = 25) of the samples
# --> discard samples with > 17 0's
# Cutibacterium acnes, Malassezia globosa and Malassezia symbodialis remain

# boxplots

gather(DA_AllgF_abund, SGB, Abundance,
       c(Cutibacterium_acnes, Malassezia_globosa, Malassezia_sympodialis), factor_key = TRUE) %>%
  ggplot(., aes(x = Category, y = sqrt(Abundance), color = Category)) +
  geom_boxplot(alpha = 0) +
  geom_jitter(alpha = 0.5) +
  stat_compare_means(label.y = 7.5, label = "p.format", label.x = 1.3) +
  scale_color_manual(values = c("orange3", "mediumpurple3")) +
```

```
  facet_wrap(vars(SGB)) +
  ylab("Abundance") +
  theme_bw() +
  theme(axis.text.y = element_text(size = 6),
        axis.text.x = element_blank(),
        axis.ticks.x = element_blank(),
        axis.title.x = element_blank(),
        strip.text = element_text(size=9))
```

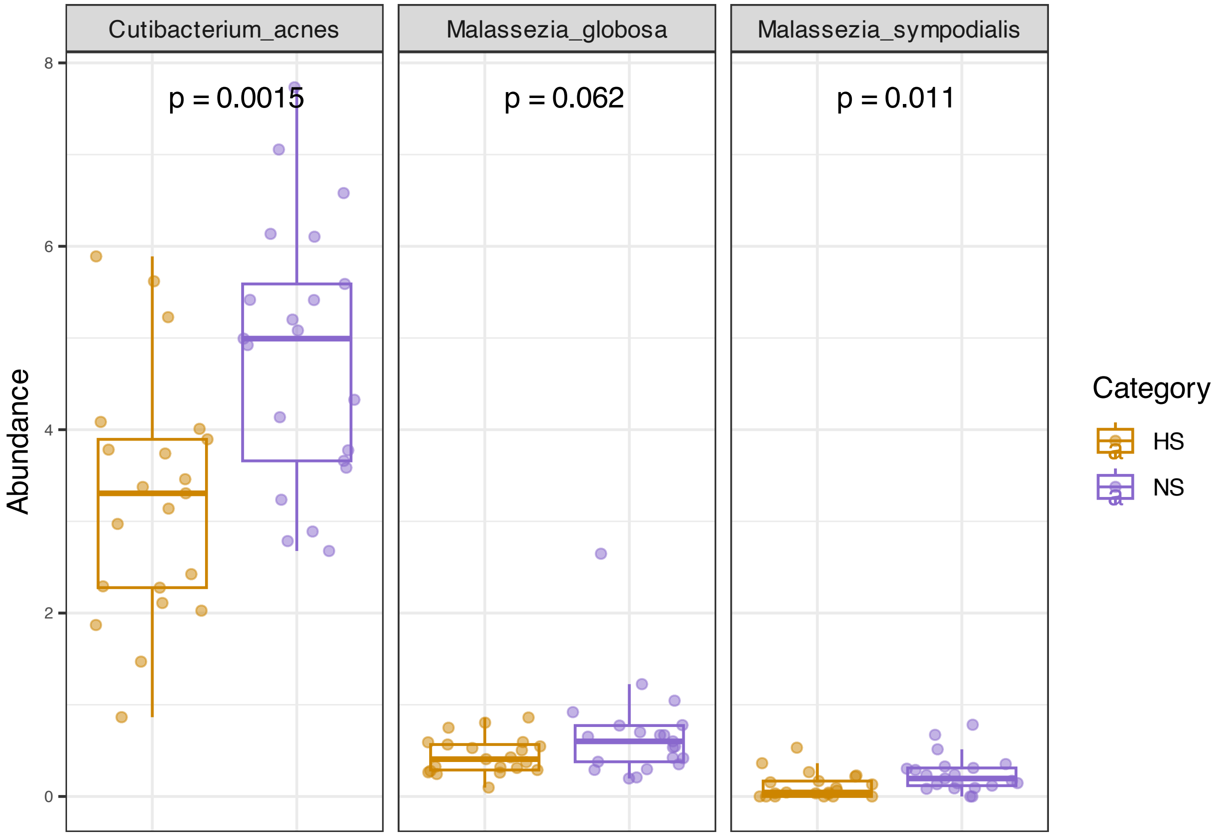


```
### plot C. acnes and Malassezia abundances----

# Agglomerate Malassezia species abundances and visualize C. acnes and Malassezia genus abundances acro

# Malassezia genus abundance

tse_Malassezia_FIN <- subsetFeatures(tse_FIN, rowData(tse_FIN)$Genus == "Malassezia")

# C. acnes species abundance

tse_C.acnes_FIN <- subsetFeatures(tse_FIN, rowData(tse_FIN)$Species == "Cutibacterium_acnes")

# merge the taxa abundance tables and add 'Category' variable

DA_AllgF_abund_2 <- cbind(as.data.frame(t(assay(tse_C.acnes_FIN, "Abundance"))),
                          data.frame(colSums(assay(tse_Malassezia_FIN, "Abundance"))))

DA_AllgF_abund_2$Category <- colData(tse_FIN)$Category
```

```r
names(DA_AllgF_abund_2) <- c("Cutibacterium acnes", "Malassezia", "Category")

# violin plot

Category_order <- c("NS", "SI", "HS")
categ_comparisons <- list(c("HS", "NS"), c("NS", "SI"), c("HS", "SI"))

ggarrange(
gather(DA_AllgF_abund_2, SGB, Abundance,
       'Cutibacterium acnes', factor_key = TRUE) %>%
  ggplot(., aes(x = factor(Category, level = Category_order), y = Abundance)) +
  geom_violin(aes(fill= Category, col = Category)) +
  geom_boxplot(width=0.1, alpha = 0.5) +
  scale_fill_manual(values=c("mediumpurple1","moccasin","darkgoldenrod2"), breaks = c("NS","SI","HS")) +
  scale_color_manual(values = c("mediumpurple4","navajowhite3","orange4"), breaks = c("NS","SI","HS")) +
  stat_compare_means(comparisons = categ_comparisons, method = "wilcox.test", label = "p.format") +
  ylab("Abundance") +
  facet_wrap(vars(SGB), labeller = labeller(SGB = c("Cutibacterium acnes", "Malassezia"))) +
  theme_bw() +
  theme(panel.grid.major = element_blank(), panel.grid.minor = element_blank()) +
  theme(legend.position = "none",
        axis.text.y = element_text(size = 8),
        axis.text.x = element_blank(),
        axis.ticks.x = element_blank(),
        axis.title.x = element_blank(),
        strip.text = element_text(size=10, face = "italic")),
  gather(DA_AllgF_abund_2, SGB, Abundance,
       Malassezia, factor_key = TRUE) %>%
  ggplot(., aes(x = factor(Category, level = Category_order), y = sqrt(Abundance))) +
  geom_violin(aes(fill= Category, col = Category)) +
  geom_boxplot(width=0.1, alpha = 0.5) +
  scale_fill_manual(values=c("mediumpurple1","moccasin","darkgoldenrod2"), breaks = c("NS","SI","HS")) +
  scale_color_manual(values = c("mediumpurple4","navajowhite3","orange4"), breaks = c("NS","SI","HS")) +
  stat_compare_means(comparisons = categ_comparisons, method = "wilcox.test", label = "p.format") +
  ylab("Abundance (sqrt)") +
  facet_wrap(vars(SGB), labeller = labeller(SGB = c("Cutibacterium acnes", "Malassezia"))) +
  theme_bw() +
  theme(panel.grid.major = element_blank(), panel.grid.minor = element_blank()) +
  theme(axis.text.y = element_text(size = 8),
        axis.text.x = element_blank(),
        axis.ticks.x = element_blank(),
        axis.title.x = element_blank(),
        strip.text = element_text(size=10, face = "italic")),
common.legend = T, legend = "right", nrow = 1)
```

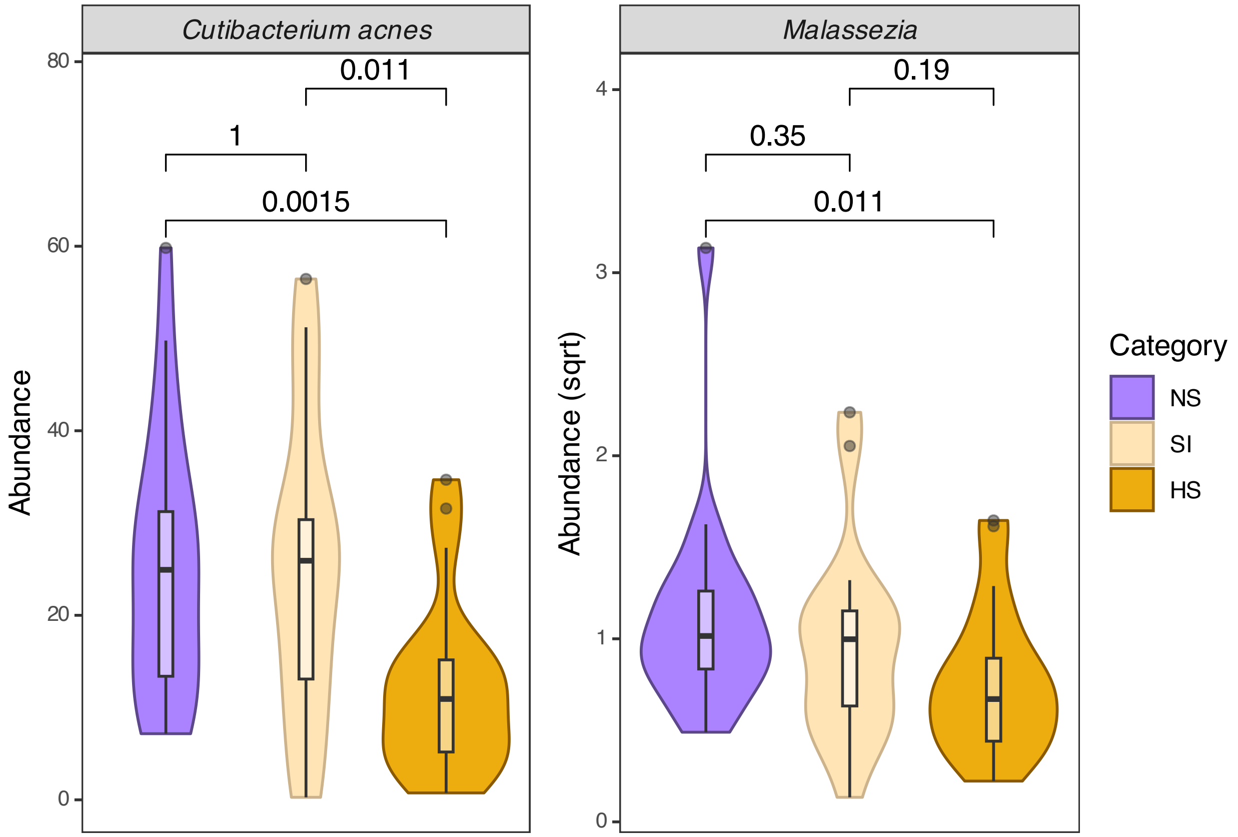

5) *Malassezia restricta* phylogenetic tree

To find if strain variation was linked to allergic sensitization categories, sample-specific strains were identified using StrainPhlAn4 (v4.0.3) and the MetaPhlAn4 markers in vJan21 database. Species markers for *Malassezia restricta* were present at sufficient coverage in 31, for which multiple sequence alignment was produced by PhyloPhlAn wihtin StrainPhlAn. IQ-TREE 2 was used to construct a maximum likelihood phylogenetic tree from the MSA.

```
# import the phylogenetic tree tree
tree_M.restricta <- read.iqtree("Additional_file_7_EUK76775_concatenated.aln.treefile")

# tip labels as four-digit sample codes to match metadata row names
mr_tiplab <- sapply(strsplit(tree_M.restricta@phylo$tip.label, "_"), `[[`,1)

# tree metadata
# subset metadata choosing columns for geographic sampling location, allergic sensitizatio category and
mr_meta <- data.frame(metadata_w_tech[rownames(metadata_w_tech) %in% mr_tiplab, c(1,12,13)])
# rename metadata rownames to match the tree tip labels
mr_meta <- mr_meta[match(mr_tiplab, rownames(mr_meta)), ]
rownames(mr_meta) <- tree_M.restricta@phylo$tip.label

# plot tree: rectangular tree with root at midpoint
p_tree <- ggtree(tree_M.restricta, layout = "rectangular", root.position = 0.5)

# visualize UFBoot and SH-aLRT value: set threshold to 70%
p_tree$data$bootstrap <-  '0'
p_tree$data[which(p_tree$data$SH_aLRT >= 70 & p_tree$data$UFboot >= 70),]$bootstrap <- '1'
```

```
p_tree2 <- p_tree + new_scale_color() +
  geom_nodepoint(aes(color=bootstrap == '1'), size = 3) +
  scale_color_manual(name='Bootstrap', values=setNames( c('black', 'grey'), c(T,F)), guide = "none")

# add annotations for the allergic sensitization category and geographic sampling location
# make a dataframe that can be combined with the p_tree$data (the tree data includes both tips (31) and
mr_meta[nrow(mr_meta) + 29, ] <- NA
rownames(mr_meta) <- p_tree2$data$label

# add annotations to the tree data
p_tree2$data$Category <- mr_meta$Category
p_tree2$data$Location <- mr_meta$Location

# Malassezia restricta
p_tree2 + new_scale_color() +
  geom_tippoint(aes(color = Category, shape = Location), size = 5) +
  scale_shape_manual(values = c(15,17), name = "Location") +
  scale_color_manual(values=c("mediumpurple3", "navajowhite2", "orange3"),
                     breaks = c("NS", "SI", "HS"),
                     name="Category", labels = c("NS", "SI", "HS"))
```

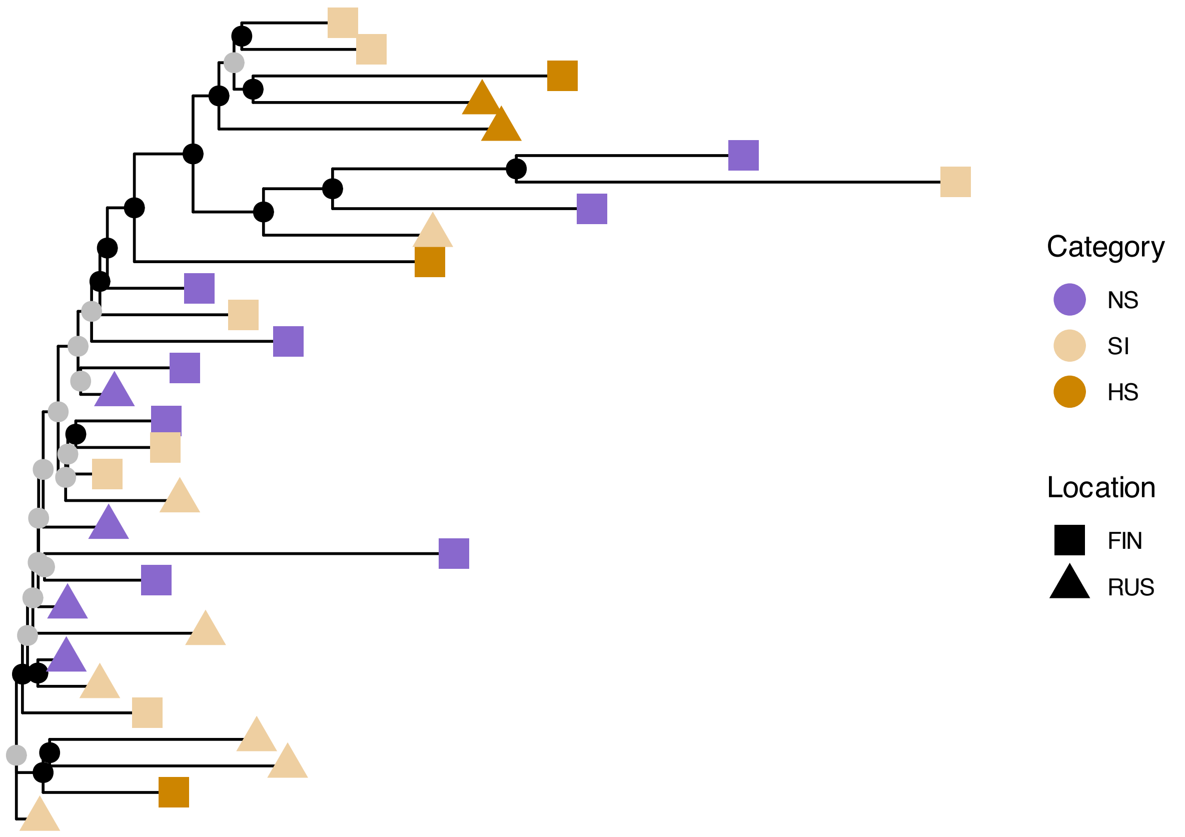


```
# phyloSignal metadata associations
# strain association with allergen specific combination sIgE
# Prepare an list object of parameters to be tested
dat <- list()
dat$Combination_sIgE <- mr_meta[c(1:31),2]+1 # a pseudocount was added to improve further visualization
```

```r
# Combine phylogeny and traits into a phylo4d object.
phyd <- phylo4d(tree_M.restricta@phylo, dat)

# Measuring and testing the signal for each trait
phyloSignal(p4d = phyd, method = "all")
```

```
## $stat
##                       Cmean             I        K   K.star    Lambda
## Combination_sIgE 0.2333572 -6.492114e-05 1.296763 1.386299 0.3816406
##
## $pvalue
##                   Cmean     I     K K.star    Lambda
## Combination_sIgE 0.027 0.009 0.015  0.004 0.2619817
```

```r
# Locating the signal with LIPA
carni.lipa <- lipaMoran(phyd)

#Visualize
# tip labels by location
tiplabs2=as.character(mr_meta$Location)
# tip colors by allergic sensitization category
tiplabs=mr_meta$Category
#colorkey by allergy category
colorkey <- c(HS = "orange3", NS = "mediumpurple3", SI = "navajowhite2")
cols <- as.character(colorkey[tiplabs])

bar_colors <- ifelse(carni.lipa$p.value < 0.05, "tomato3", "dodgerblue3") # variable-strain association
barplot.phylo4d(phyd, tree.type = "phylo",bar.col=bar_colors,
                center = FALSE , scale = FALSE, tip.labels = tiplabs2, tip.col=cols, show.tip = T,
                edge.width=2,  node.color = "black", tree.ladderize = T, tree.ratio=0.5,
                trait.bg.col = "grey93", trait.cex=0.8)
```

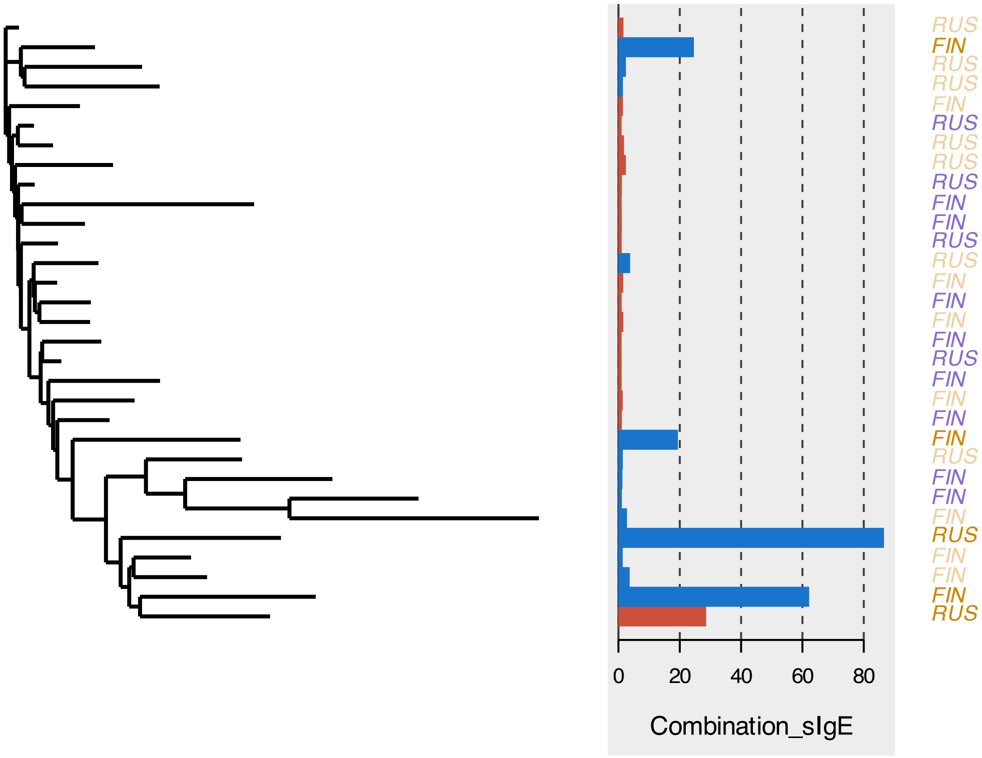

6) Network analysis

To further investigate skin microbiome differences between non-sensitized individuals and individuals with relatively high allergen-specific serum IgE, microbe-microbe co-occurrence was compared between the groups using network analysis. Microbe-microbe co-occurrence was obtained by calculating Spearman correlation between species abundances of the most abundant species (present in at least 50% of the samples) within both groups (NS and HS) separately. Networks were drawn for co-occurring species with rho > 0.6 and adjusted p-value < 0.05, and to compare network centrality, only species that met the rho and p-value criteria in both NS and HS co-occurrence networks were included. That is, NS and HS networks included the same species. To ensure that the differences in network topology and centrality were not due to distinct variation of species abundances within the NS and HS, Bray-Curtis dissimilarity was calculated within both groups using the composition of the 18 shared species and the group dispersions were compared.

```
# FIN non-sensitized participants: correlations-----

# subset NS samples and exclude taxa that are not present in the NS samples

tse_FNS <- tse_FIN[ ,tse_FIN$Category == "NS"]

tse_FNS <- tse_FNS[rowSums(assay(tse_FNS, "Abundance")) > 0, ]

# include only species that are present in at least 50% of the samples

FNS_prevtax <- getPrevalentFeatures(tse_FNS,
                                    assay.type = "Abundance",
                                    prevalence = 0.5, as_relative = T)
```

```
## Warning in getPrevalentFeatures(tse_FNS, assay.type = "Abundance", prevalence =
## 0.5, : 'getPrevalentFeatures' is deprecated.
```

```
FNS_prevtax_relab <- assay(tse_FNS, "Abundance")[
    which(rownames(assay(tse_FNS, "Abundance")) %in% FNS_prevtax),
] |> as.matrix()

# taxonomic table of the most prevalent taxa

FNS_prevtax_table <- rowData(tse_FNS)[
    which(rownames(rowData(tse_FNS)) %in% FNS_prevtax),
] |> data.frame()

# Spearman correlations

corr_FNS <- rcorr(t(FNS_prevtax_relab), type="spearman")
corrmat_FNS <- corr_FNS$r
corrP_FNS <- corr_FNS$P
padj_FNS <- p.adjust(corrP_FNS, method = "fdr")

# keep only correlations > 0.6 (positive) and with adjusted p-value < 0.05

corrmat_FNS[which(corrmat_FNS < 0.6)] <- 0
corrmat_FNS[which(padj_FNS > 0.05)] <- 0
diag(corrmat_FNS) <- 0

# remove the rows and columns that sum to 0

corrmat_FNS <- corrmat_FNS[which(rowSums(corrmat_FNS) != 0), ]
corrmat_FNS <- corrmat_FNS[, which(colSums(corrmat_FNS) != 0)]


# FIN highly sensitized participants: correlations-----

# subset HS samples and exclude taxa that are not present the HS samples

tse_FHS <- tse_FIN[ ,tse_FIN$Category == "HS"]

tse_FHS <- tse_FHS[rowSums(assay(tse_FHS, "Abundance")) > 0, ]

# include only species that are in at least 50% of the samples

FHS_prevtax <- getPrevalentFeatures(tse_FHS,
                                    assay.type = "Abundance",
                                    prevalence = 0.5, as_relative = T)
```

```
## Warning in getPrevalentFeatures(tse_FHS, assay.type = "Abundance", prevalence =
## 0.5, : 'getPrevalentFeatures' is deprecated.
```

```
FHS_prevtax_relab <- assay(tse_FHS, "Abundance")[
    which(rownames(assay(tse_FHS, "Abundance")) %in% FHS_prevtax),
] |> as.matrix()

# taxonomic table of the most prevalent taxa

FHS_prevtax_table <- rowData(tse_FHS)[
    which(rownames(rowData(tse_FHS)) %in% FHS_prevtax),
```

```r
] |> data.frame()

# Spearman correlations

corr_FHS <- rcorr(t(FHS_prevtax_relab), type="spearman")
corrmat_FHS <- corr_FHS$r
corrP_FHS <- corr_FHS$P
padj_FHS <- p.adjust(corrP_FHS, method = "fdr")

# keep only correlations of > 0.6 (positive) and with adjusted p-value < 0.05

corrmat_FHS[which(corrmat_FHS < 0.6)] <- 0
corrmat_FHS[which(padj_FHS > 0.05)] <- 0
diag(corrmat_FHS) <- 0

# remove the rows and columns that sum to 0

corrmat_FHS <- corrmat_FHS[which(rowSums(corrmat_FHS) != 0), ]
corrmat_FHS <- corrmat_FHS[, which(colSums(corrmat_FHS) != 0)]

# networks----

# build networks for NS and HS based on species which are present in both groups
# according to the filtering criteria (rho > 0.6 and adjusted p-value < 0.05)

# remove species that are exclusively in NS or HS

HS_SGB <- setdiff(rownames(corrmat_FHS), rownames(corrmat_FNS))

NS_SGB <- setdiff(rownames(corrmat_FNS), rownames(corrmat_FHS))

corrmat_shFHS <- corrmat_FHS[which(rownames(corrmat_FHS) %nin% HS_SGB),
                             which(rownames(corrmat_FHS) %nin% HS_SGB)]

corrmat_shFNS <- corrmat_FNS[which(rownames(corrmat_FNS) %nin% NS_SGB),
                             which(rownames(corrmat_FNS) %nin% NS_SGB)]

shFNS_g <- graph_from_adjacency_matrix(corrmat_shFNS, diag = F, mode = "undirected", weighted = T)
plot(shFNS_g, vertex.label = NA) # the network is fragmented
```

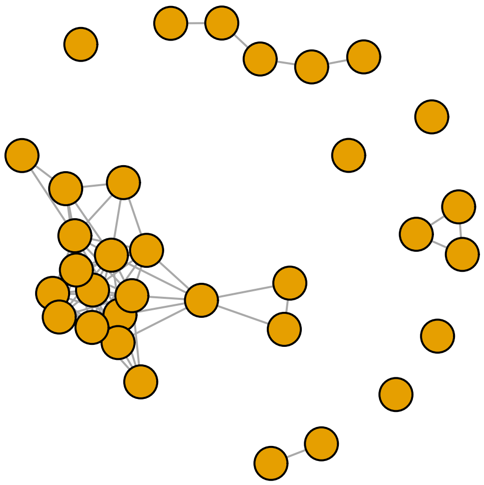

```
shFHS_g <- graph_from_adjacency_matrix(corrmat_shFHS, diag = F, mode = "undirected", weighted = T)
plot(shFHS_g, vertex.label = NA) # the network is fragmented
```

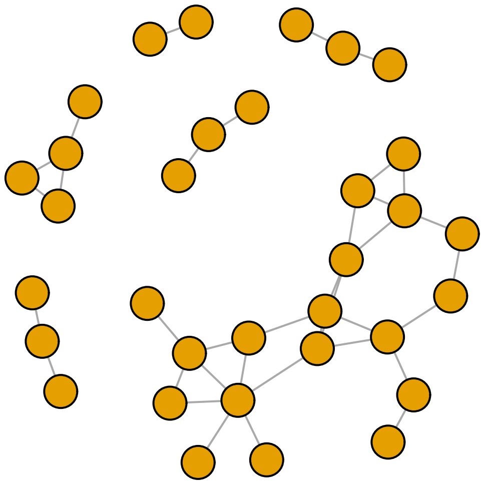

```
# compare centralities only within the giant component of each network
# extract giant component

shFNS_comps <- decompose(shFNS_g)
lengths(shFNS_comps) |> table()
```

```
##
##  1  2  3  5 18
##  5  1  1  1  1
```

```
shFNS_giant <- shFNS_comps[[3]]

shFHS_comps <- decompose(shFHS_g)
lengths(shFHS_comps) |> table()
```

```
##
##  2  3  4 18
##  1  3  1  1
```

```
shFHS_giant <- shFHS_comps[[4]]
```

```r
## centrality scores----
# make a data frame with centrality scores

shFNS_cdf <- data.frame(
    SGB = as.character(V(shFNS_giant)$name),
    Degree = as_adjacency_matrix(shFNS_giant, attr = "weight") |>
        as.matrix() |>
        rowSums(),
    Closeness = closeness(shFNS_giant),
    Betweenness = betweenness(shFNS_giant, directed = F),
    Eigen = eigen_centrality(shFNS_giant)$vector,
    Hub = hub_score(shFNS_giant)$vector
)
```

```
## Warning: `hub_score()` was deprecated in igraph 2.0.3.
## i Please use `hits_scores()` instead.
## This warning is displayed once every 8 hours.
## Call `lifecycle::last_lifecycle_warnings()` to see where this warning was
## generated.
```

```r
shFHS_cdf <- data.frame(
    SGB = as.character(V(shFHS_giant)$name),
    Degree = as_adjacency_matrix(shFHS_giant, attr = "weight") |>
        as.matrix() |>
        rowSums(),
    Closeness = closeness(shFHS_giant),
    Betweenness = betweenness(shFHS_giant, directed = F),
    Eigen = eigen_centrality(shFHS_giant)$vector,
    Hub = hub_score(shFHS_giant)$vector
)

# compare centrality scores

# merge centrality data frames

shFNS_cdf$Category <- rep("NS", 18)
shFHS_cdf$Category <- rep("HS", 18)

merged_shFIN_cdf <- rbind(shFNS_cdf, shFHS_cdf)

# plot differences

Category_order <- c("NS","HS")

gather(merged_shFIN_cdf, centrality, score,
       Degree:Betweenness, factor_key = TRUE) %>%
    ggplot(., aes(x = factor(Category, level = Category_order), y = score)) +
    geom_violin(aes(fill = Category, col = Category)) +
    geom_boxplot(width=0.1, alpha = 0.5) +
    scale_fill_manual(values=c("darkgoldenrod2", "mediumpurple1")) +
    scale_color_manual(values = c("orange4", "mediumpurple4")) +
    facet_wrap(vars(centrality), scales = "free") +
    stat_compare_means(label = "p.format", label.x = 1.3, size = 3) +
    labs(y = "Centrality score") +
```

```
    theme_pubr() +theme_bw() +
    theme(panel.grid.major = element_blank(), panel.grid.minor = element_blank()) +
    theme(axis.text.y = element_text(size = 8),
          axis.text.x = element_blank(),
          axis.ticks.x = element_blank(),
          axis.title.x = element_blank(),
          strip.text = element_text(size=10))
```

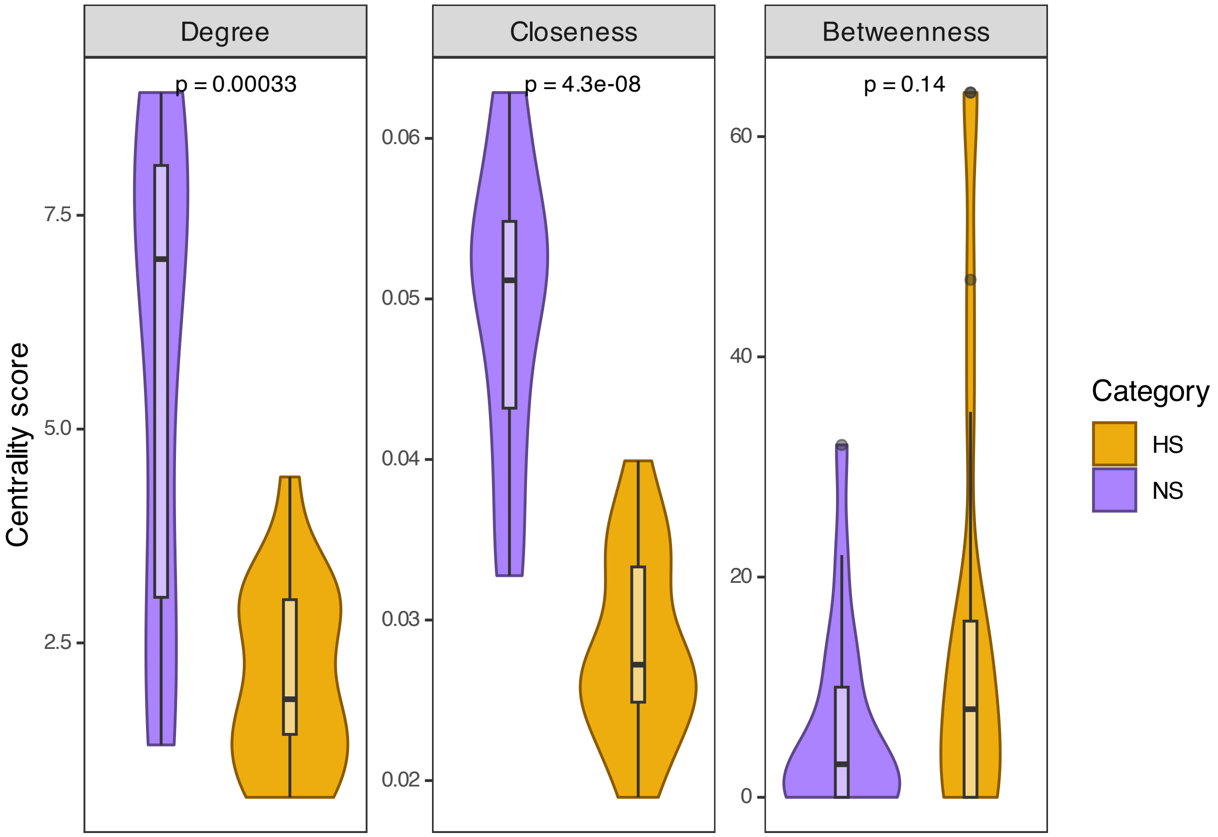


```
## node attributes----

# add species names as node labels

shFNS_taxa <- as.data.frame(rowData(tse_FIN)[which(rownames(rowData(tse_FIN)) %in%
                                                       V(shFNS_giant)$name),])

shFHS_taxa <- as.data.frame(rowData(tse_FIN)[which(rownames(rowData(tse_FIN)) %in%
                                                       V(shFHS_giant)$name),])

V(shFNS_giant)$species <- shFNS_taxa$Species
V(shFNS_giant)$species <- sub("_", " ", V(shFNS_giant)$species)

V(shFHS_giant)$species <- shFHS_taxa$Species
V(shFHS_giant)$species <- sub("_", " ", V(shFHS_giant)$species)

# walktrap clustering

shFNS_cw = cluster_walktrap(shFNS_giant)
```

```r
shFHS_cw = cluster_walktrap(shFHS_giant)

# set clustering to node attributes
# we are interested only in clusters of three or more species
# therefore clusters of < 3 species are set to 'NA'

table(shFNS_cw$membership)
```

```
##
## 1 2 3 4 5
## 3 9 3 2 1
```

```r
V(shFNS_giant)$Cluster <- shFNS_cw$membership

shFNS_tbl <- as_tbl_graph(shFNS_giant) |>
    mutate(Cluster = replace(Cluster, which(Cluster == 4), NA)) |>
    mutate(Cluster = replace(Cluster, which(Cluster == 5), NA))

table(shFHS_cw$membership)
```

```
##
## 1 2 3 4 5
## 7 4 3 2 2
```

```r
V(shFHS_giant)$Cluster <- shFHS_cw$membership

shFHS_tbl <- as_tbl_graph(shFHS_giant) |>
    mutate(Cluster = replace(Cluster, which(Cluster == 4), NA)) |>
    mutate(Cluster = replace(Cluster, which(Cluster == 5), NA))

## network visualization----

set_graph_style(plot_margin = margin(1,1,1,1))

ggraph(shFNS_tbl) +
    geom_edge_link(color = "gray80", alpha = 0.8, aes(linewidth = weight)) +
    geom_node_point(shape = 21, aes(fill = factor(Cluster), color = factor(Cluster)), size = 9) +
    scale_fill_manual(values = c("lightsteelblue2","plum1","lightgoldenrod1")) +
    scale_color_manual(values = c("cornflowerblue","orchid3","goldenrod2")) +
    geom_node_text(aes(label = species), repel = T, fontface = "bold.italic") +
    labs(col = "Cluster", fill = "Cluster") +
    scale_edge_width(name = "Corr. (rho)")
```

```
## Using "stress" as default layout
```

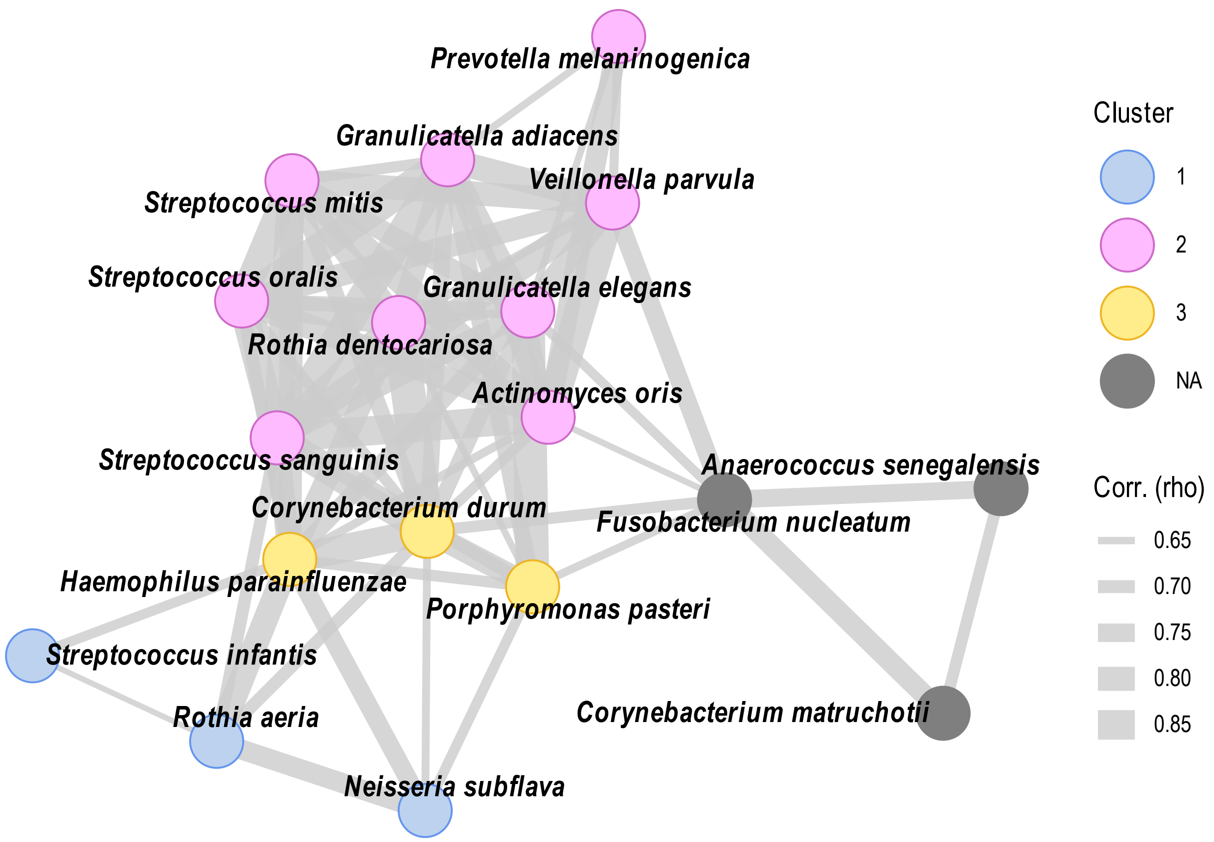


```
ggraph(shFHS_tbl) +
    geom_edge_link(color = "gray80", alpha = 0.8, aes(linewidth = weight)) +
    geom_node_point(shape = 21, aes(fill = factor(Cluster),  color = factor(Cluster)), size = 9) +
    scale_fill_manual(values = c("lightsteelblue2","plum1","lightgoldenrod1")) +
    scale_color_manual(values = c("cornflowerblue","orchid3","goldenrod2")) +
    geom_node_text(aes(label = species), repel = T, fontface = "bold.italic") +
    labs(col = "Cluster", fill = "Cluster") +
    scale_edge_width(name = "Corr. (rho)")
```

```
## Using "stress" as default layout
```

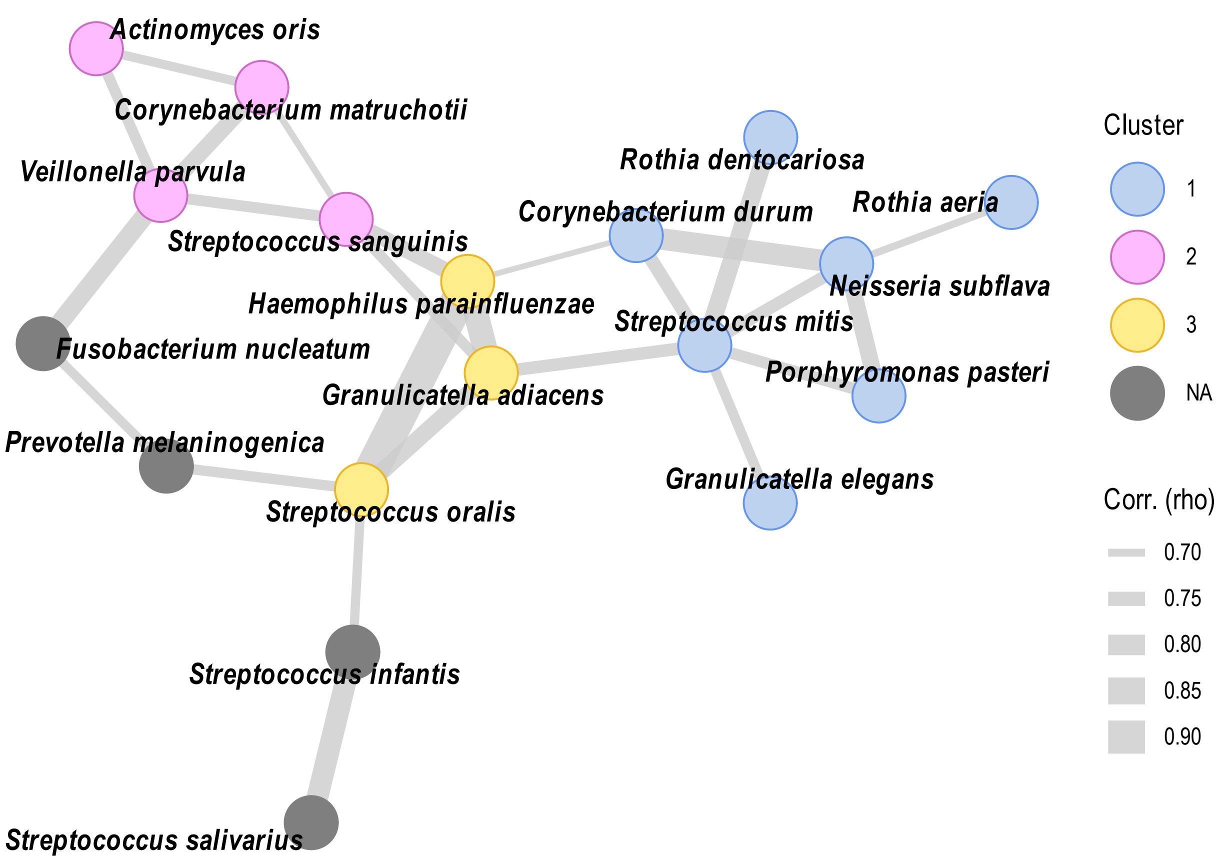


```r
# betadisper----

# subset NS and HS samples

tse_FIN_Allg <- tse_FIN[ ,tse_FIN$Category != "SI"]

tse_FIN_Allg <- tse_FIN_Allg[rowSums(assay(tse_FIN_Allg, "Abundance")) > 0, ]

# subset abundances of the 18 species share between NS and HS significantly correlating species

shFIN_relabu <- as.data.frame(assay(tse_FIN_Allg, "Abundance")[which(rownames(assay(tse_FIN_Allg, "Abun
                                                       merged_shFIN_cdf$SGB),])

# Bray-Curtis dissimilarity
# remove sample 3536 since all 18 species had abundance of 0

bc_shFIN <- vegdist(t(shFIN_relabu)[-17,], method = "bray")

# betadisper

betadisper_shFIN <- betadisper(bc_shFIN, colData(tse_FIN_Allg)[-17,13])
boxplot(betadisper_shFIN)
```

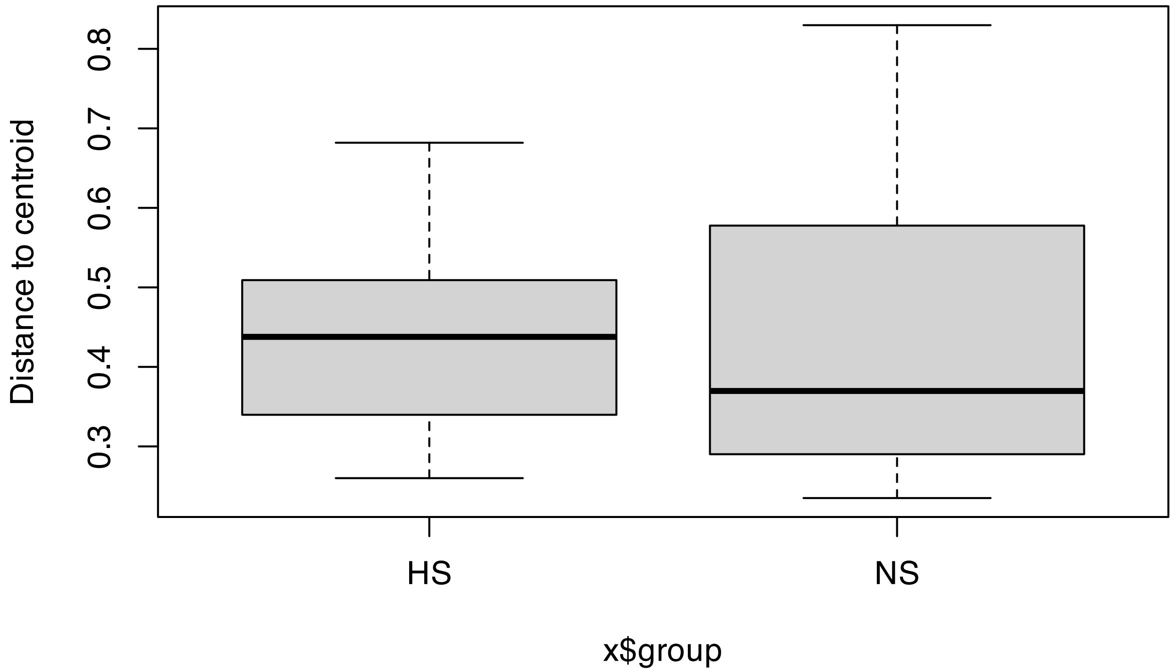


```
permutest(betadisper_shFIN) # differences in variance between HS and NS is not significant
```

```
##
## Permutation test for homogeneity of multivariate dispersions
## Permutation: free
## Number of permutations: 999
##
## Response: Distances
##           Df  Sum Sq  Mean Sq      F N.Perm Pr(>F)
## Groups     1 0.00011 0.000113 0.0053    999  0.933
## Residuals 39 0.83634 0.021445
```
