# Supplementary material for: Shotgun metagenomics reveals distinct skin microbial species in allergen-sensitized individuals
Source: Microb Genom. 2025 Dec 3;11(12):001527. doi: 10.1099/mgen.0.001527 (PMC13293315; doi:10.1099/mgen.0.001527)
Supplement: Uncited Supplementary Material 2. [file mgen-11-01527-s002.pdf]

Supplementary information

Shotgun metagenomics reveals distinct skin microbial species in allergen sensitized individuals

Matilda Riskumäki*, Matti O. Ruuskanen, Kuunsäde Mäenpää, Lasse Ruokolainen, Mika J. Mäkelä, Pekka Jousilahti, Erkki Vartiainen, Noora Ottman, Tiina Laatikainen, Tari Haahtela, Harri Alenius, Nanna Fyhrquist, Hanna Sinkko

*Corresponding author: Matilda Riskumäki (matilda.riskumaki@helsinki.fi)

Table of Contents

1. The use of mock communities ... 1

2. Supplementary figures ... 2

Figure S1. Allergic sensitization categories. ... 3
Figure S2. Overview of study population characteristics. ... 4
Figure S3. Skin microbiome composition overview. ... 5
Figure S4. Microbial mock community theoretical and identified compositions. ... 6
Figure S5. Functional profiles differ between NS and HS Finnish participants. (A) ... 7
Figure S6. Microbiome composition and diversity differ between Finnish and Russian Karelia ... 8
Figure S7. Fraction of unclassified reads in samples from Finnish and Russian Karelia. ... 9
Figure S8. Likelihood-mapping analysis for *Malassezia restricta* and *Cutibacterium acnes* strains. .. 9
Figure S9. Variance partitioning of species abundances ... 10
Figure S10. Comparison of network centrality scores between the NS and HS networks. ... 10

3. References ... 11

1. The use of mock communities

DNA was extracted from two microbial mock community samples to observe DNA extraction and sequencing performance, respectively. The microbial mock community samples included the ZymoBIOMICS Microbial Community Standard II (Zymo Research) and the Skin Microbiome Whole Cell Mix (MSA-2005, ATCC). The mock community samples were subjected to the same DNA extraction procedure as the skin swab samples, followed by library preparation with seven PCR cycles and shotgun sequencing. Sequencing data from the mock community samples was quality filtered and taxonomically classified alongside the swab samples using the same tools and parameters (KneadData and MetaPhlAn4, respectively).

Analysis of the mock community sequence data revealed differences between the theoretical composition of the mock communities and the identified compositions after DNA extraction, sequencing and sequencing data processing (Fig S4). In the ZymoBIOMICS mock community,

the species with theoretical relative abundance below 0.00089% could not be detected after sequence processing. Additionally, species that were not included in the ZymoBIOMICS mock community, including *Listeria marthii, Listeria innocua, Bacillus vallismortis* and *Bacillus tequilensis*, were identified. This could be due to misidentification of some of the reads originated from the species *Listeria monocytogenes* and *Bacillus subtilis* that were included in the mock community (Fig S4A).

Reads identified as *Dechlorosoma suillum* were detected both in ATCC and ZymoBIOMICS mock communities in minute abundance of 0.0002% and 0.00006%, respectively, and were considered as contamination in the mock samples. However, *D. suillum* was identified only in one out of the three DNA extraction occasions within both mock communities. *D. suillum* was present in 75% of the skin samples in low abundances (on average 0.019%) but not detected in the negative control samples. This suggests that *D. suillum* is not a contaminant from DNA extraction or sequencing but may possibly be cross-contamination from the skin samples to the mock community samples. Approximately 7% of the reads remained unclassified within both mock communities, suggesting that some noise was generated during sequencing or sequence processing (Fig S4).

Results from the mock community samples suggest that the rarest species in the samples may not have been identified. Moreover, identified species composition of the participants' skin swabs collected may be slightly biased from the true sampled composition on skin. Bias in the identified composition can have different origins, including DNA extraction procedure, the amount of template DNA used for sequence library preparation and the choice of library preparation kit [1, 2]. Further, sequencing data processing, such as choice of read trimming and filtering parameters, as well as the choice of taxonomic classification tool have been reported to potentially bias the composition [2]. This study does not focus on the source of the compositional bias in the samples, but the mock communities were used to ensure that reliable data is recovered after DNA extraction, sequencing and sequence processing.

2. Supplementary figures

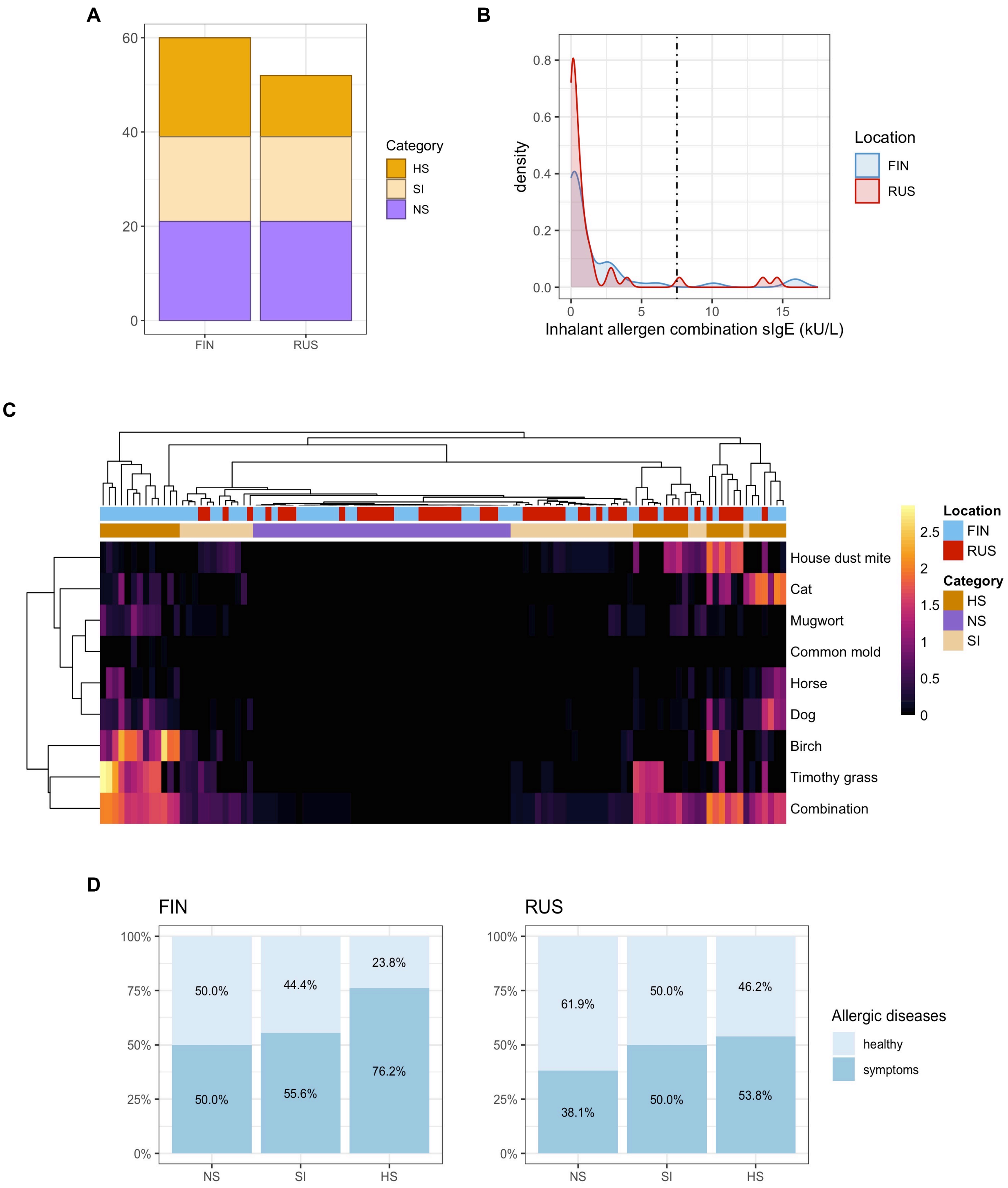

Figure S1. Allergic sensitization categories. (A) The selected subgroup study participants (n = 112) were divided into three allergic sensitization categories: NS = non-sensitized, SI = sensitized intermediate and HS = highly sensitized. (B) Threshold for HS was set to 7.5 kU/L (dashed vertical line) according to the distribution of allergen-specific serum IgE (sIgE) levels against a combination of eight inhalant allergens. The x-axis scale was cropped at 20 kU/L. (C) The participants' (x-axis) allergen-specific sIgE levels against a combination of eight inhalant allergens and each allergen separately in a clustered heatmap. The values were transformed with base ten logarithm**.** Specific sIgE against each individual allergen was not available for the NS participants and was set to zero (0). (D) The fraction of participants per allergic sensitization category from Finnish Karelia (left) and Russian Karelia (right) reporting symptoms of allergic diseases, including hay fever, asthma and atopic dermatitis, during their lifetime.

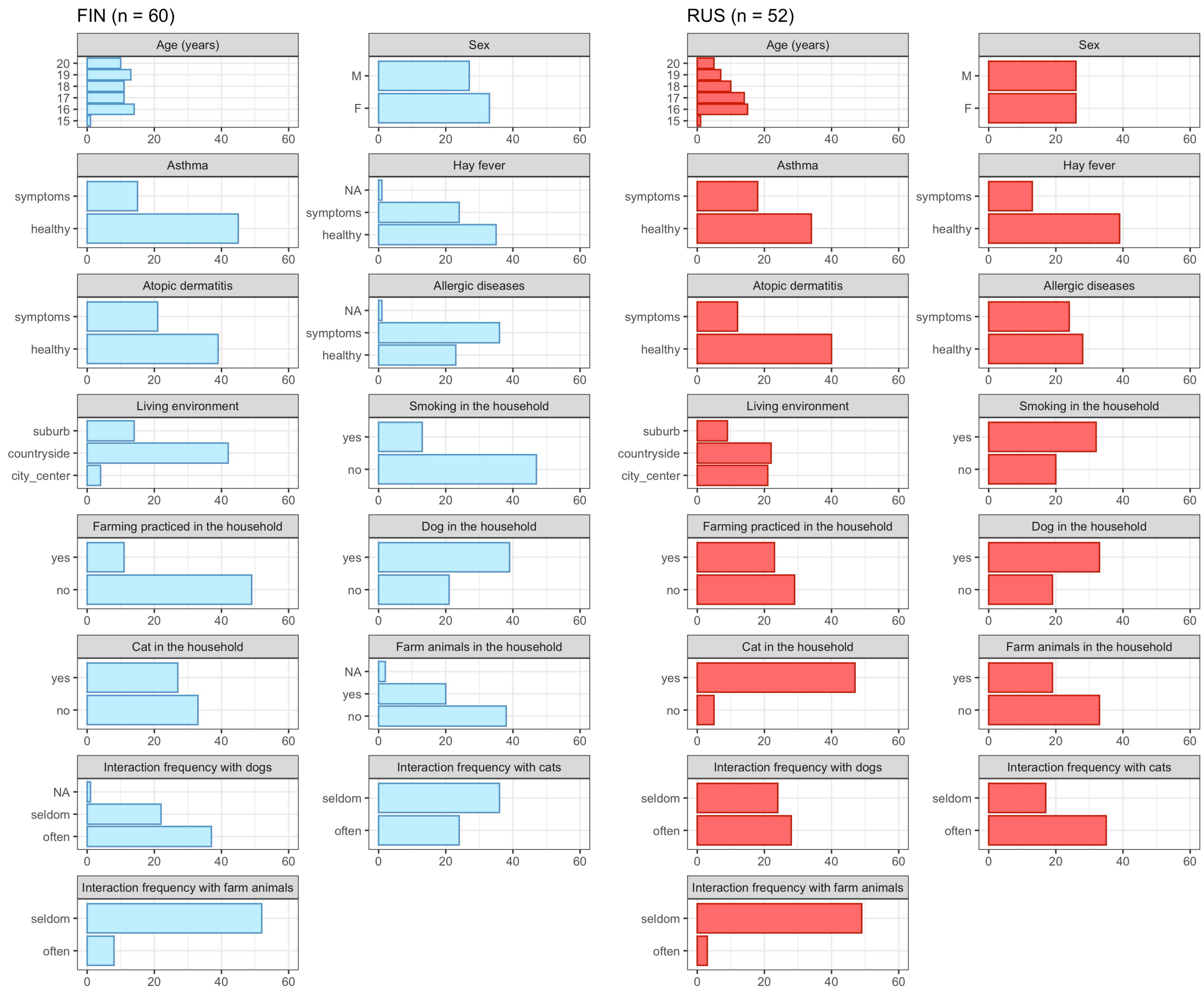

Figure S2. Overview of study population characteristics. Data from survey questionnaires was utilized for the investigation of allergic symptoms and lifestyles of the study participants. Altogether 15 variables were selected from the questionnaire to represent the selected subgroup study population (n = 112) characteristics**.** Ownership of dogs, cats and farm animals was considered as positive, if there ever was such pet in the participants' household during their childhood or at the time of sampling. Interaction frequency with household pets and farm animals defined as "often" if interaction took place on a daily or weekly basis within the past 12 months, and otherwise as "seldom".

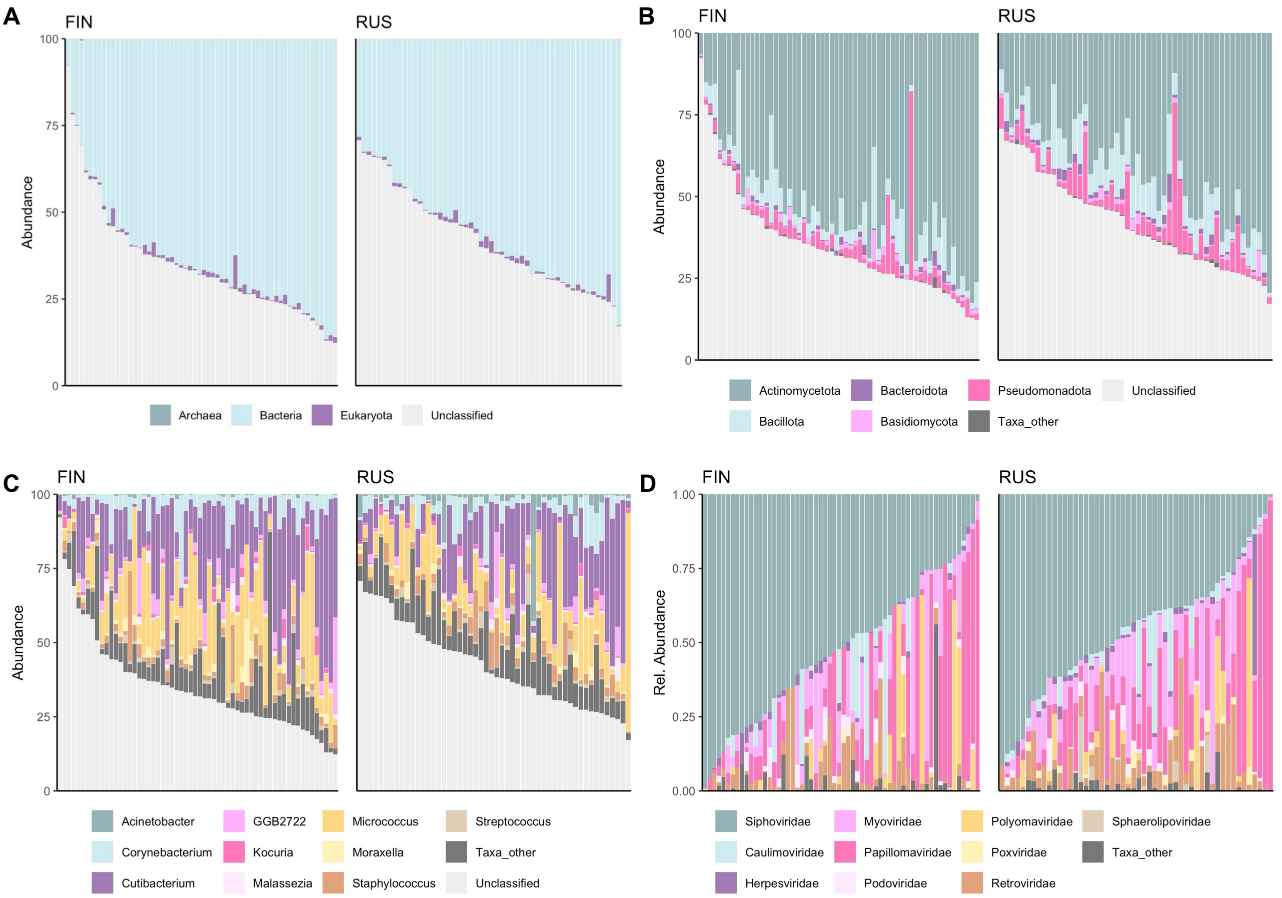

Figure S3. Skin microbiome composition overview. Per-sample (FIN: n = 60; RUS: n = 52) fraction of unclassified reads and relative abundances of taxonomies identified using (A–C) MetaPhlAn4 which included taxonomies within the domains (A) *Bacteria, Archaea* and *Eukaryota*. (B) Five most abundant families and (C) ten most abundant genera. (D) Per-sample scaled relative abundaces of the ten most abundant viral families identified using MetaPhlAn3. Note that the bacteriophage taxonomies *Siphoviridae, Myoviridae* and *Podoviridae* are outdated [3].

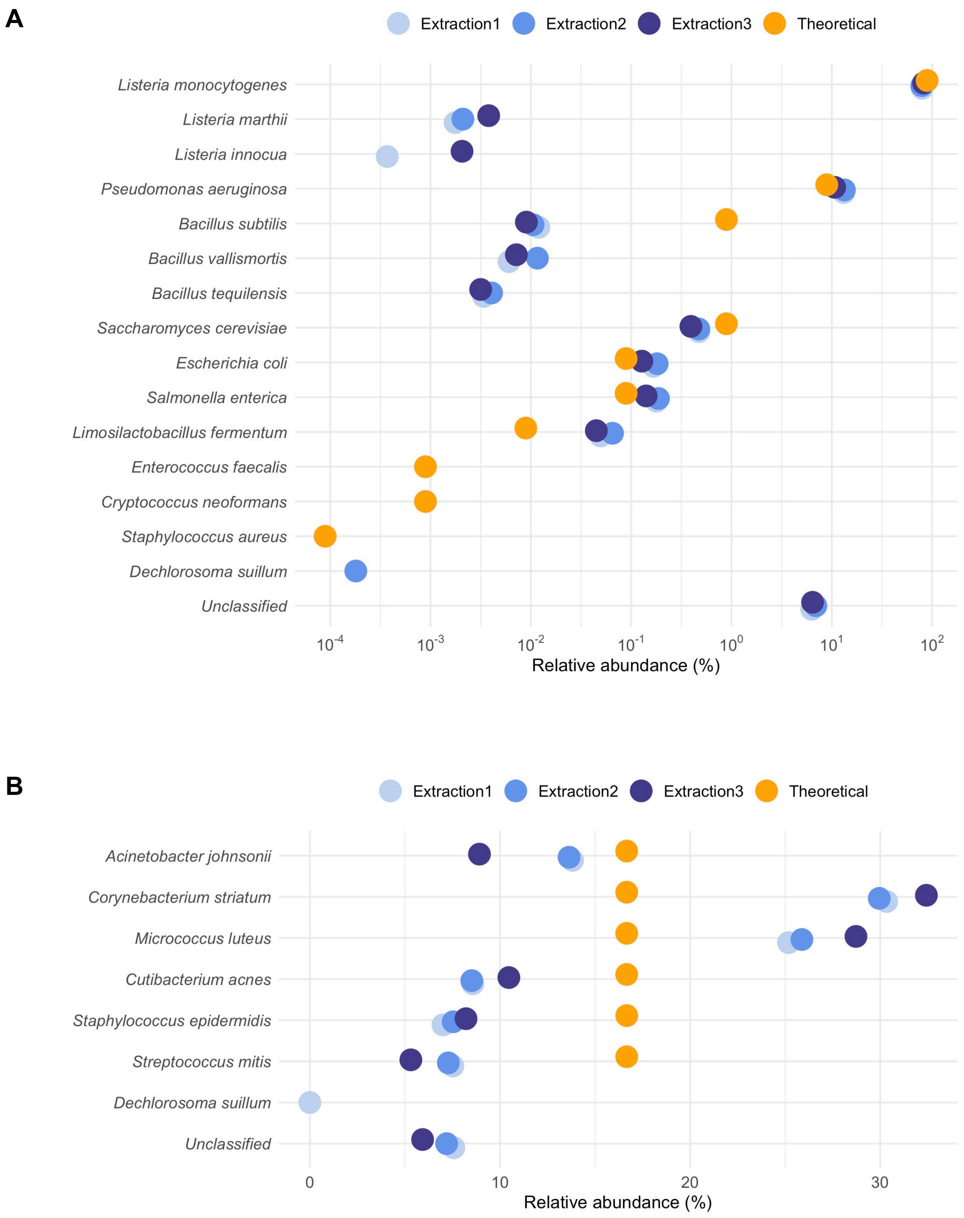

Figure S4. Microbial mock community theoretical and identified compositions. DNA was extracted on three occasions (Extraction 1–3 in shades of blue) from (A) ZymoBIOMICS Microbial Community Standard II and (B) ATCC Skin Microbiome Whole Cell Mix. (A–B) The theoretical composition, based on genomic DNA composition in A and whole cell concentration in B, is shown in yellow and the theoretically present species written in bold font.

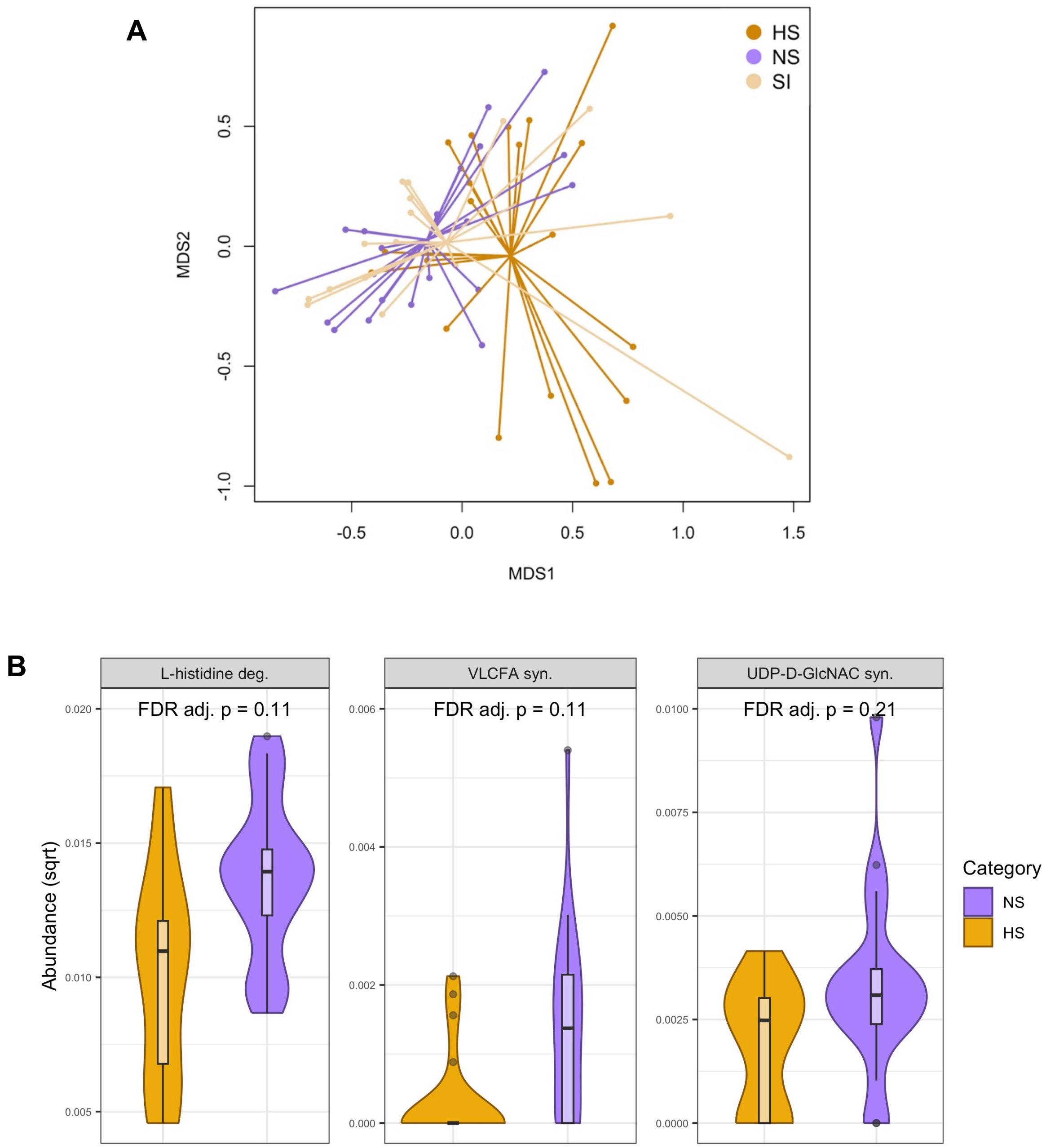

Figure S5. Functional profiles differ between NS and HS Finnish participants. (A) Differences regarding bacterial, eukaryotic and archaeal microorganisms in non-metric multidimensional scaling on sample-wise Bray-Curtis dissimilarities, stress value 0.10. (B) Pathway abundance (square root transformed) of L-histidine degradation II (left), very long chain fatty acid biosynthesis I (middle) and UDP-*N*-acetyl-D-glucosamine biosynthesis II (right) in Finnish participants by allergic sensitization category (NS: non-sensitized; SI: sensitized intermediate; HS: highly sensitized), FDR adjusted *P*-values derived from the MaAsLin2 differential abundance analysis.

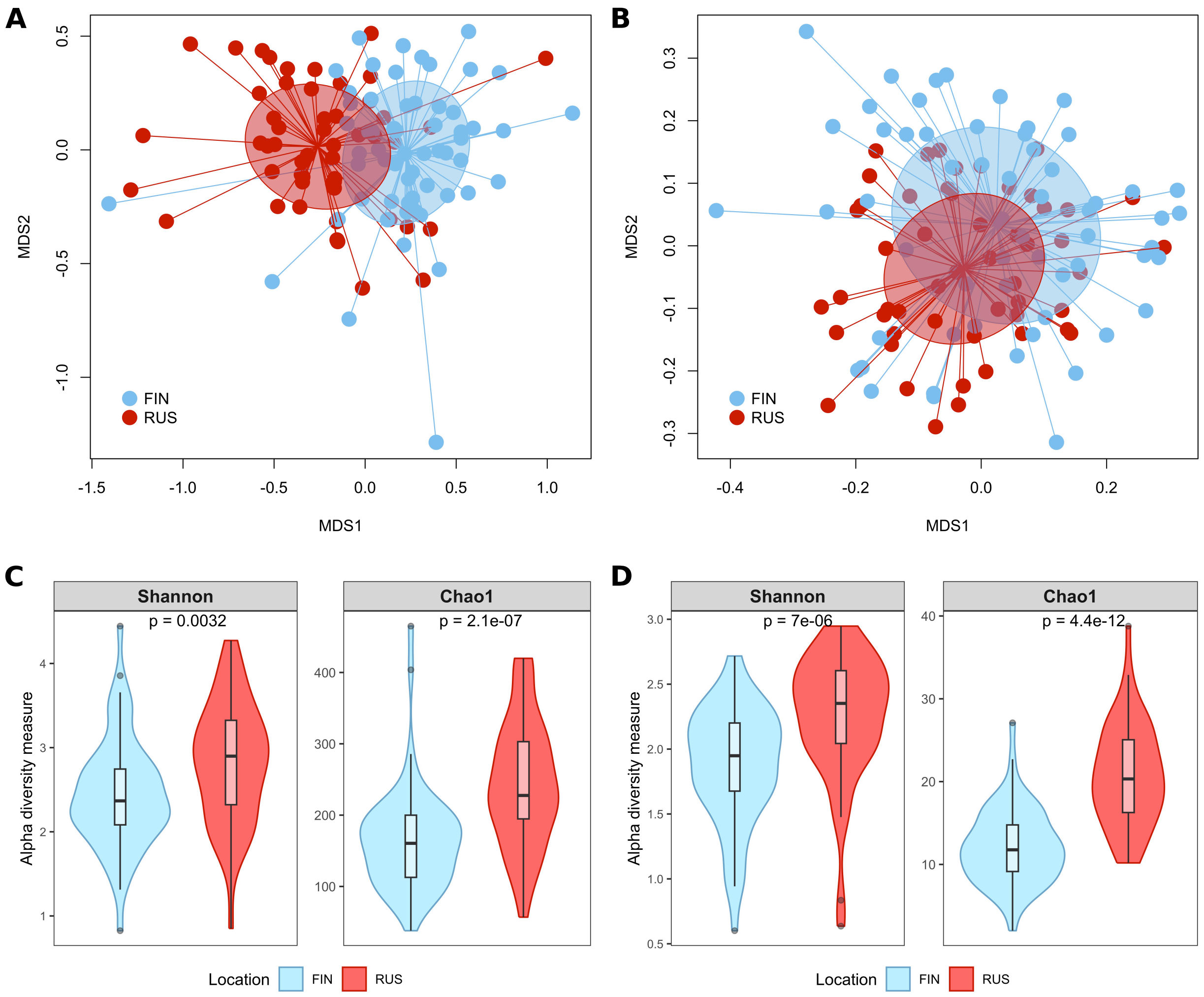

Figure S6. Microbiome composition and diversity differ between Finnish and Russian Karelia. (A,C) Differences regarding bacterial, eukaryotic and archaeal microorganisms and (B,D) in viral profiles including (A,B) non-metric multidimensional scaling on sample-wise Bray-Curtis dissimilarities, stress value (A) 0.13 and (B) 0.2. (C,D) Shannon diversity and the Chao1 species richness estimate of (C) microbial species-level genome bins and (D) viral species. P-value was calculated using Wilcoxon rank-sum test.

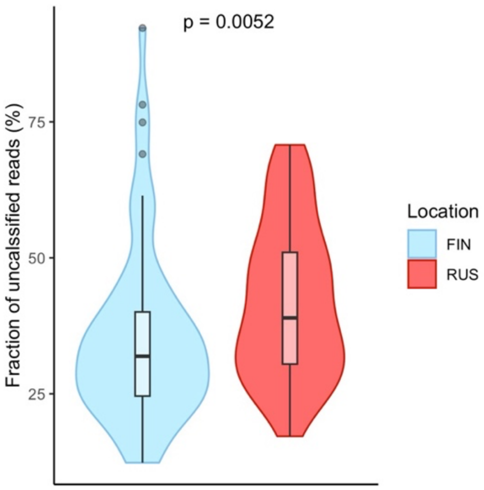

Figure S7. Fraction of unclassified reads in samples from Finnish and Russian Karelia. Fraction of reads that were not classified as *Bacteria, Archaea* or *Eukaryota* using the MetaPhlAn 4 taxonomic profiler. *P*-value was calculated using Wilcoxon rank-sum test.

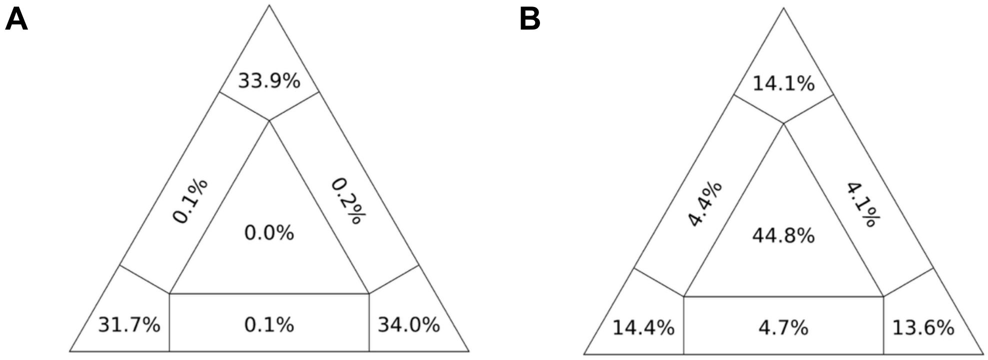

Figure S8. Likelihood-mapping analysis for *Malassezia restricta* and *Cutibacterium acnes* strains. The phylogenetic information contained in MSA of (A) 31 *M. restricta* strains and (B) 105 *C. acnes* strains displayed by likelihood-mapping performed using IQ-TREE 2 with 10000 random quartets. This methodology is based on the analysis of the maximum likelihoods for three fully resolved tree topologies that can be computed for a subset of four sequences (a quartet) from the input MSA. The three likelihoods are represented as one point within an equilateral triangle (the likelihood map) partitioned into different regions. Values in the center of the triangle represent the fraction of unresolved quartet trees, values in the vertices represent the fraction of fully resolved trees, and the values between vertices represent conflicting trees.

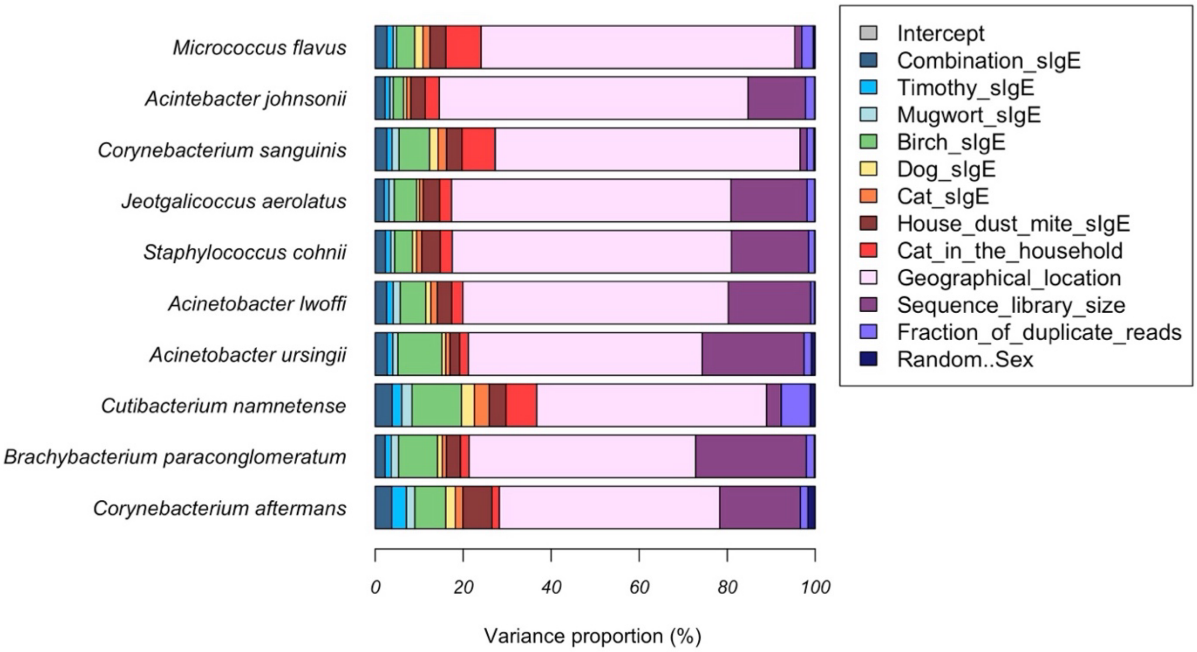

Figure S9. Variance partitioning of species abundances. Hierarchical modelling of species communities (HMSC) was utilized to identify abundant species (present in at least 50% of the samples) associated with geographical location. In addition to geographical location, serum Ige levels specific for a combination of eight inhalant allergens, timothy, mugwort, birch, dog, cat and house dust mite, as well as cat ownership, library size and fraction of duplicate reads, were included as explanatory variables in the model. Sex was included as random effects. Species with > 50% of abundance variation explained by geographical location were considered associated with the variable (n = 10).

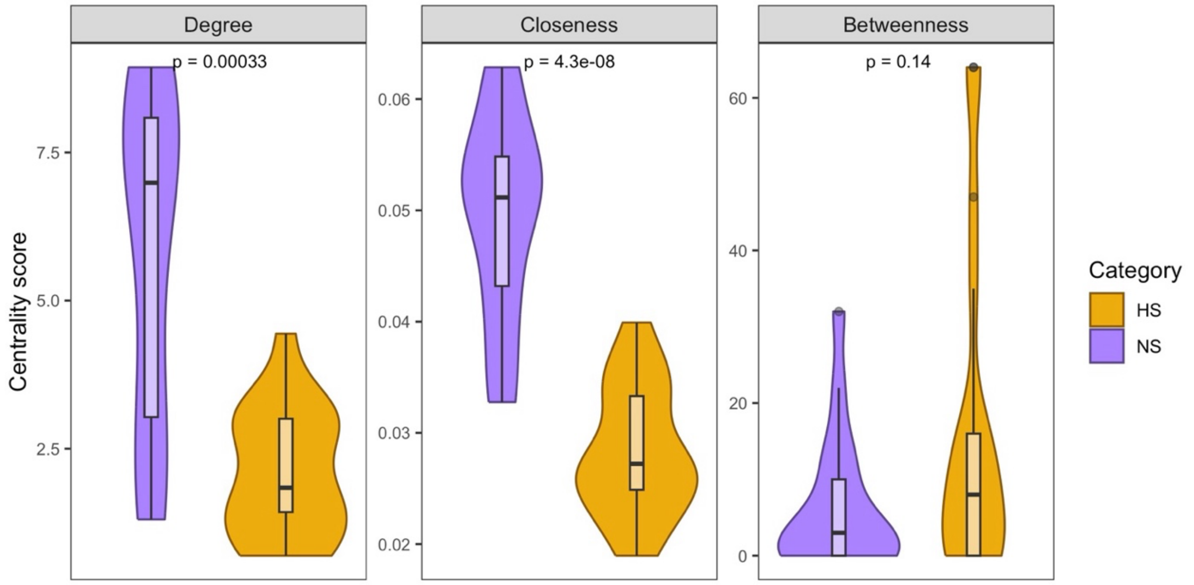

Figure S10. Comparison of network centrality scores between the NS and HS networks. Weighted degree, closeness and betweenness centrality scores were compared between the NS and HS networks. Weighted degree centrality depicts the per node number of edges and the sum of edge weights. Closeness centrality depicts the per-node path distances to other nodes and betweenness the number of times a node falls within the shortest paths between two other nodes. *P*-value was calculated using Wilcoxon rank-sum test.

3. References

1. Bowers, R.M., et al., *Impact of library preparation protocols and template quantity on the metagenomic reconstruction of a mock microbial community.* BMC Genomics, 2015. **16**: p. 856.
2. Tourlousse, D.M., et al., *Characterization and Demonstration of Mock Communities as Control Reagents for Accurate Human Microbiome Community Measurements*. Microbiol Spectr, 2022. **10**(2): p. e0191521.
3. Turner, D., A.M. Kropinski, and E.M. Adriaenssens, *A Roadmap for Genome-Based Phage Taxonomy.* Viruses, 2021. **13**(3).
